# Spatio‐temporal shifts in British wild bees in response to changing climate

**DOI:** 10.1002/ece3.10705

**Published:** 2023-11-16

**Authors:** Chris Wyver, Simon G. Potts, Mike Edwards, Rowan Edwards, Deepa Senapathi

**Affiliations:** ^1^ Centre for Agri‐Environmental Research, School of Agriculture, Policy and Development University of Reading Reading UK; ^2^ Bees, Wasps and Ants Recording Society, Leaside, Carron Lane West Sussex UK

**Keywords:** climate change, distribution modelling, phenology, spatio‐temporal shifts, wild bees

## Abstract

Climate plays a major role in determining where species occur, and when they are active throughout the year. In the face of a changing climate, many species are shifting their ranges poleward. Many species are also shifting their emergence phenology. Wild bees in Great Britain are susceptible to changes in climatic conditions but little is known about historic or potential future spatio‐temporal trends of many species. This study utilized a sliding window approach to assess the impacts of climate on bee emergence dates, estimating the best temperature window for predicting emergence dates for 88 species of wild bees. Using a ‘middle‐of‐the‐road’ (RCP 4.5) and ‘worst‐case’ (RCP 8.5) climate scenario for the period 2070–2079, predictions of future emergence dates were made. In general, the best predicting climate window occurred in the 0–3 months preceding emergence. Across the 40 species that showed a shift in emergence dates in response to a climate window, the mean advance was 13.4 days under RCP 4.5 and 24.9 days under RCP 8.5. Species distribution models (SDMs) were used to predict suitable climate envelopes under historic (1980–1989), current (2010–2019) and future (2070–2079 under RCP 4.5 and RCP 8.5 scenarios) climate conditions. These models predict that the climate envelope for 92% of studied species has increased since the 1980s, and for 97% and 93% of species under RCP 4.5 and RCP 8.5 respectively, this is predicted to continue, due to extension of the northern range boundary. While any range changes will be moderated by habitat availability, it highlights that Great Britain will likely experience northward shifts of bee populations in the future. By combining spatial and temporal trends, this work provides an important step towards informing conservation measures suitable for future climates, directing how interventions can be provided in the right place at the right time.

## Graphical abstract

Climate change is driving spatio‐temporal shifts in many taxa, however the effects on British wild bees have not been quantified. We show that bees are emerging earlier in warmer years, and are predicted to continue to do so. Many bees have already shifted their distributions northwards, and are likley to continue to do so under future climate conditions.

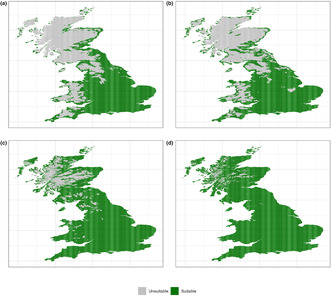

## 1 INTRODUCTION

Wild bees in Great Britain comprise over 270 species (Falk, 2019). Many of these species provide important pollination services to numerous crops widely grown in Great Britain (Breeze et al., 2011; Hutchinson et al., 2021). They are expected to need to provide this service to an even greater extent in the future as the area of land cultivated with pollinator‐dependent crops continues to increase (Aizen et al., 2019). Although the majority of crop pollination is carried out by a very small proportion of the overall bee fauna (Hutchinson et al., 2021; Kleijn et al., 2015), widespread reports of declines in many species of wild bees in the United Kingdom (Biesmeijer et al., 2006; Powney et al., 2019) mean that this important ecosystem service is potentially under threat. Pollination deficits are already being reported in apple crops (Garratt et al., 2014), and other crops such as sweet cherry, blueberry and highland coffee show increased yield when visited by insect pollinators (Klein et al., 2003; Nicholson & Ricketts, 2019; Osterman et al., 2023). Additionally, wild bees contribute to the pollination of many non‐crop flowering plants, up to 87.5% globally (Ollerton et al., 2011). This includes many rare and threatened flowering plants in Great Britain, such as the late flowering *Gentianopsis ciliata*, classed as Critically Endangered in England (Stroh et al., 2014), but highly dependent on insect pollination to produce seeds, likely to be carried out by bumblebees (Oostermeijer et al., 2002).

There are a range of threats to wild bees and their associated pollination service. One of the major threats is climate change (Dicks et al., 2021), which has been shown to alter both spatial (i.e. range boundaries) (Nooten & Rehan, 2020) and temporal (i.e. activity periods) (Bartomeus et al., 2011) distributions of wild bees. Historically, bumblebee species show mixed spatial responses to climate change in the United Kingdom, with common bumblebees generally becoming more widely distributed, and rarer bees seeing range contractions, although these trends appear to have stabilized in recent years (Casey et al., 2015). Despite these historic changes, relatively little data exists on potential future climate‐driven changes in wild bee distributions in Great Britain. Reports from the United States predict widespread range losses of bumblebees under future climate conditions, with gains at more northerly latitudes and losses in the south (Sirois‐Delisle & Kerr, 2018). In many cases, in both the United States and Europe, poleward range gains are not keeping pace with equatorward range losses, effectively placing many species in a ‘climatic vice’ (Kerr et al., 2015). With Great Britain sitting at the northern edge of many wild bee species ranges (Ollerton et al., 2014), and the projections of northwards movement shown in the United States and expected in Great Britain, climate change could present an opportunity for wild bees to see range expansions in Great Britain.

Climate, however, is not the only determinant of species ranges. Habitat fragmentation and loss of habitat through land use change are also contributing to changes in the distributions of wild bee species in the United Kingdom (Senapathi et al., 2015). This is largely credited with large‐scale changes in agricultural policy and practices linked to agricultural expansion and intensification in the periods post‐First and Second World Wars (Raven & Wagner, 2021). Indeed, significant declines in bumblebees in Great Britain were seen in the post‐World War II period. Although these declines appeared to have slowed in recent decades the resultant communities are much more homogenized as many of the most sensitive species have been lost (Carvalheiro et al., 2013; Powney et al., 2019). More recently, the improvement of land considered economically unviable for agricultural production (Ollerton et al., 2014) has threatened bee populations. These changes in agricultural practices, such as increased use of mechanization and synthetic fertilizers and pesticides, coupled with increasing adoption of monocultures and loss of boundary features are likely to have contributed to rapid local extinctions of wild bees and wasps (Powney et al., 2019). This simplification of agricultural landscapes also reduces the amount of available forage for those species that persist, exacerbating risk, especially for species that do not forage on flowers found in these crop monocultures, such as Rosaceae, Brassicaceae and Asteraceae species (Scheper et al., 2014).

Alongside the spatial changes, many species of wild bees in Britain and further afield are also experiencing earlier emergence dates over time, and these advances are linked to warming climates (Bartomeus et al., 2011; Wyver et al., 2023). These shifts are species‐specific, but at least in part explained by life‐history traits, namely nesting habits (spring emerging above‐ground nesters showed greater phenological sensitivity to climate change than below‐ground nesters) and general activity period (spring emergers generally experienced phenological advancements whereas autumn emergers generally experienced delays) (Dorian et al., 2022). The major drivers of these changes are known to be temperature (Bartomeus et al., 2011; Wyver et al., 2023) and/or rainfall (Stemkovski et al., 2020). The exact mechanisms controlling this process are not fully understood, although it is likely linked to increased metabolic rates, especially under increased temperatures (Fründ et al., 2013). Usually, however, the climate window chosen to test for phenological shifts is often the same for all species (i.e. ‘spring’ or ‘April’) and may not be directly relevant to the ecology of each species. This approach may fail to identify a window of greater sensitivity which could provide a better estimate of phenological sensitivity to climate change.

These combinations of spatial and temporal changes pose clear threats, but also potential opportunities for both species' persistence and the pollination services they provide. To minimize the threats and maximize opportunities, policymakers and land managers have a range of response options that can provide suitable habitats and resources for wild bees. In the United Kingdom, encompassing Great Britain and Northern Ireland, 69% of all land is classed as utilized agricultural area (DEFRA, 2022b), encompassing arable, horticultural and pastoral land, and as a result, perhaps the largest opportunity for providing for wild bees comes from biodiversity‐friendly management of farmland. These schemes often provide benefits to pollinators by including interventions such as sowing or managing flower‐rich habitats and non‐crop plants, and reductions in agricultural inputs (e.g. England's Environmental Land Management Scheme, ELMS). Flower‐rich interventions have been shown to locally benefit some groups of pollinators, dependent on the diversity of non‐crop plants present (Carvell et al., 2007; Crowther & Gilbert, 2020; McHugh et al., 2022), which currently may not be optimal for promoting bee diversity (Wood et al., 2015). Whilst evidence suggests that agri‐environment schemes may provide some benefits to pollinators (Breeze et al., 2014), it is likely that these benefits are not being maximized due to limited considerations of target species phenology, especially for floral interventions (Image et al., 2022; Timberlake et al., 2019).

Additionally, management for wild bees can take the form of protected areas, and in Great Britain, these come at a range of scales ranging from regional (e.g. National Parks) to local (e.g. local nature reserves). With a finite budget for nature conservation in the United Kingdom (Great Britain and Northern Ireland), £624 million in public money and £243 million of private sector money attributed to biodiversity protection in 2020/2021 (DEFRA, 2022a), maximizing value for money by incorporating both spatial and temporal ecology of wild bees, targeting the most beneficial areas, with the most beneficial implementations at the most beneficial time is crucial to ensure bees can persist and provide pollination services to both crops and wildflowers.

To effectively do this, understanding where and when bees currently occur, where and when they could potentially occur under future climate scenarios, is vital to understanding where and when management interventions are needed. In Great Britain, there is an extensive database of where bees occur, curated by the Bees, Wasps and Ants Recording Society (BWARS), however, for many species, climate suitability modelling has not been undertaken, with notable exceptions studied by Polce et al. (2014), who used Species Distribution Models (SDMs) to assess changes in the spatial overlap between apple crops and their pollinators between current and 2050 climates. This study predicted possible changes in the ranges of bees (increases for 20 species and contractions for 10 species), and ultimately a potential decline in the spatial overlap between apple orchards and their pollinators by 2050. Additionally, utilizing SDMs to predict future ranges and activity periods can help conservation planners to forward plan for specific goals, either to prevent further loss, attract potentially suitable species or simply maintain or improve the pollination service provided by a wild bee community.

This study looks to combine both spatial and temporal trend analyses of bee populations by asking:

1. What temperature window (generated through sliding window analysis) best predict bee emergence dates, and what might emergence dates look like under future climate scenarios?
2. What are the current climate envelopes of British wild bee species, and how are these projected to change under a future climate scenario?

## 2 METHODS

### 2.1 Bee data

Bee data was obtained from the Bees, Wasps and Ants Recording Society (www.bwars.com) within Great Britain. This is a dataset comprised of opportunistic records, each with a species, recording date and location. Although there is no formal protocol, records must meet a data quality threshold, where the data is checked by experts within BWARS for taxonomic accuracy for inclusion. Data were extracted for the period 1980–2019.

A species was eligible for inclusion in analysis provided it had 20 or more years of records, with each year containing a minimum of 20 records (Wyver et al., 2023). This resulted in a total of 88 (out of a potential total of 270) species being available for analysis. A full list of species can be found in Tables S1–S7. Emergence dates of each of these *species x year* combinations were calculated as the 5th percentile flight date, taken as being 5% of the distance between the first and last recorded observations, and is independent of abundance of records. For univoltine species (species with one generation per year), this was simply taken as the 5th percentile of all records for any given year. For bivoltine species (two generations per year), or species exhibiting variable voltinism throughout the study area (partial second generation in some years), a k‐means clustering method was used to identify records belonging to the first generation. Only records shown to be in the first generation were used in the calculation of the 5th percentile flight date in these instances. Outlying emergence dates were identified for each species individually, using the interquartile range (IQR) method (Barbato et al., 2011), whereby the IQR is calculated as the range between the 25th (Q_1_) and 75th (Q_3_) percentile values. Values lower than Q_1_ − 1.5*IQR or higher than Q_3_ + 1.5*IQR were removed.

### 2.2 Temporal shifts

#### 2.2.1 Sliding window analysis

To overcome the often‐arbitrary selection of the best predicting climate window, a sliding window approach using the r package ‘*Climwin*’ (Bailey & Van De Pol, 2016) was implemented. This approach allows for all climate windows within a set range to be tested, and allows for fine‐resolution data, in this case daily mean temperature, to be used.

Historic daily temperature data came from the e‐Obs dataset (v26.0) (Cornes et al., 2018) at 0.25° × 0.25° gridded resolution. Data for all grid squares covering Great Britain were extracted and averaged to generate mean daily temperature for the study region.

An absolute window was selected, and the reference day was set as the mean date of emergence for each species across the whole study period. Possible time windows were restricted to allow the timing of the window to fall at any point within the 365 days before the reference date, with a minimum window duration of 14 days. The inclusion of very short climate windows is often not biologically plausible and can produce statistical artefacts (van de Pol et al., 2016).

The best predicting window was chosen as the window with the largest decrease in AICc from the null model, and the randomization function within the climwin package was used to calculate the probability the best predicting window was chosen by chance (‘false positives’). Ten randomizations were used for this purpose, which has been shown to balance a suitable detection rate of false positives and reduce large computing time, as this process is computationally intensive (van de Pol et al., 2016). A climate window was considered a ‘true’ cue if *p*ΔAICc was <.05 (i.e. the probability of such a result occurring in a randomized dataset was <5%). The raw data from the best supported ‘true’ window was extracted, and used in a linear model to assess change in emergence date linked to climate:

$$\text{Emergence Date}\sim {\text{Mean temperature during best}}^{‘}{\text{true}}^{’}\;\text{temperature window}$$

#### 2.2.2 Predicting future emergence

To predict future emergence dates, daily climate projections between 2070 and 2079 were obtained from CMIP5 climate projections under RCP 4.5 and RCP 8.5, also available from the E‐Obs dataset (Cornes et al., 2018) and selected to provide assessments of temporal shifts under a ‘middle‐of‐the‐road’ and ‘worst‐case’ future climate scenario. Mean projected temperature for each of the selected climate windows for the period 2070–2079 was calculated and models were re‐fitted using the *predict()* function in r to generate future emergence dates for both scenarios.

### 2.3 Spatial shifts

#### 2.3.1 Spatial shifts in climate envelopes

Historic and future changes in potential climate envelopes were estimated using models created using MaxEnt version 3.3.4 (Phillips et al., 2008). This a commonly used tool in species distribution modelling (SDM) where presence‐only data, such as that provided by BWARS, are available. Raw bee records were passed through two filter stages to be included in the spatial analysis. Initially, records with imprecise grid coordinates (<1 km scale) were removed, and subsequently, duplicate records within the same species and 1 km square were also removed. The bioclimatic variables used in this analysis are the same as those used in pollinator distribution models in Great Britain (Polce et al., 2013). These were derived using the *‘biovars’* function from the *‘dismo’* package (Hijmans et al., 2017) using maximum and minimum monthly temperature, and monthly precipitation, which were obtained from CHESS‐SCAPE at a 1 km^2^ resolution (Robinson et al., 2023) (Table 1).

**TABLE 1:** Predictors used in the wild bee distribution models.

Predictor	Description
Bio3	Isothermality
Bio7	Temperature annual range
Bio9	Mean temperature of driest quarter
Bio11	Mean temperature of coldest quarter
Bio15	Precipitation seasonality
Bio19	Precipitation of coldest quarter

To create the models for each species, 75% of the data was used for training and 25% for testing. SDMs for each species were run 10 times, using the ‘sub‐sample’ method. The convergence threshold was set to 10^−5^ with 5000 iterations, and with a maximum of 10,000 background points. The selection of the functions for the predictor variables (feature type) was carried out automatically, following the default options depending on the number of occurrences: ‘linear + quadratic + hinge’ if there are from 15 to 79 points (2 species) and ‘all’ if there are >80 points (86 species) (Urbani et al., 2015).

The cloglog output was used, and this provides continuous values for each grid cell from 0 (unsuitable) to 1 (most suitable). These values can be interpreted as the probability of presence of suitable climate conditions for the target species (Veloz, 2009). The ‘10th percentile training presence cloglog threshold’ was selected to covert the continuous score to a binary output. This threshold selects the value above which 90% of the training locations are correctly classified (Zarzo‐Arias et al., 2019). This threshold is a recommended for datasets collected with non‐standardized methods or by different collectors or observers over a long time, as the BWARS dataset is, (Rebelo & Jones, 2010; Urbani et al., 2015) and is commonly used in SDM exercises (e.g. Barik et al., 2022; Crawshaw et al., 2022; Segal et al., 2021).

Validation of SDMs was done by testing whether the area under the curve (AUC) of the receiver operating characteristic (ROC) significantly differed from a random expectation using bias‐corrected null models (Raes & Ter Steege, 2007). Ninety‐nine null models were created for each species, run using the same MaxEnt settings, except the random test percentage, which was set to 0. with the number of ‘records’ equal to the actual number of records of each species. These were drawn randomly without replacement from a list of grid squares containing records for the whole dataset, to account for potential geographic sampling bias. If a species observed AUC (mean of 10 replicate runs) ranked above the upper 95% confidence interval of the null models (above the 95th highest AUC value of the 99 null models), then the modelled distribution was considered significant, with a <5% chance that a random set of records could produce an equally good model (Table S2).

#### 2.3.2 Predicting historic and future climate envelopes

To test for changes in climate envelopes, each SDM that proved better than a random set of records was re‐fitted, with future bioclimatic variables for the period 1980–1989 and 2070–2079 (under both RCP 4.5 and RCP 8.5), again obtained from CHESS‐SCAPE. Change in the climate envelope was calculated as the change in the number of grid cells classed as suitable. Finally, to test for the movement of climatically suitable area, the latitude of the northern range boundary (90th percentile latitude), range centroid (50th percentile latitude) and southern range boundary (10th percentile latitude) of the climate envelope was calculated under historic, current and future climate conditions, and the distance and bearings between the historic and current, and current and future locations were calculated using the ‘*geosphere*’ package (Hijmans et al., 2019).

## 3 RESULTS

### 3.1 Temporal shifts

#### 3.1.1 Sliding window analysis

**FIGURE 1:**
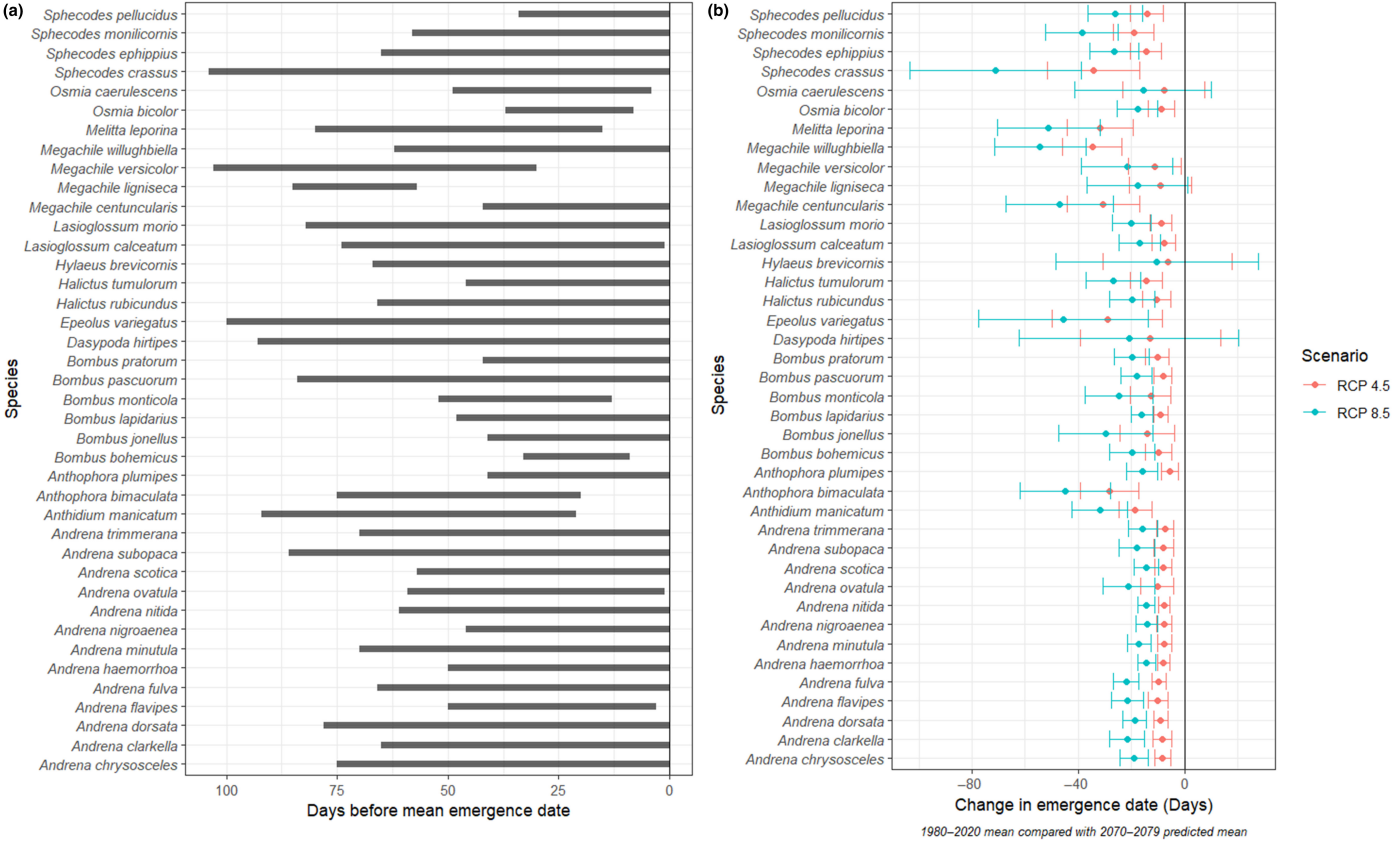
(a) Locations of best predicting climate windows for wild bee emergence. All windows presented here were statistically unlikely to occur by chance (*p*ΔAICc < .05), and linear models of emergence date regressed against mean temperature during the highlighted window show a significant effect. (b) Predicted shift in emergence between current (1980–2020) and future (2070–2079) emergence dates. Negative values indicate advancement of emergence dates. Bars indicate 95% confidence intervals.

In total, 40 of the 88 (45.5%) species showed a significant phenological response to a ‘true’ climate window (i.e. a climate window, greater than 14 days, that performed better than a null model and *p*ΔAICc < .05). The timing of these windows generally was within 0–3 months of the mean date of emergence for each species (Figure 1a, Table S3).

All 40 species showing significant phenological shifts related to temperature experienced earlier emergence dates in warmer years. These ranged from 4.1 ± 1.1 (*Halictus rubicundus*, *p* < .001) to 14.2 ± 3.2 (*Sphecodes crassus*, *p* < .001) days per °C temperature increase during the best‐explaining temperature window (Table S4).

#### 3.1.2 Potential changes in future phenology

For the 40 species that showed a significant phenological response to a climate window, potential emergence dates under ‘middle‐of‐the‐road’ (RCP 4.5) and ‘worst‐case’ (RCP 8.5) scenarios for the period 2070–2079 were estimated. All 40 species are projected to emerge earlier in the future under both climate scenarios, compared with baseline (1980–2020) dates. Under RCP 4.5, changes range from a 5.6 ± 3.1‐day advance (*Anthophora plumipes*) to a 34.7 ± 11.2 day advance (*Megachile willughbiella*). Under RCP 8.5, changes range from a 14.2 ± 4.1‐day advance (*Andrena nigroaenea*) to a 54.4 ± 17.2 day advance (*M. willughbiella*) (Figure 1b, Table S5).

### 3.2 Spatial shifts

#### 3.2.1 Current distribution of suitable climate envelope

**FIGURE 2:**
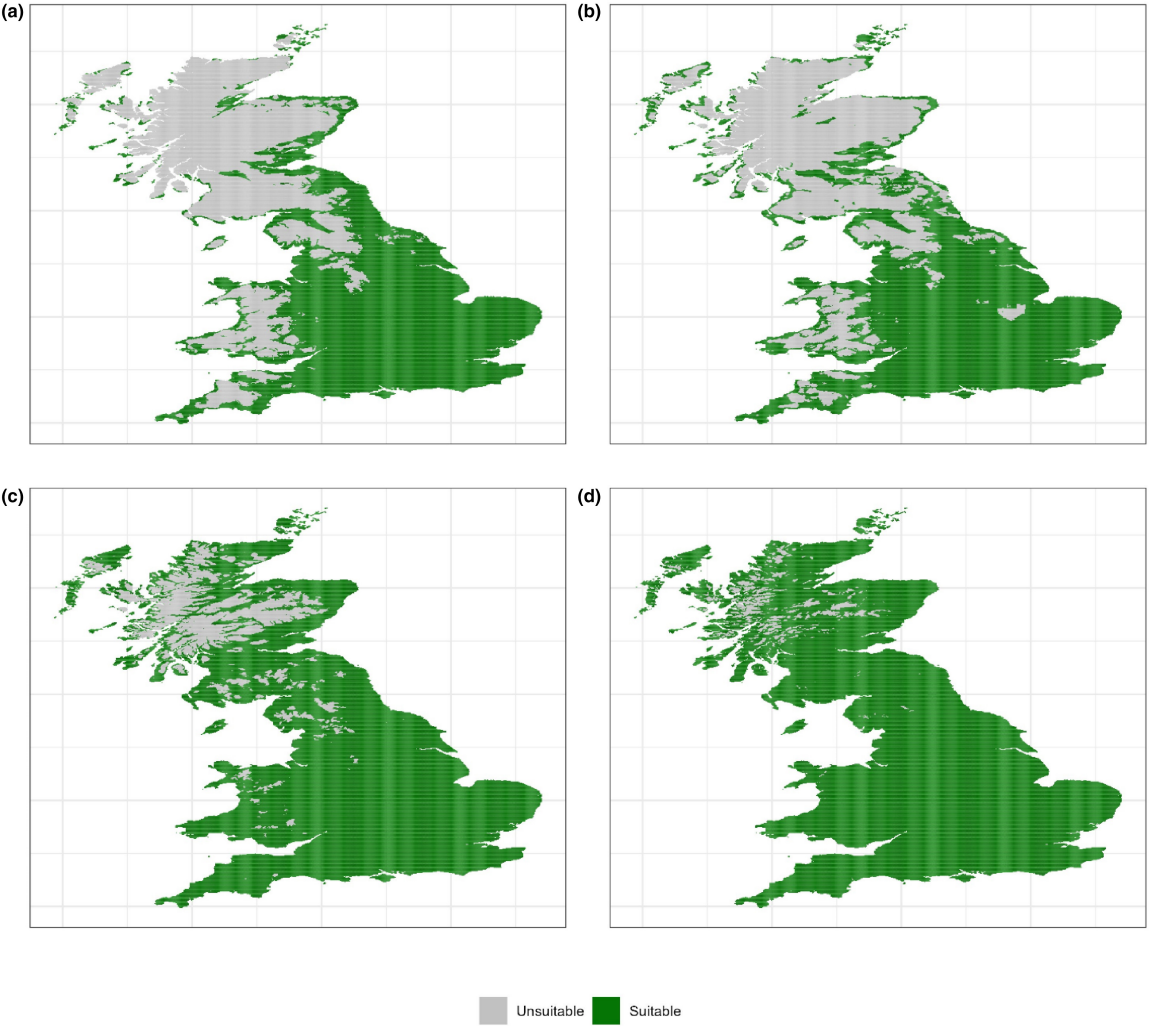
MaxEnt climate maps for *Bombus hortorum*. Showing climate envelope for 1980–1989 (a), 2010–2019 (b) and 2070–2079 under RCP 4.5 (c) and RCP 8.5 (d). 10th percentile training presence threshold = 0.3167. Plots for all other species can be found in Figure S1.

SDMs for 76 of the species performed significantly better than the bias‐corrected null models and were used in further analysis (Table S2). This included both rare and widespread species ranging from *Bombus distinguendus* (2489 grid squares, classed as suitable climate under current climate conditions) to *Bombus hortorum* (Figure 2) (80,300 pixels classed as suitable climate under current climate conditions). The predicted accuracy of models was high, with the mean AUC across all 76 species at 0.860.

#### 3.2.2 Predicted changes in suitable climate envelopes

Of the 76 species with SDMs significantly better than the bias‐corrected null models, the area of the suitable climate envelope increased between the 1980s and 2010s for 91% of species, with the mean climate envelope being 43.6% smaller in the 1980–1989 period compared with the 2010–2019 period.

**FIGURE 3:**
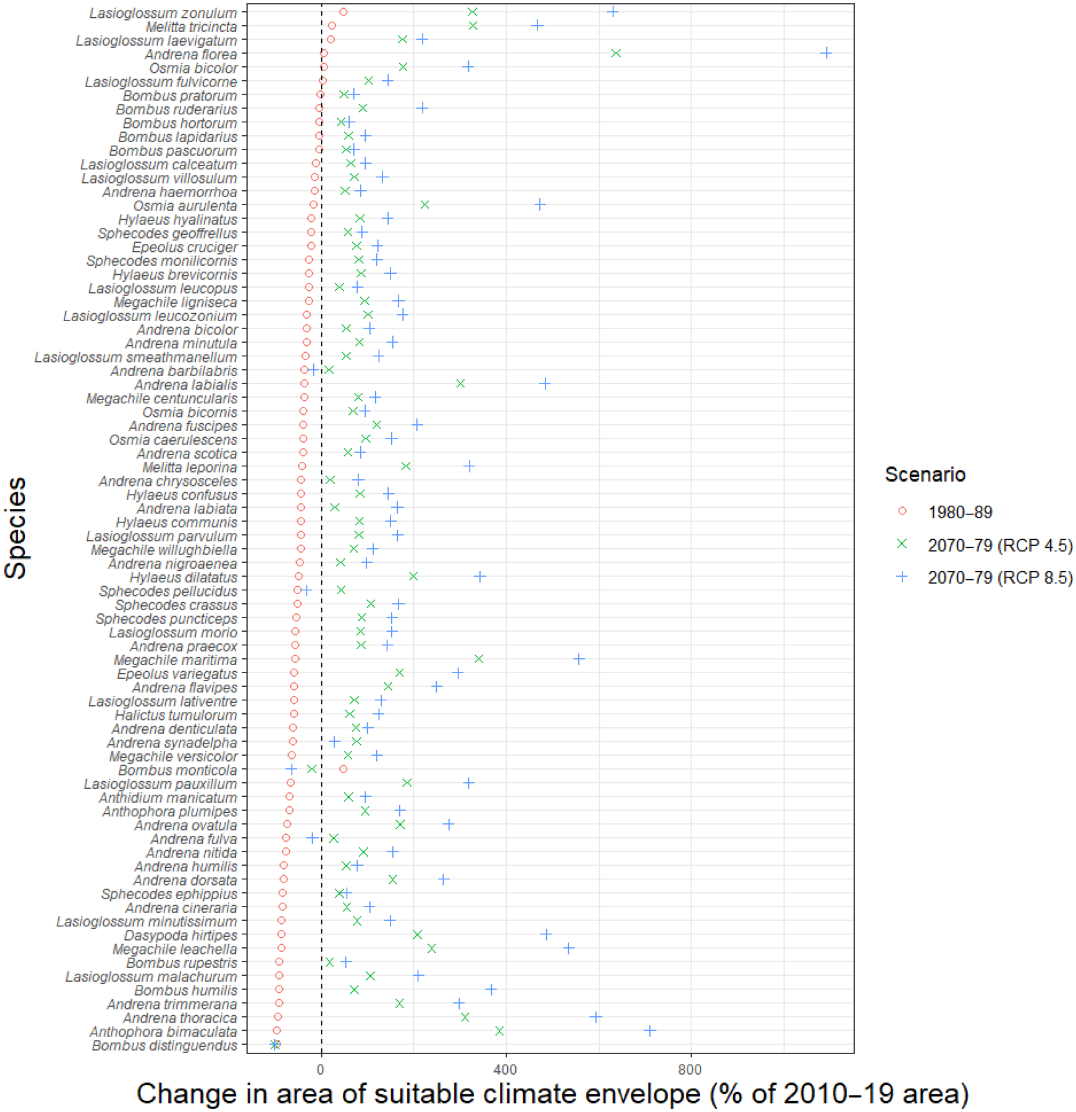
Predicted change in area of suitable climate envelope under historic and future climate scenarios. 0 represents the climate envelope under current (2010–2019) conditions.

The climate envelope of was predicted to continue to increase under both RCP 4.5 and RCP 8.5 into the 2070–2079 period for 74 and 71 species, respectively. Under RCP 4.5 the mean climate envelope increased in size by 113% in the 2070s compared with the present day, and these changes ranged from a 637% increase (*Andrena florea*) to a 100% decrease—complete loss of climate suitable for persistence (*B. distinguendus*). Under RCP 8.5, the mean increase in climate envelope area was 200% in the 2070s compared with present day conditions. The same species showed the largest positive and negative potential changes (1091% increase for *A. florea* and 100% decrease for *B. distinguendus*) (Figure 3, Table S6).

**FIGURE 4:**
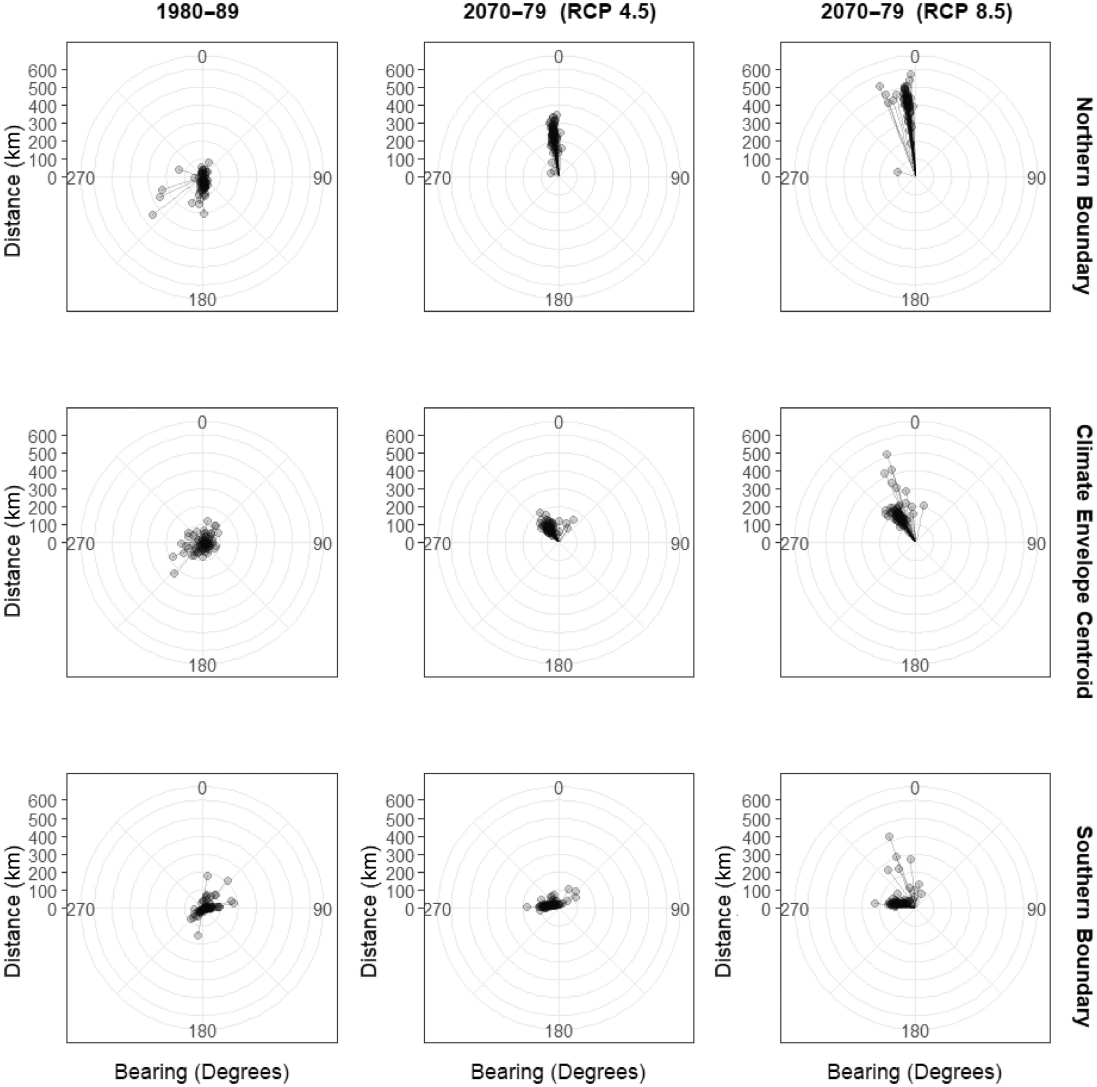
Distance and bearing of shifts in northern climate envelope boundary, climate envelope centroid and southern climate envelope boundary area under historic and future climate conditions. The centre of each plot indicates the position during the current period, and the points indicate the position during the historic or future period.

There was clear evidence of poleward movement of species climate envelopes, with the northern range boundary of most species' potentially suitable climate envelope shifting northwards. Between the 1980s and current period, the northern range boundary shifted northwards by an average of 29.4 km, and between the current period and the 2070s, the current period, the northern range boundary shifted by a mean of 206.0 km (RCP 4.5) and 371.4 km (RCP 8.5) (Figure 4, Table S7).

For most species, the southern range boundary stayed relatively static between the 1980s to the current period (mean = −5.0 km) and the current period to the 2070s under both RCP 4.5 (mean = 18.5 km) and RCP 8.5 (mean = 44.0 km). However, for *Bombus monticola*, which shows a reduced climate envelope under RCP 4.5 and the four species showing reduced climate envelopes under RCP 8.5 (*B. distinguendus* is predicted to have no suitable climate range under both scenarios, so is not included in this group), the southern range boundary moves northwards at a much greater rate than their northern range boundary. *B. monticola*, for example, sees its southern range edge move northwards by 26.4 and 243.4 km under RCP 4.5 and RCP 8.5 respectively, whereas the northern range boundary moves northwards by less—17.4 km under RCP 4.5 and 27.9 km under RCP 8.5, indicating that these species may be caught in a climatic vice in the future. Climate envelope maps for each species can be found in Figure S1.

## 4 DISCUSSION

### 4.1 Temporal shifts

This study presents the first quantitative analysis of the projected spatial and temporal changes of British wild bees and provides important insights into the impacts of worst‐case future climate change on their phenology and distribution. The study found that many bee species analysed (45.5%) showed a significant phenological response to a temperature window. Additionally, potential emergence dates for all 40 species that responded to climate windows were projected to advance (mean = 13.4 days under ‘middle‐of‐the‐road’ RCP 4.5, 24.9 days under ‘worst‐case’ RCP 8.5) from 2070 to 2079 compared to baseline dates from 1980 to 2020. The phenological aspect of this study, predicting earlier emergence in warmer years, conforms to the general trend of phenological advances found in other studies on wild bees (Bartomeus et al., 2011; Wyver et al., 2023), however, the inclusion of species‐specific climate windows again highlights the individual nature of species responses to climate change.

Using a sliding window analysis to produce species‐specific phenology models shows that in Great Britain, temperatures in the period directly before emergence appear to be the best predictor of emergence dates. This conforms with many studies stating spring temperatures are the main driver of temperate bee emergence phenology (Bartomeus et al., 2011; Gordo & Sanz, 2005). However, even species‐specific models still do not explain all the variation in emergence dates, indicating other unexplained factors are still important to some degree in determining emergence phenology. These unexplained drivers could include winter temperatures. Although not as important as spring temperatures, winter temperatures have been shown to play a role in the timing of the emergence dates of several solitary bee species (Fründ et al., 2013), and so are also a likely source of some unexplained variation from the sliding window models. Additionally, the timing of the end of the previous generations flight season may be one source of this variation and has been shown to influence emergence dates in other studies (Stemkovski et al., 2020).

Microhabitat conditions could also be influencing phenology estimates. The emergence phenology of the codling moth (*Cydia pomonella*) is influenced by microhabitat temperatures (Kührt et al., 2006), and bees could experience a similar phenomenon, emerging earlier in warmer microhabitats. In the case of this study, the emergence dates may be influenced by the proportion of records from different habitats (i.e. an emergence estimate comprising 90% of records from agricultural land may be different from an estimate comprising 90% of records from semi‐natural habitat, providing different emergence estimates despite the same mean temperature). While the BWARS dataset does not incorporate habitat type in its recording structure, making it difficult to test for an effect of microhabitat on emergence phenology in this study, it is plausible that this may be the cause of at least some of the unexplained variation in the phenology models.

### 4.2 Spatial shifts

In terms of spatial shifts, this study provides evidence for significant latitudinal shifts in the climate envelopes of many of the species included in this study. Specifically, an average northward shift of 29.4 km in the northern range boundary across all species. Other studies investigating latitudinal shifts in bee distributions report similar shifts (Aguirre‐Gutiérrez et al., 2016), although the magnitude differs. Aguirre‐Gutiérrez et al. (2016), for example reported a 22 km northward shift in Dutch bees. These differences could be due to a range of factors, for example, we show strong species‐specific variation in shifts, and differential results could be caused by different study species, and in different study areas.

This study also used SDMs to evaluate the current and future distribution of suitable climates for 76 of the 88 total study species. The SDMs were found to have a high level of predicted accuracy, with a mean AUC of 0.860 across all 76 species. The study found that the suitable climate envelope was predicted to increase for almost all species under future climate scenarios (74 species under RCP 4.5 and 71 species under RCP 8.5). The magnitude of change varied between species and climate scenario, ranging from a 637% increase to a 100% decrease (mean = 113% increase) under RCP 4.5 and a 1091% increase to a 100% decrease (mean = 200% increase) under RCP 8.5. While this is consistent with other SDM exercises focusing on bees, which show both range expansions and shrinkages dependent on the species (Kuhlmann et al., 2012; Sirois‐Delisle & Kerr, 2018), the magnitude of predicted range increases are much greater here than in these studies.

The historic and future shifts in climate envelopes presented here also appear similar to many large‐scale studies, such as the Climatic Risk and Distribution Atlas of European Bumblebees (Rasmont et al., 2015). This work predicts the widespread poleward movement of many bumblebee species, with large range expansions in northern Europe, including in Great Britain. Great Britain sits in a potentially advantageous position for many wild bee species, relative to much of mainland Europe, as it is close to the northern boundary of the ranges of many species' geographic ranges (Ollerton et al., 2014). This may be a contributing factor to the large northward shifts seen in this study. For many species it was expected that the northern edge of potentially suitable climate would shift further north under future climate scenarios in relation to temperature moving away from species' minimum thermal tolerances towards more favourable conditions for survival, thus allowing for northward colonization.

Conversely, for species already constrained to northern parts of England and Scotland or high‐altitude areas such as *B. distinguendus* and *B. monticola*, climate change has been projected to leave them with nowhere to migrate within Great Britain, resulting in large net range losses. The SDMs for these two species confirm this projection, with range losses of 100% (i.e. complete loss from Great Brittain) under both climate scenarios for *B. distinguendus* and losses of 20.5% (RCP 4.5) and 64.6% (RCP 8.5) for *B. monticola*. This study focusses primarily on data‐rich species, and therefore rare species, many of which already inhabit marginal habitats are likely to see similar trends to *B. distinguendus* and *B. monticola*.

However, it is important to note that while future climate may allow for many species to expand northwards, they are likely to be constrained by a lack of suitable habitat. Many of the species exhibiting the largest increases in suitable climate envelopes are constrained to specific, often rare, habitats, and climate is a major constraint in shaping distributions in Great Britain. *A. florea*, which exhibited the largest potential increase in suitable climate envelope is narrowly oligolectic, and almost exclusively visits plants from the genus *Bryonia* (Polidori & Federici, 2019), and is therefore constrained to areas where these plants are also present and in practice is extremely unlikely to fill its entire predicted climate envelope.

Species' not filling their full predicted climate envelope will not be unique to *A. florea*. Many other species will be constrained to varying degrees (depending on the habitat specificity of each bee species), by non‐climatic factors. Life‐history traits such as habitat breadth (variety of habitats a species can survive in) can be a key factor in realized range shifts (proportion of climate envelope filled) in both mammals and birds (Estrada et al., 2018) and is likely to play a similar role in realized range shifts of bees, although to date this has not been explicitly tested.

Loss of suitable habitat from existing ranges, or lack of suitable habitat in future climate envelopes means that other biological factors such as dispersal ability, voltinism and lecty could play a role in the colonization of new sites. The maps presented here assumes dispersal ability is unlimited, and a species can fill all suitable habitat, however, in practice, there are often barriers that prevent dispersal ability, for example, large areas of intensive farming decreasing habitat connectivity. This again highlights the importance of considering habitat provision, alongside climate, when planning future conservation strategies.

Reduced dispersal ability has been shown to exacerbate range declines in bumblebees in the United States (Sirois‐Delisle & Kerr, 2018). One of the major barriers to the colonization of new areas is increasing habitat fragmentation and a lack of connecting corridors with adequate resources to allow for survival. Conversion of land to intense agricultural or urban land often provides such fragmentation, and expansion of these land uses, something that was not included in future projections in the MaxEnt models, could lead to species not filling all areas identified as being potentially suitable under future climate conditions. In light of this, it could be that the main conservation priority is to reduce or reverse existing fragmentation, to allow for localized dispersion from existing habitats, rather than attempting to prepare for the long‐distance dispersal of wild bees.

### 4.3 Implications for pollination and conservation

Whether caused by changes in climate, habitat or by a combination, changing spatio‐temporal bee distributions are also likely to have a knock‐on impact on the pollination of many flowering crops and plants. Much of the pollination service, particularly of crops, is predominantly carried out by a very small proportion of species such as *Bombus lapidarius* and *Andrena chrysosceles* (Kleijn et al., 2015), many of which are likely to expand their ranges under future climate scenarios. This could present opportunities for the growth of pollinator‐dependent crops such as apple and oilseed rape. However, studies have also shown that increasing functional complementarity can lead to increased seed set (Frund et al., 2013), increased fruit quality and long‐term storability (Samnegård et al., 2019). The expansion of generalist species may increase competition for floral resources and nesting space, ultimately having a detrimental impact on overall species diversity as sensitive species are replaced in a continuation of the trend found by Powney et al. (2019).

Any assessment of potential benefits to crop pollination services under future climate scenarios needs to be considered in conjunction with potential changes in areas suitable for the growth of pollinator‐dependent crops. Currently, distribution modelling of crops in Great Britain is limited to a restricted range of bioenergy crops (Bellarby et al., 2010), and although beyond the scope of this study, modelling the potential distributions of pollinator‐dependent crops, and their pollinators, many of which are known (Hutchinson et al., 2021), may provide insights into where best to target interventions to boost wild pollinators for crop pollination.

However, this work presents complex patterns of spatial and temporal changes in wild bees, so planning is not as simple as looking at spatial or temporal overlap between crops and pollinators separately. From a crop pollination perspective, it is important to consider spatial and temporal changes together when attempting to identify areas suitable for pollinator‐dependent crops and with high insect pollination potential. This is currently a potential missing link in predicting future crop suitability and is an area recommended for future research.

Projecting spatio‐temporal distributions into the future naturally comes with some uncertainty and should be treated with some caution. There are likely to be obstacles to spatio‐temporal adaptation that are very difficult to predict and may influence the future projections in this study. Species may reach the limits of their phenotypic plasticity or genetic variability to allow them to continue to adapt to changing climate conditions. Species which are unable to overcome such obstacles may, in practice, exhibit different phenological responses, or not fill their climate envelopes as presented here.

In spite of these potential issues, these results highlight the significant impact of future climate change on bee phenology and distribution in Great Britain, with implications for both bee populations and the pollination service they provide. The findings suggest that conservation efforts may need to focus on maintaining suitable local habitats for bee species as they shift their distribution in response to changing climatic conditions.

## AUTHOR CONTRIBUTIONS

**Chris Wyver:** Conceptualization (lead); formal analysis (lead); investigation (lead); methodology (lead); visualization (lead); writing – original draft (lead); writing – review and editing (equal). **Simon G. Potts:** Conceptualization (supporting); investigation (supporting); methodology (supporting); supervision (supporting); writing – review and editing (equal). **Mike Edwards:** Data curation (equal); writing – review and editing (equal). **Rowan Edwards:** Data curation (equal); writing – review and editing (equal). **Deepa Senapathi:** Conceptualization (supporting); formal analysis (supporting); funding acquisition (lead); methodology (supporting); supervision (lead); writing – review and editing (equal).

## ACKNOWLEGEMENTS

The authors would like to thank everyone who contributed to the BWARS recording data set, and to BWARS for allowing access to the data.

## Supporting information


Figure S1:

Tables S1–S7.

## Data Availability

The data that support the findings of this study are openly available in the University of Reading Data Repository at https://doi.org/10.17864/1947.000485.
