# Supplementary material for: Spatio‐temporal shifts in British wild bees in response to changing climate
Source: Ecol Evol. 2023 Nov 16;13(11):e10705. doi: 10.1002/ece3.10705 (PMC10654479; doi:10.1002/ece3.10705)
Supplement: Supplementary file 1 — Figure S1: [file ECE3-13-e10705-s002.pdf]

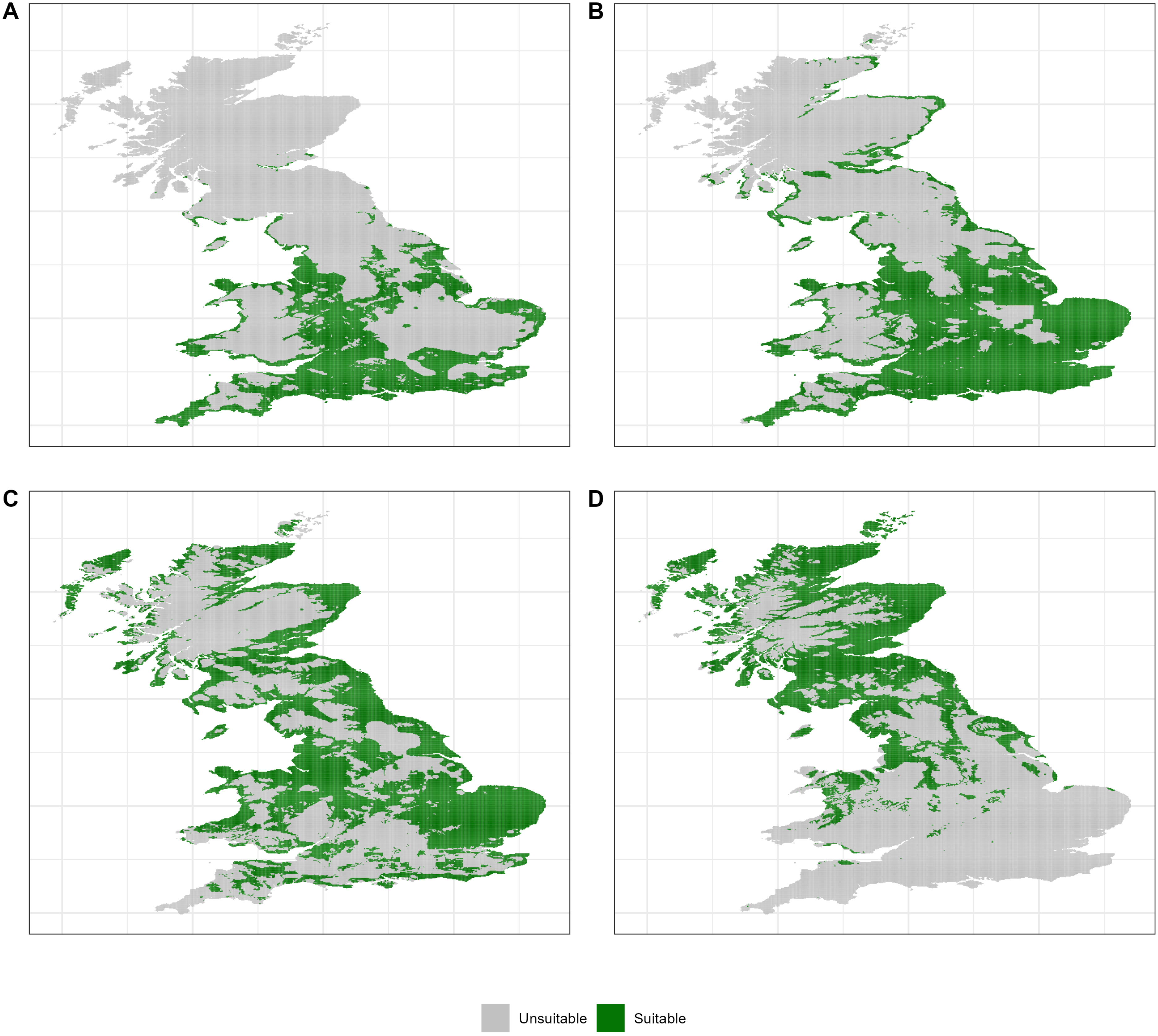

Figure S1. MaxEnt climate envelope maps for *Andrena barbilabris*. Showing climate envelope for 1980-89 **(A)**, 2010-19 **(B)**, and 2070-79 under RCP 4.5 **(C)** and RCP 8.5 **(D)**.
10th percentile training presence cloglog threshold = 0.293

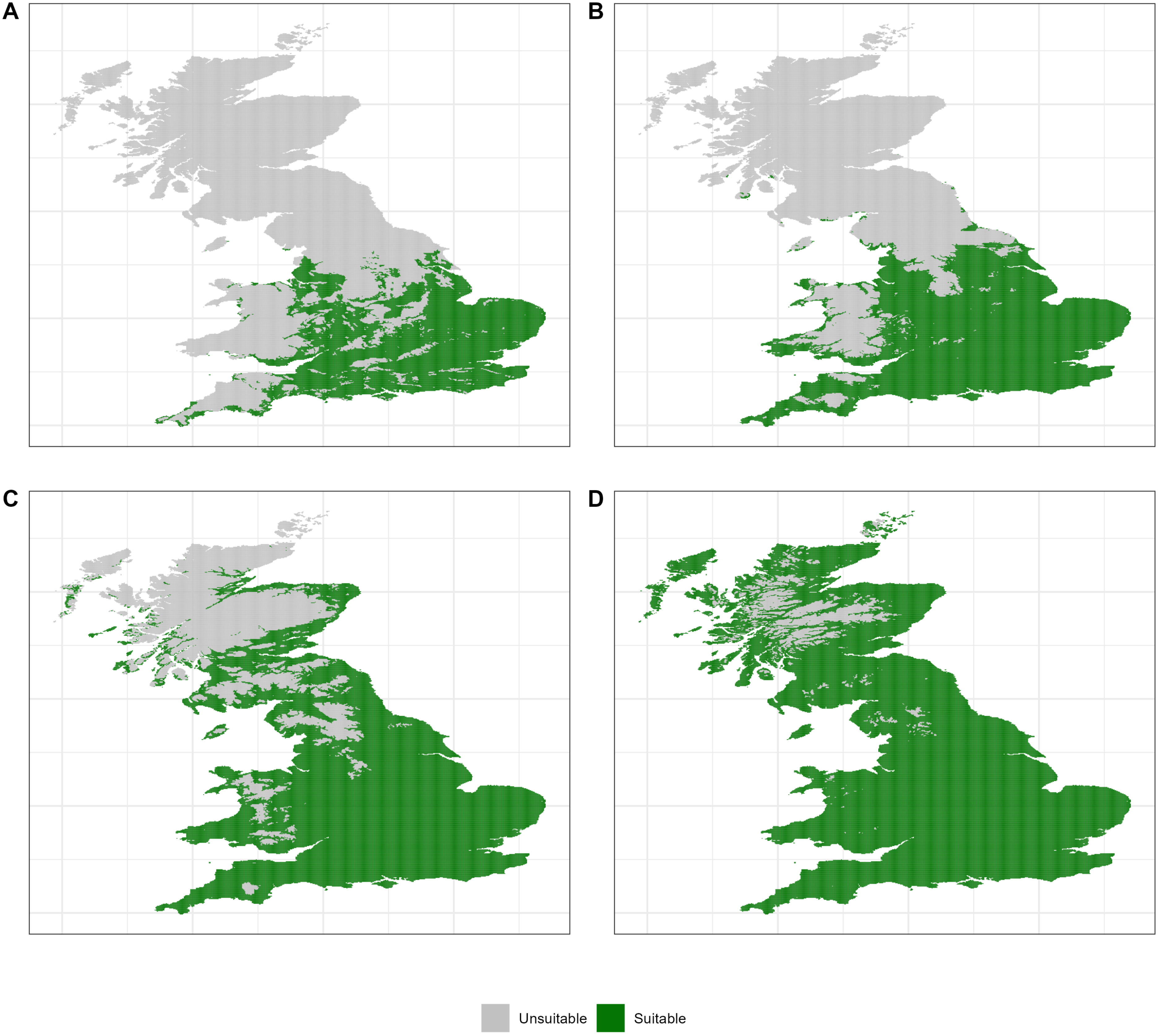

Figure S1. MaxEnt climate envelope maps for *Andrena bicolor*. Showing climate envelope for 1980-89 **(A)**, 2010-19 **(B)**, and 2070-79 under RCP 4.5 **(C)** and RCP 8.5 **(D)**.
10th percentile training presence cloglog threshold = 0.3757

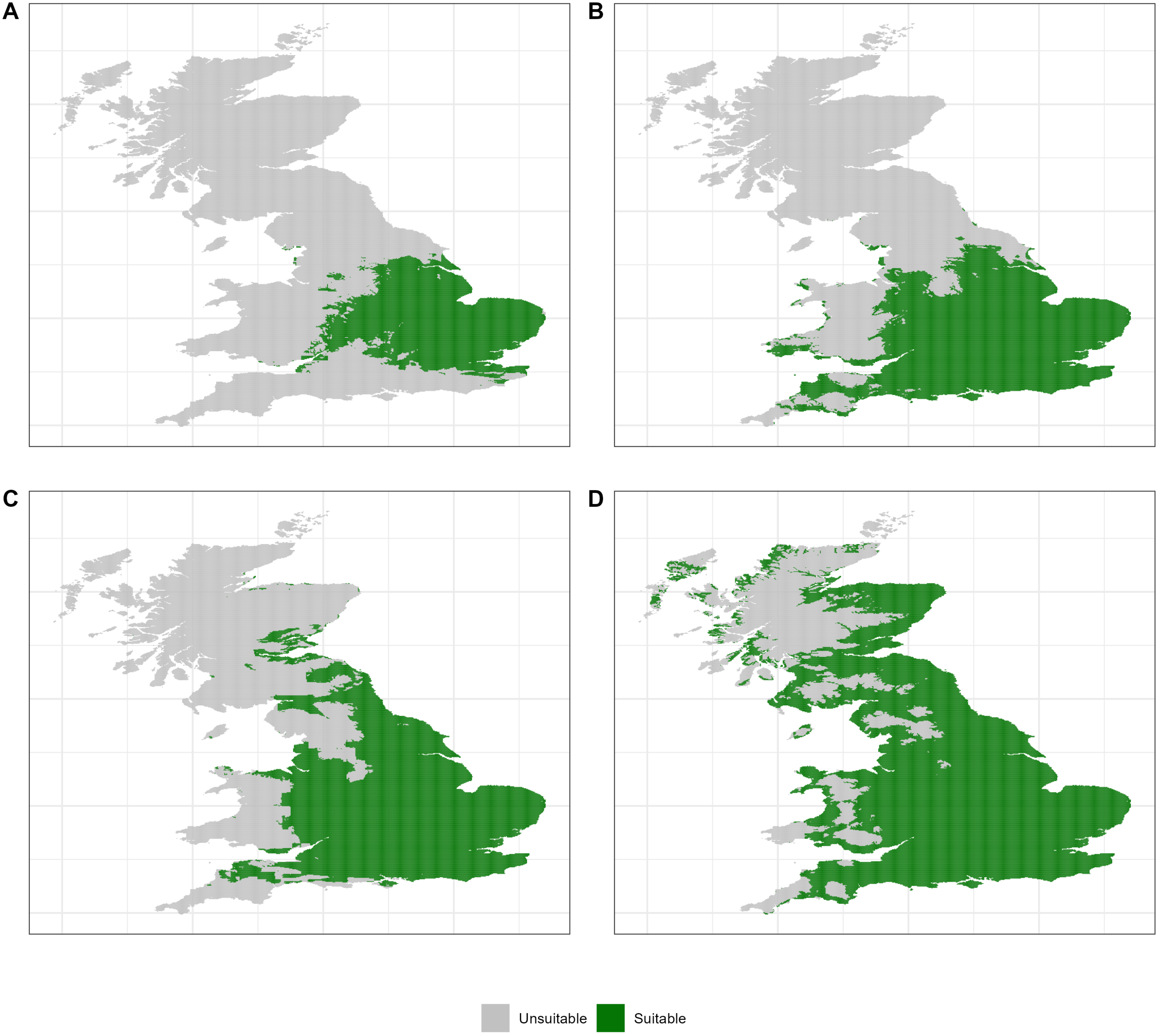

Figure S1. MaxEnt climate envelope maps for *Andrena chrysosceles*. Showing climate envelope for 1980-89 **(A)**, 2010-19 **(B)**, and 2070-79 under RCP 4.5 **(C)** and RCP 8.5 **(D)**.
10th percentile training presence cloglog threshold = 0.3844

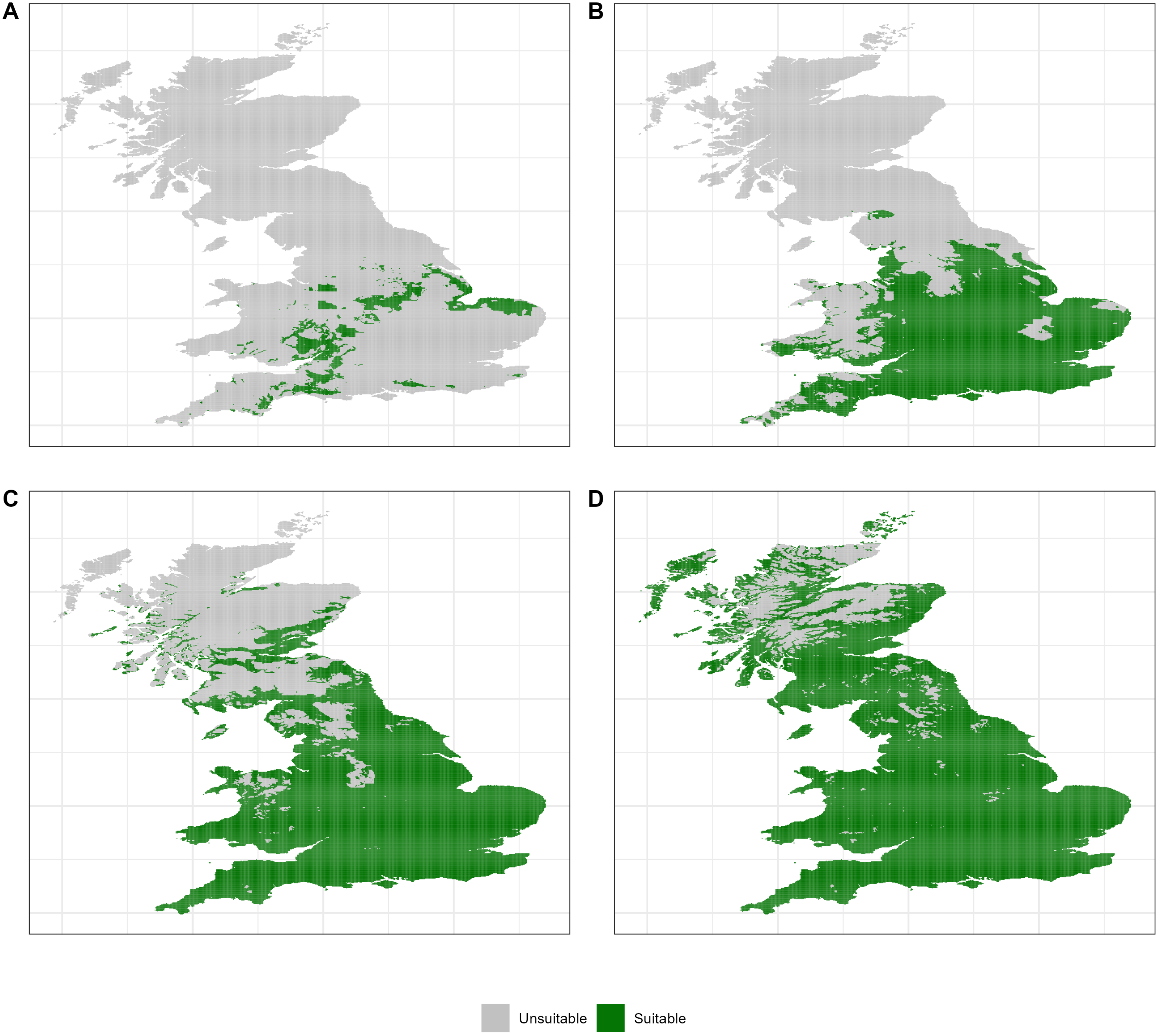

Figure S1. MaxEnt climate envelope maps for *Andrena cineraria*. Showing climate envelope for 1980-89 **(A)**, 2010-19 **(B)**, and 2070-79 under RCP 4.5 **(C)** and RCP 8.5 **(D)**.
10th percentile training presence cloglog threshold = 0.3635

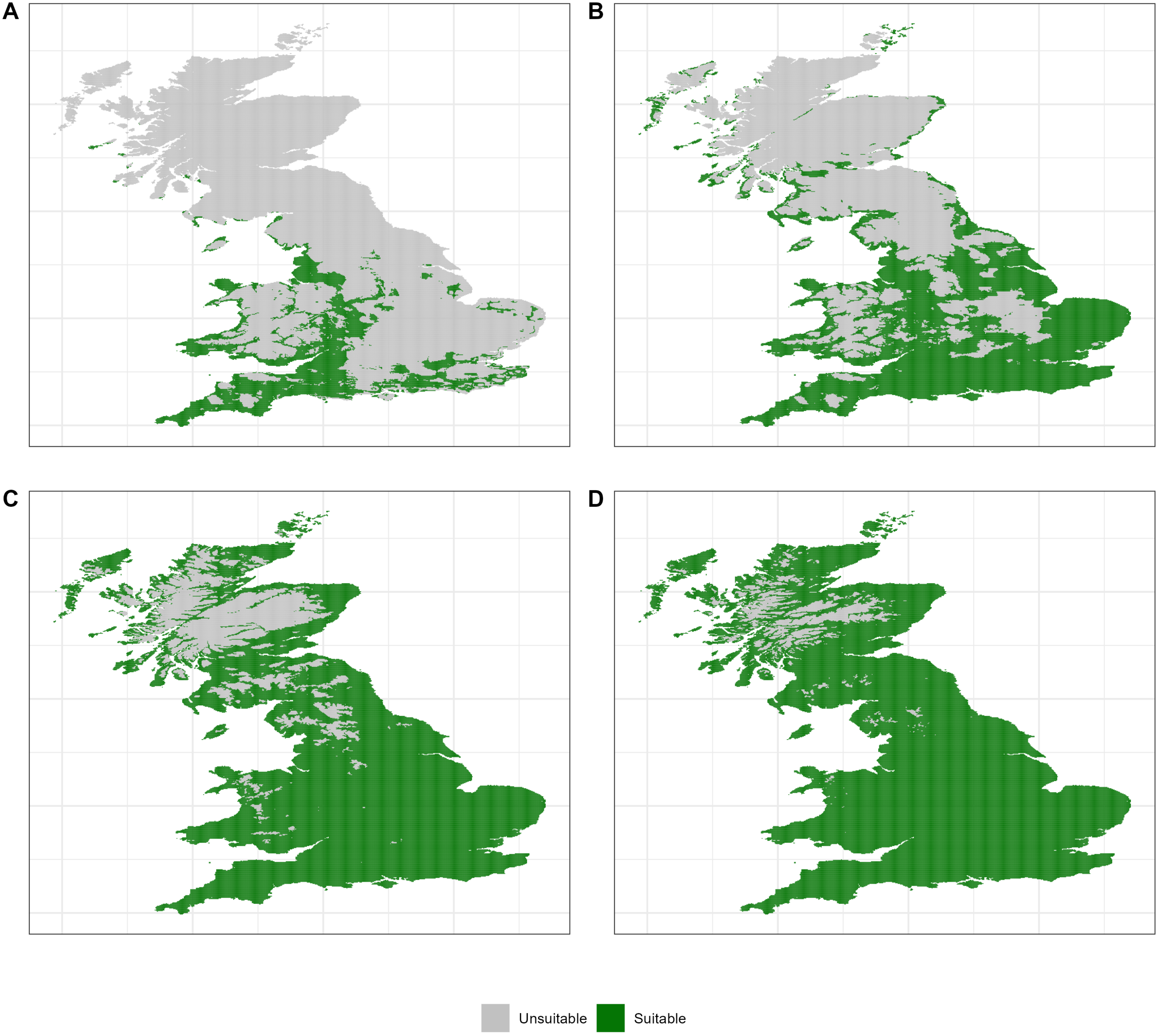

Figure S1. MaxEnt climate envelope maps for *Andrena denticulata*. Showing climate envelope for 1980-89 **(A)**, 2010-19 **(B)**, and 2070-79 under RCP 4.5 **(C)** and RCP 8.5 **(D)**.
10th percentile training presence cloglog threshold = 0.247

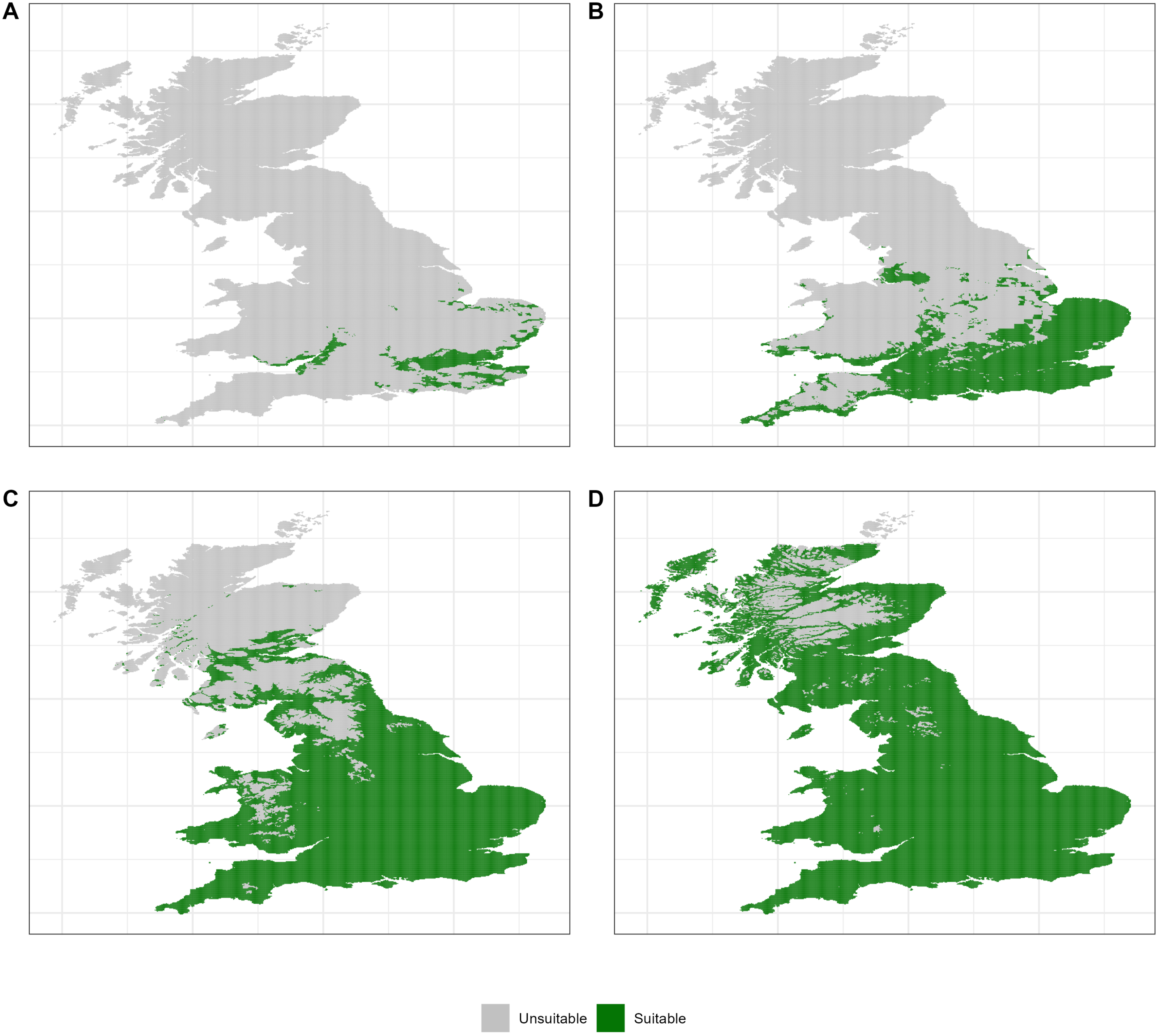

Figure S1. MaxEnt climate envelope maps for *Andrena dorsata*. Showing climate envelope for 1980-89 **(A)**, 2010-19 **(B)**, and 2070-79 under RCP 4.5 **(C)** and RCP 8.5 **(D)**.
10th percentile training presence cloglog threshold = 0.3107

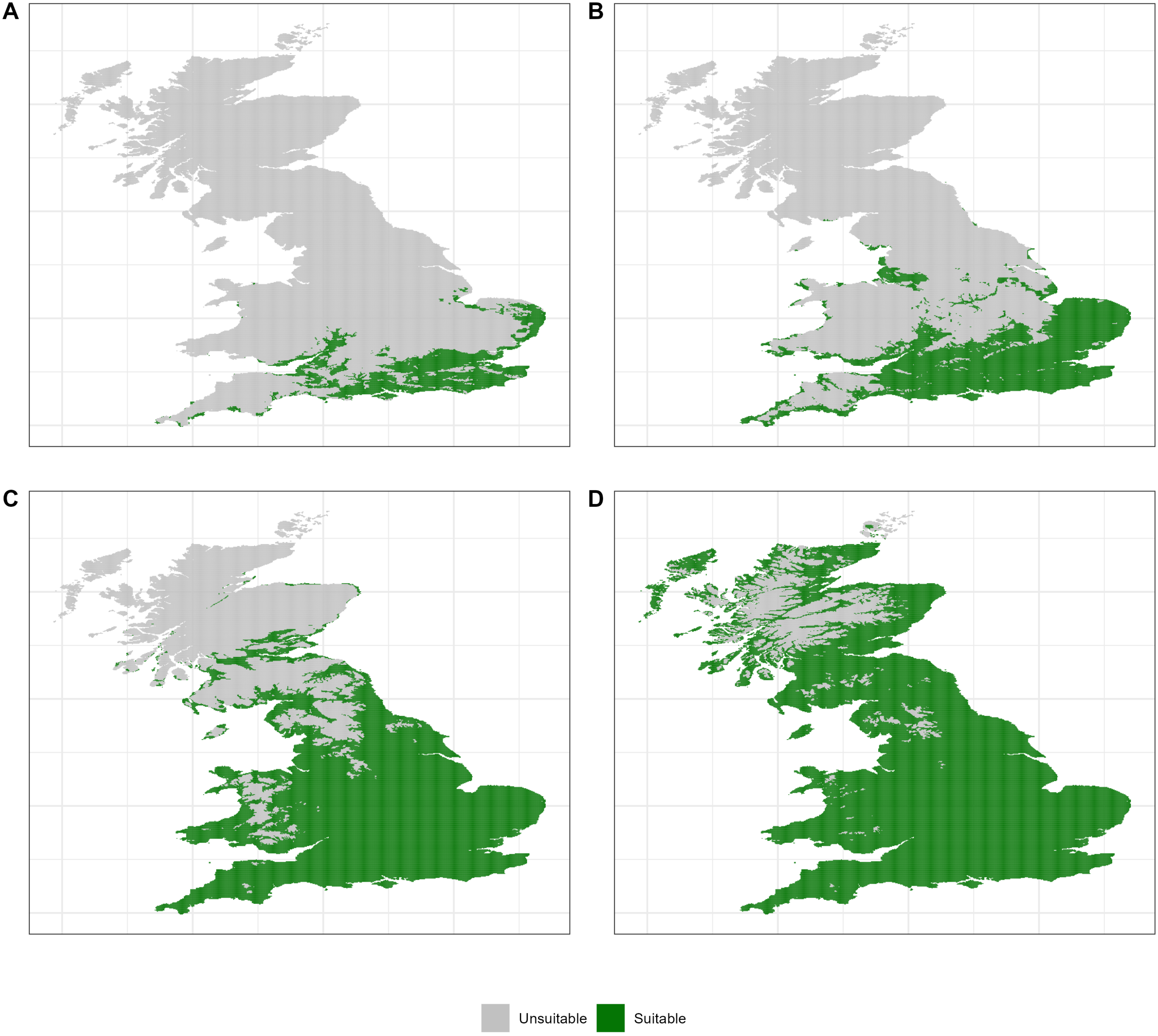

Figure S1. MaxEnt climate envelope maps for *Andrena flavipes*. Showing climate envelope for 1980-89 **(A)**, 2010-19 **(B)**, and 2070-79 under RCP 4.5 **(C)** and RCP 8.5 **(D)**.
10th percentile training presence cloglog threshold = 0.2885

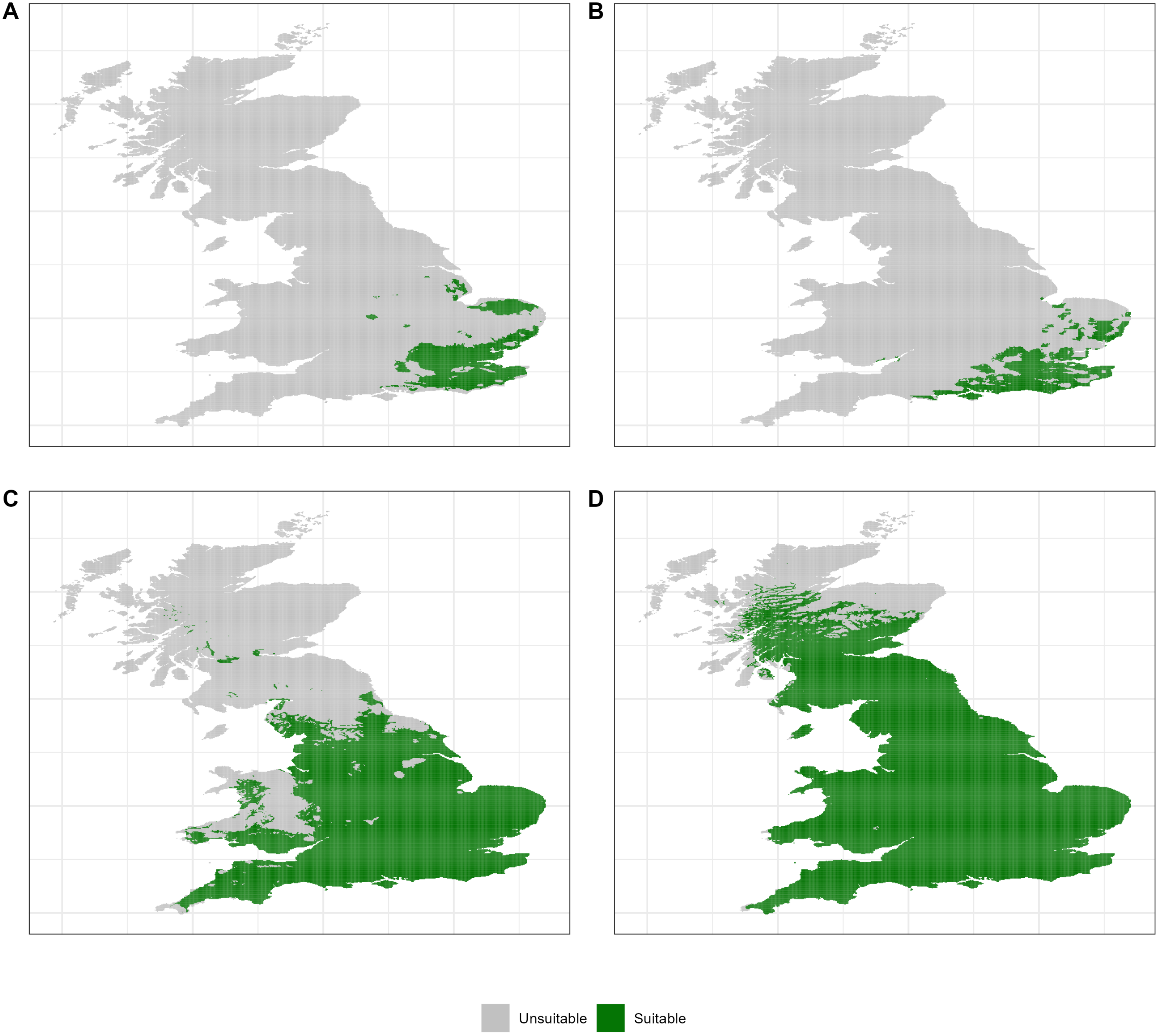

Figure S1. MaxEnt climate envelope maps for *Andrena florea*. Showing climate envelope for 1980-89 **(A)**, 2010-19 **(B)**, and 2070-79 under RCP 4.5 **(C)** and RCP 8.5 **(D)**.
10th percentile training presence cloglog threshold = 0.4457

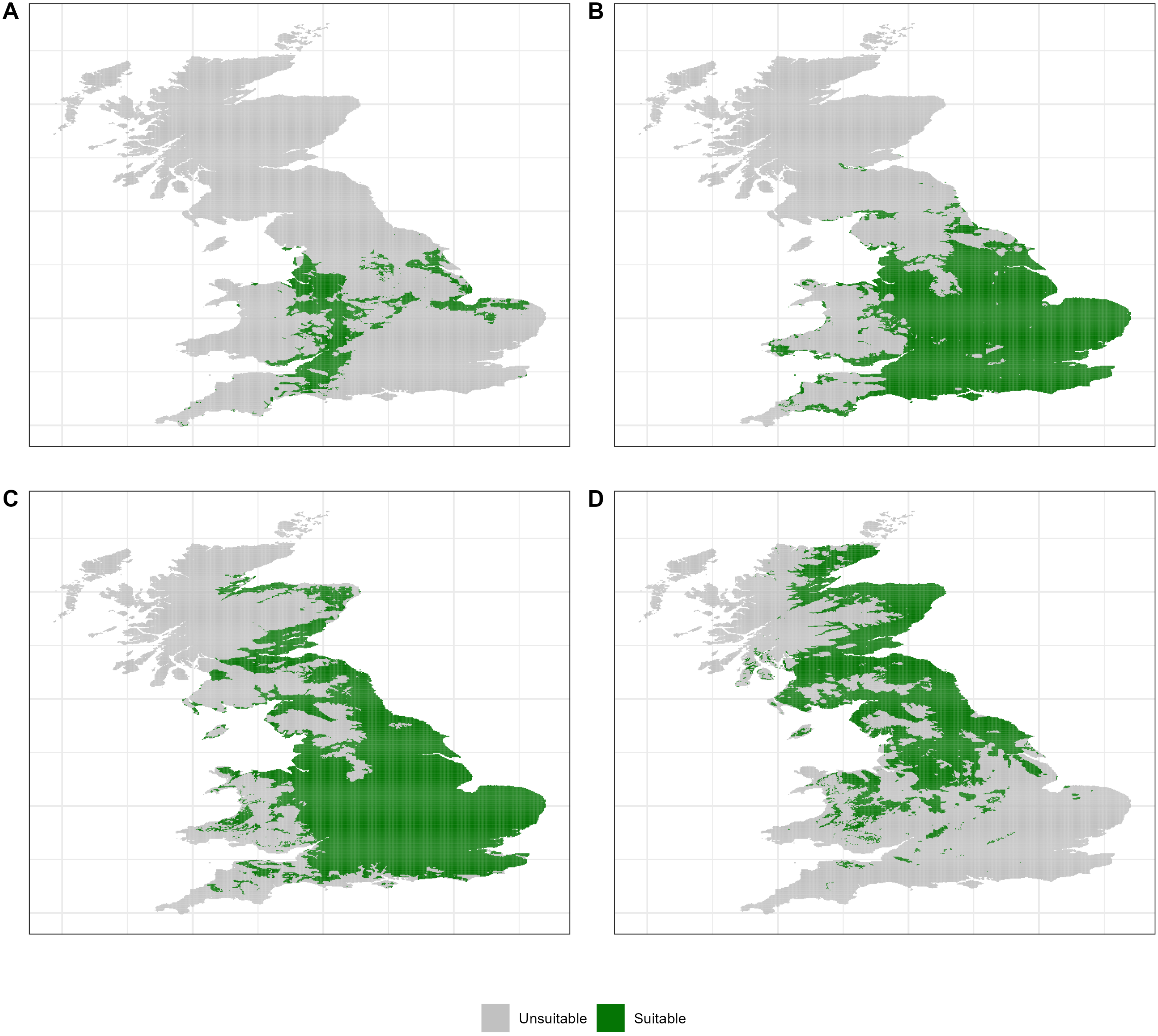

Figure S1. MaxEnt climate envelope maps for *Andrena fulva*. Showing climate envelope for 1980-89 **(A)**, 2010-19 **(B)**, and 2070-79 under RCP 4.5 **(C)** and RCP 8.5 **(D)**.
10th percentile training presence cloglog threshold = 0.3906

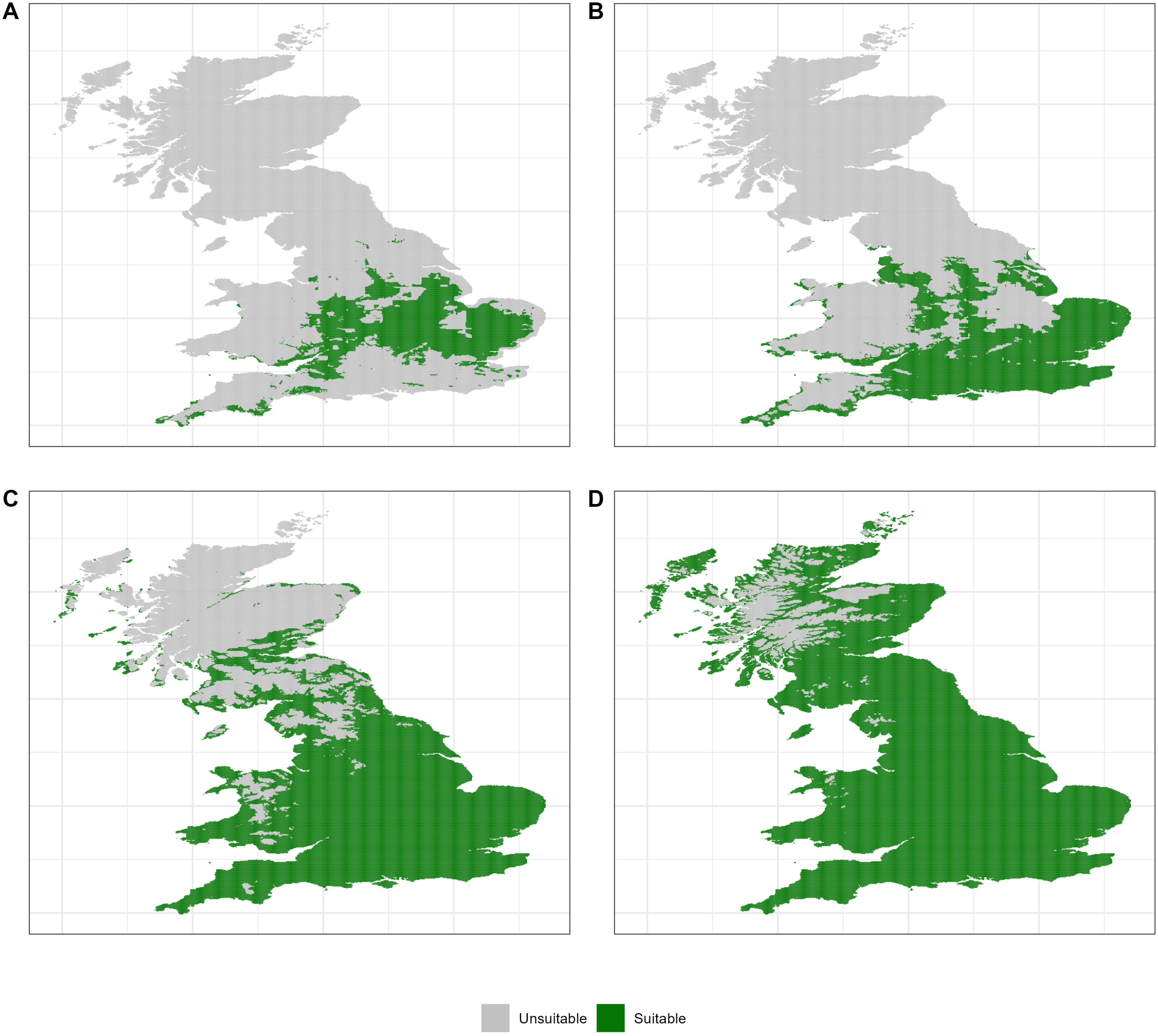

Figure S1. MaxEnt climate envelope maps for *Andrena fuscipes*. Showing climate envelope for 1980-89 **(A)**, 2010-19 **(B)**, and 2070-79 under RCP 4.5 **(C)** and RCP 8.5 **(D)**.
10th percentile training presence cloglog threshold = 0.3661

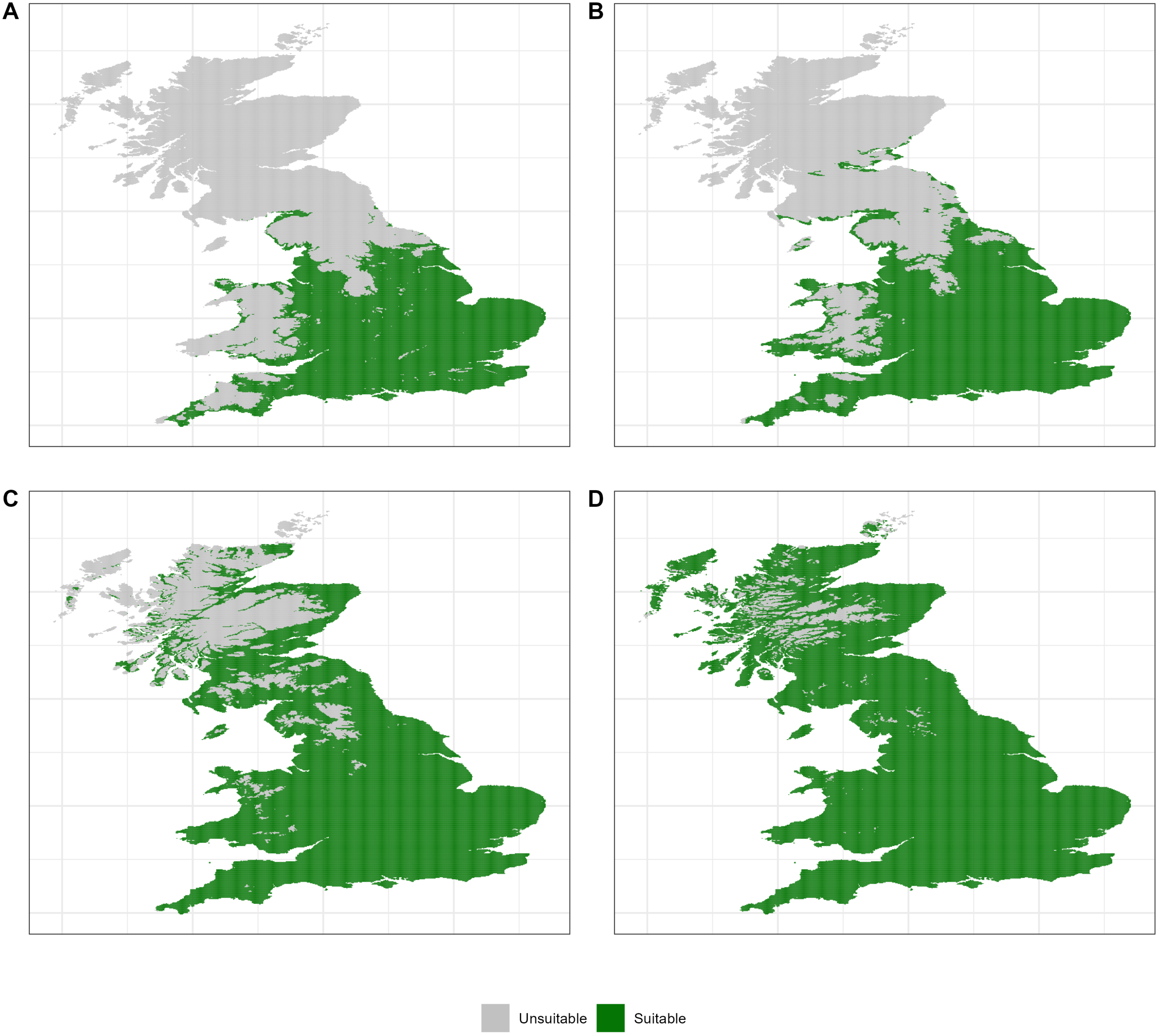

Figure S1. MaxEnt climate envelope maps for *Andrena haemorrhoa*. Showing climate envelope for 1980-89 **(A)**, 2010-19 **(B)**, and 2070-79 under RCP 4.5 **(C)** and RCP 8.5 **(D)**.
10th percentile training presence cloglog threshold = 0.3684

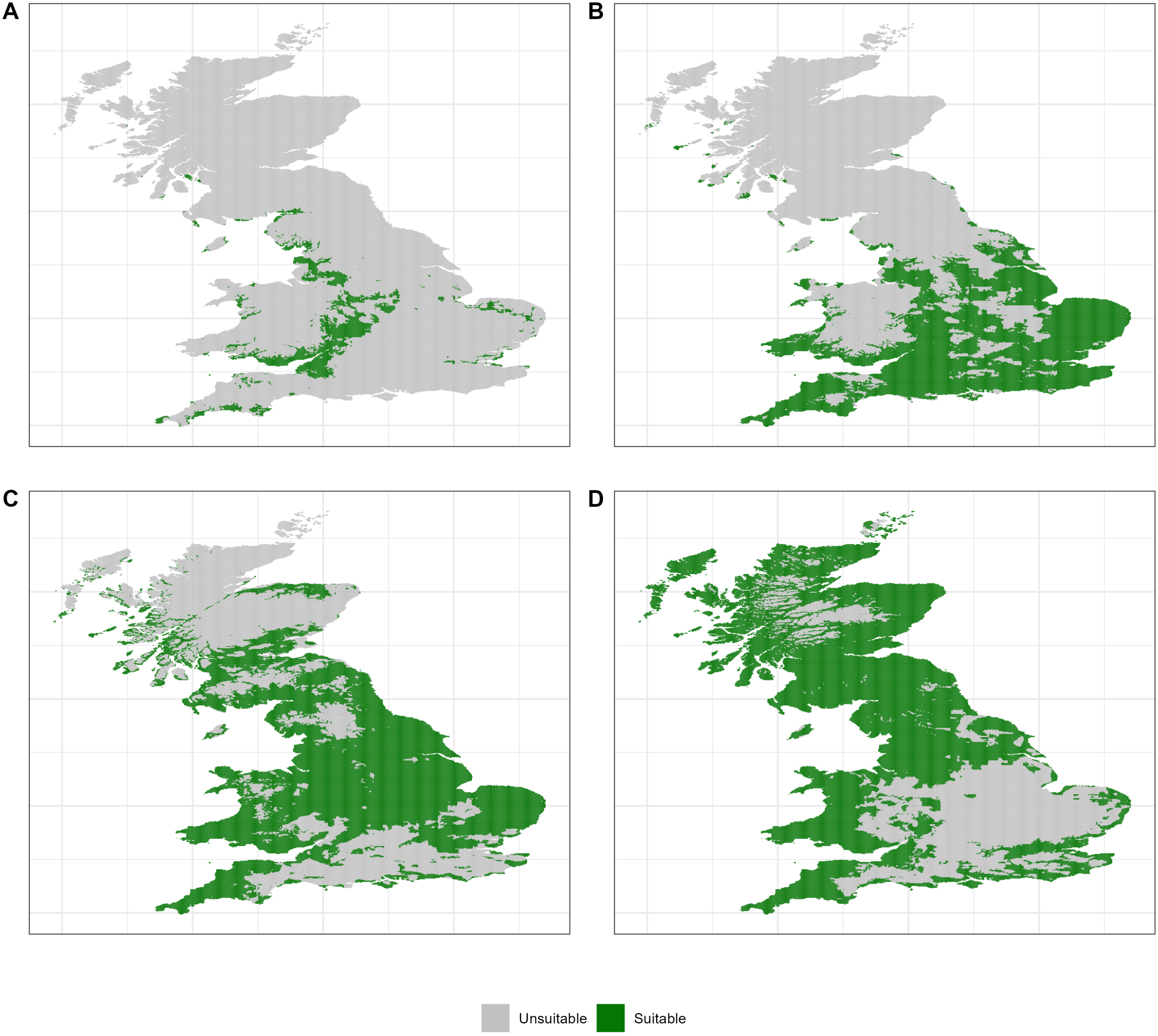

Figure S1. MaxEnt climate envelope maps for *Andrena humilis*. Showing climate envelope for 1980-89 **(A)**, 2010-19 **(B)**, and 2070-79 under RCP 4.5 **(C)** and RCP 8.5 **(D)**.
10th percentile training presence cloglog threshold = 0.2535

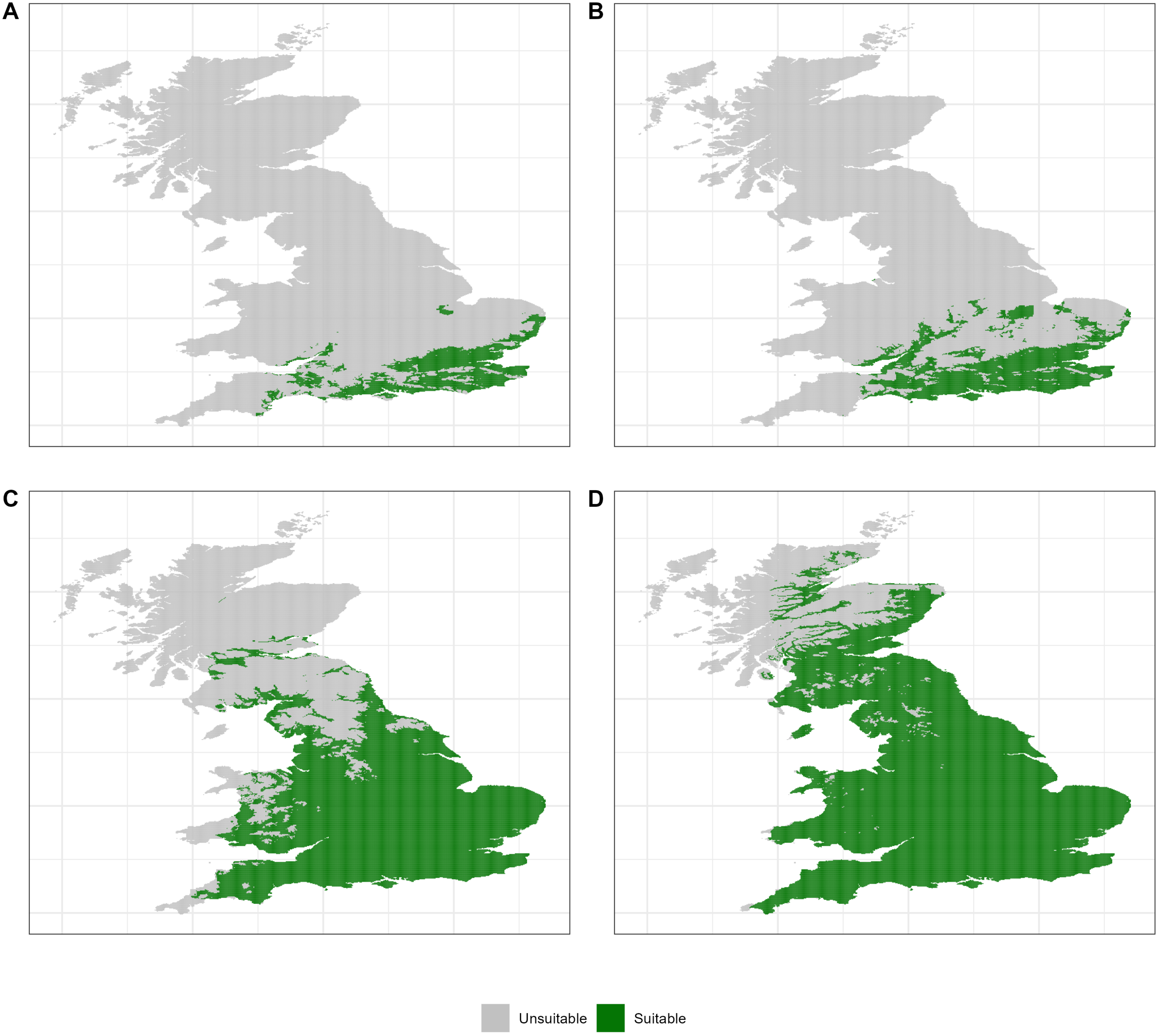

Figure S1. MaxEnt climate envelope maps for *Andrena labialis*. Showing climate envelope for 1980-89 **(A)**, 2010-19 **(B)**, and 2070-79 under RCP 4.5 **(C)** and RCP 8.5 **(D)**.
10th percentile training presence cloglog threshold = 0.297

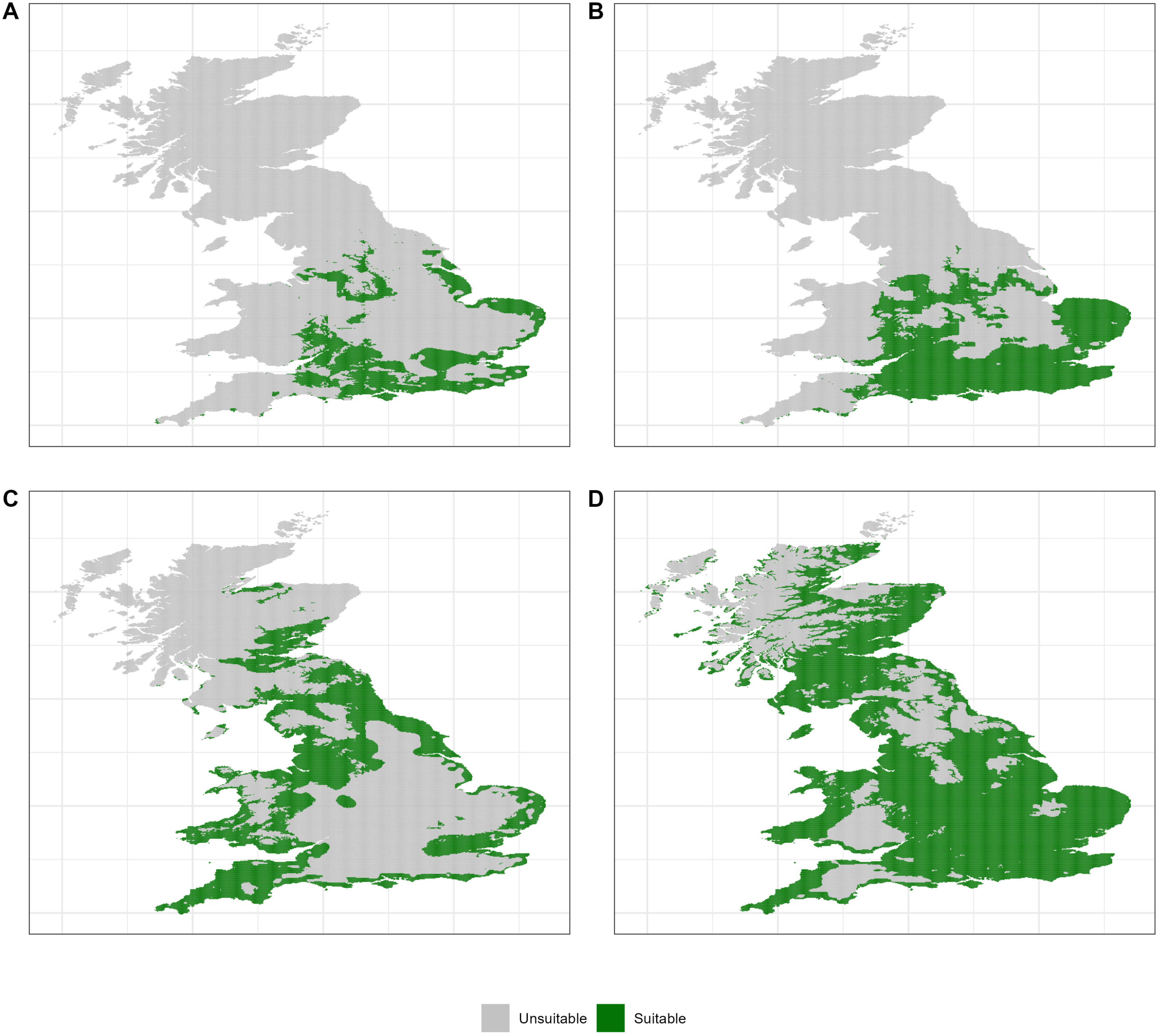

Figure S1. MaxEnt climate envelope maps for *Andrena labiata*. Showing climate envelope for 1980-89 **(A)**, 2010-19 **(B)**, and 2070-79 under RCP 4.5 **(C)** and RCP 8.5 **(D)**.
10th percentile training presence cloglog threshold = 0.2949

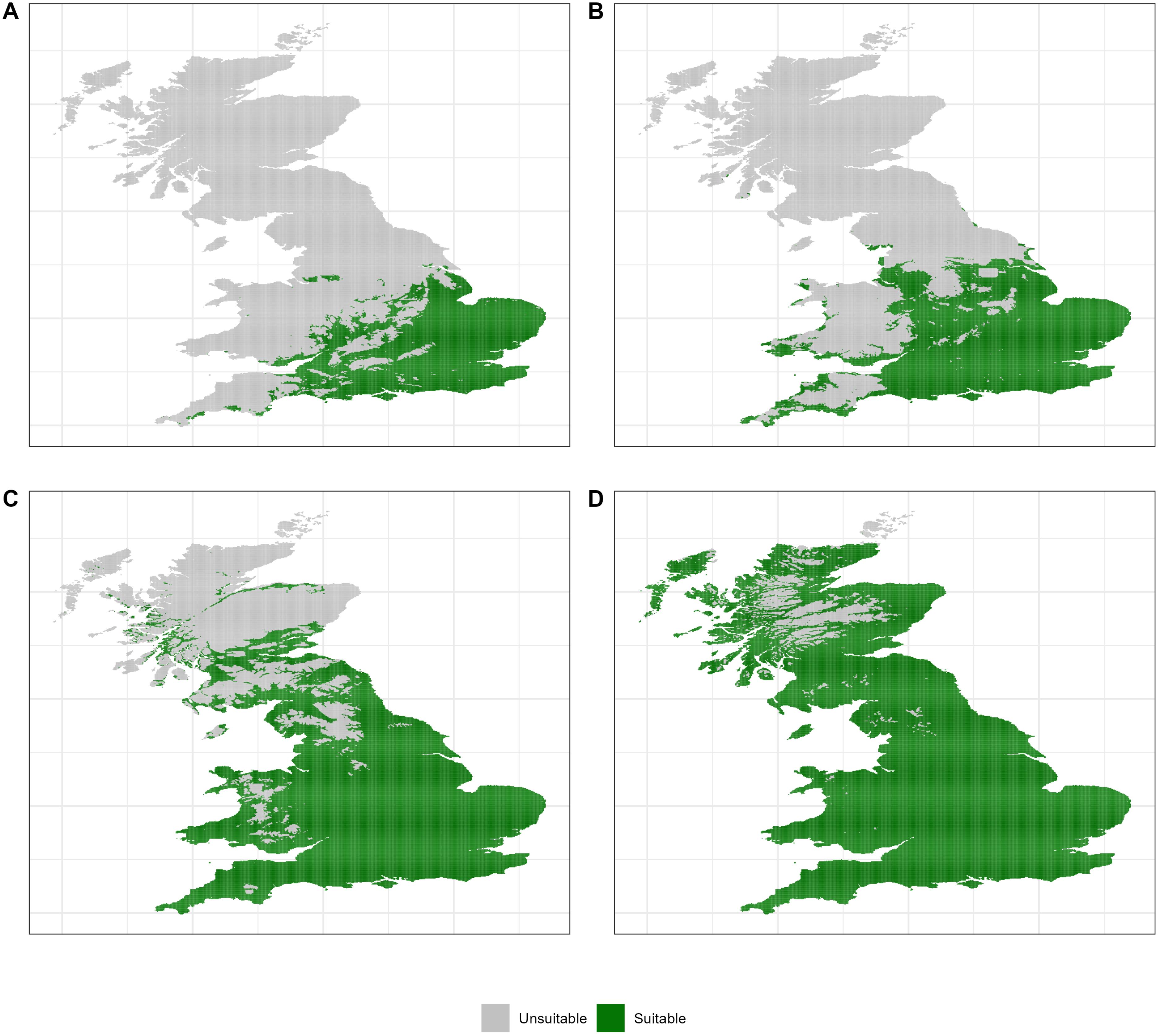

Figure S1. MaxEnt climate envelope maps for *Andrena minutula*. Showing climate envelope for 1980-89 **(A)**, 2010-19 **(B)**, and 2070-79 under RCP 4.5 **(C)** and RCP 8.5 **(D)**.
10th percentile training presence cloglog threshold = 0.3404

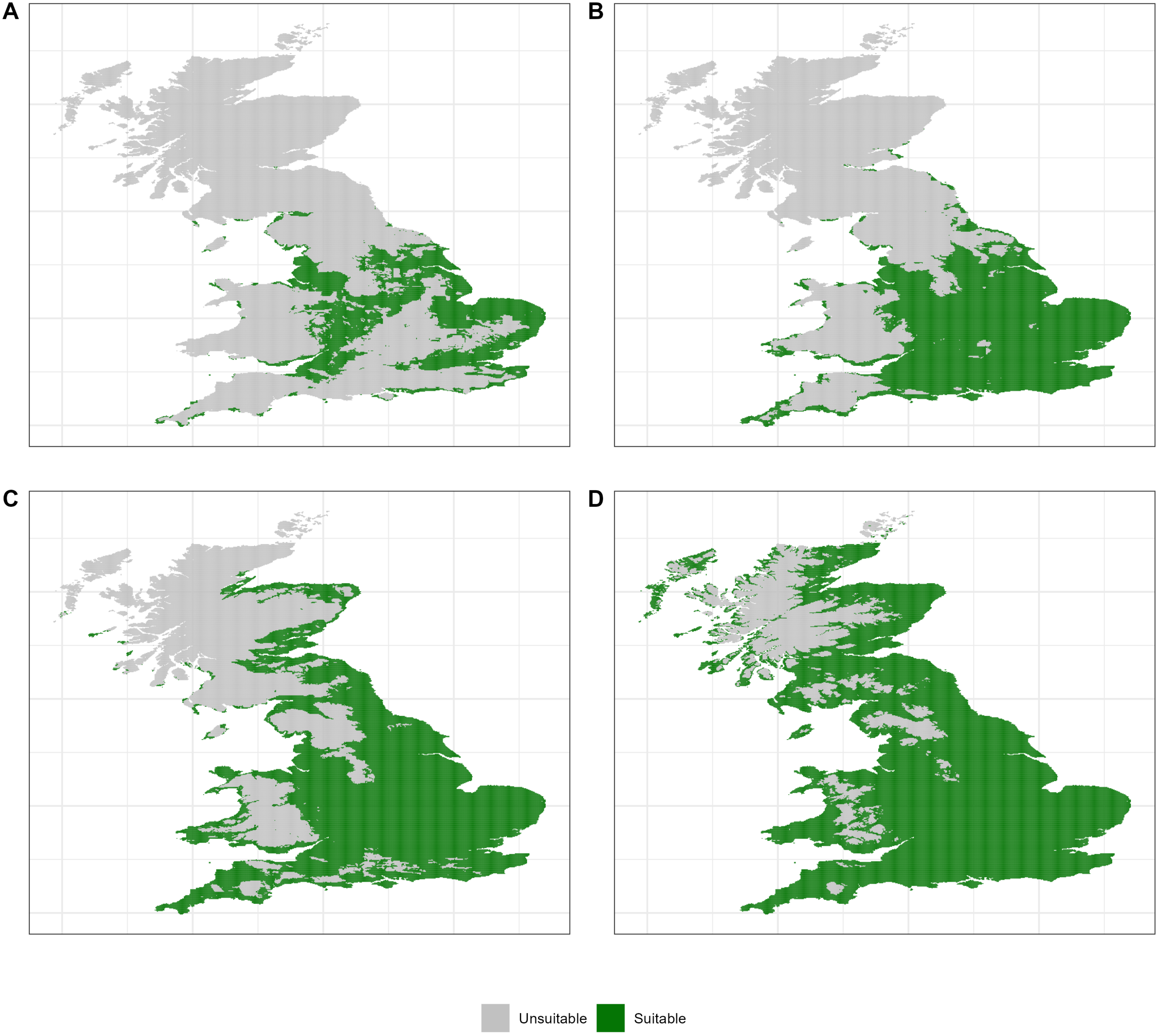

Figure S1. MaxEnt climate envelope maps for *Andrena nigroaenea*. Showing climate envelope for 1980-89 **(A)**, 2010-19 **(B)**, and 2070-79 under RCP 4.5 **(C)** and RCP 8.5 **(D)**.
10th percentile training presence cloglog threshold = 0.3494

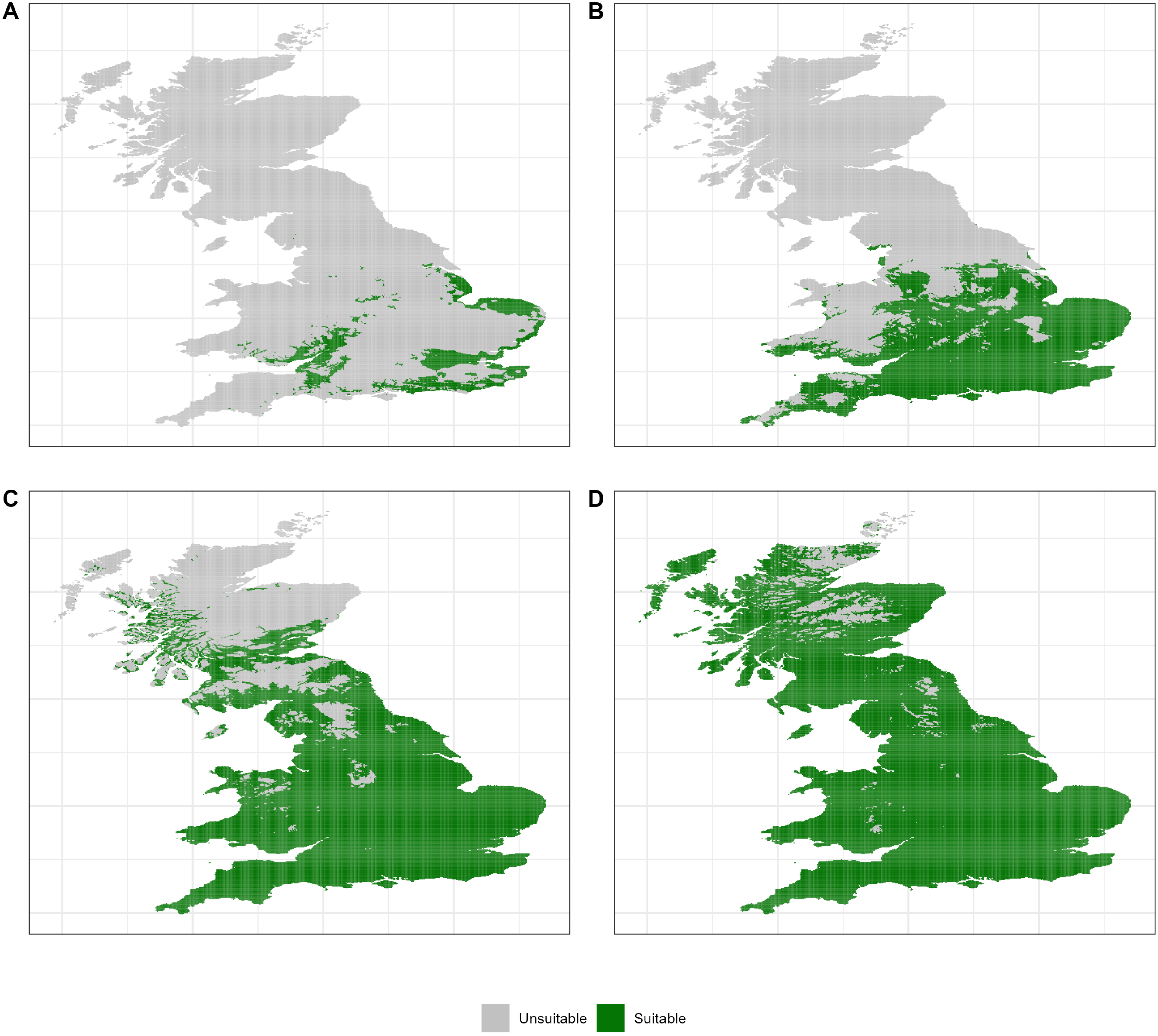

Figure S1. MaxEnt climate envelope maps for *Andrena nitida*. Showing climate envelope for 1980-89 **(A)**, 2010-19 **(B)**, and 2070-79 under RCP 4.5 **(C)** and RCP 8.5 **(D)**.
10th percentile training presence cloglog threshold = 0.3388

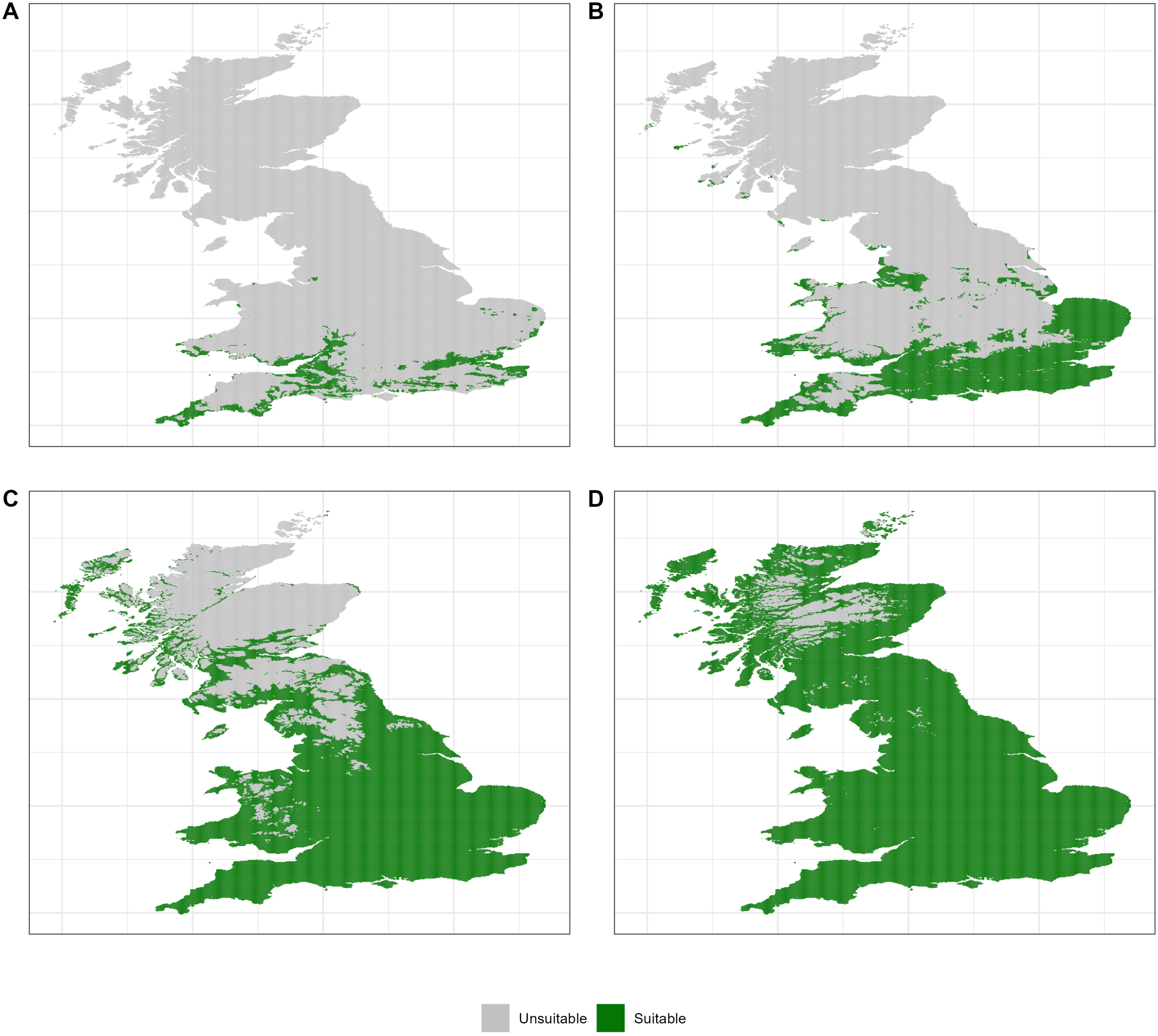

Figure S1. MaxEnt climate envelope maps for *Andrena ovatula*. Showing climate envelope for 1980-89 **(A)**, 2010-19 **(B)**, and 2070-79 under RCP 4.5 **(C)** and RCP 8.5 **(D)**.
10th percentile training presence cloglog threshold = 0.2786

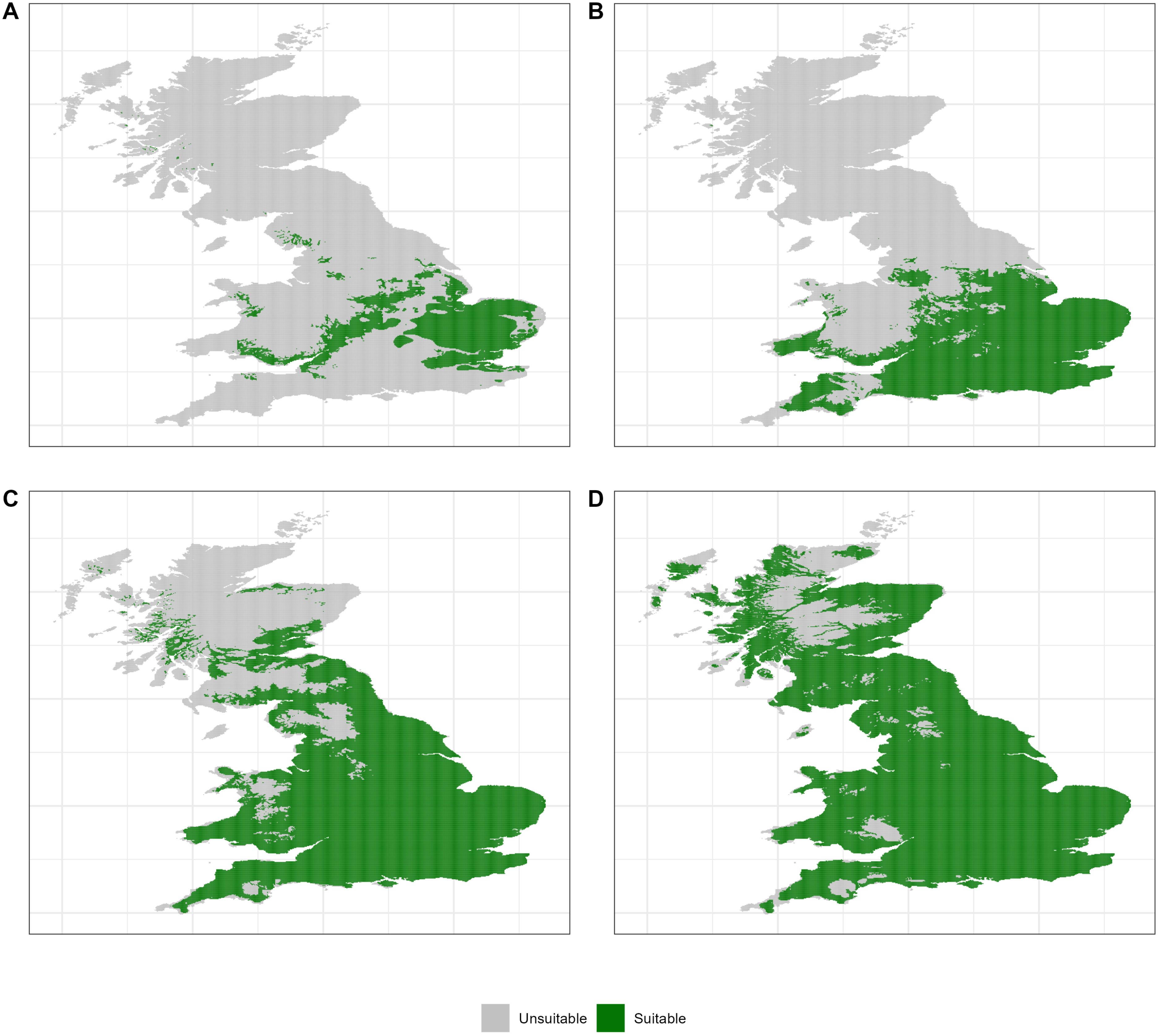

Figure S1. MaxEnt climate envelope maps for *Andrena praecox*. Showing climate envelope for 1980-89 **(A)**, 2010-19 **(B)**, and 2070-79 under RCP 4.5 **(C)** and RCP 8.5 **(D)**.
10th percentile training presence cloglog threshold = 0.3198

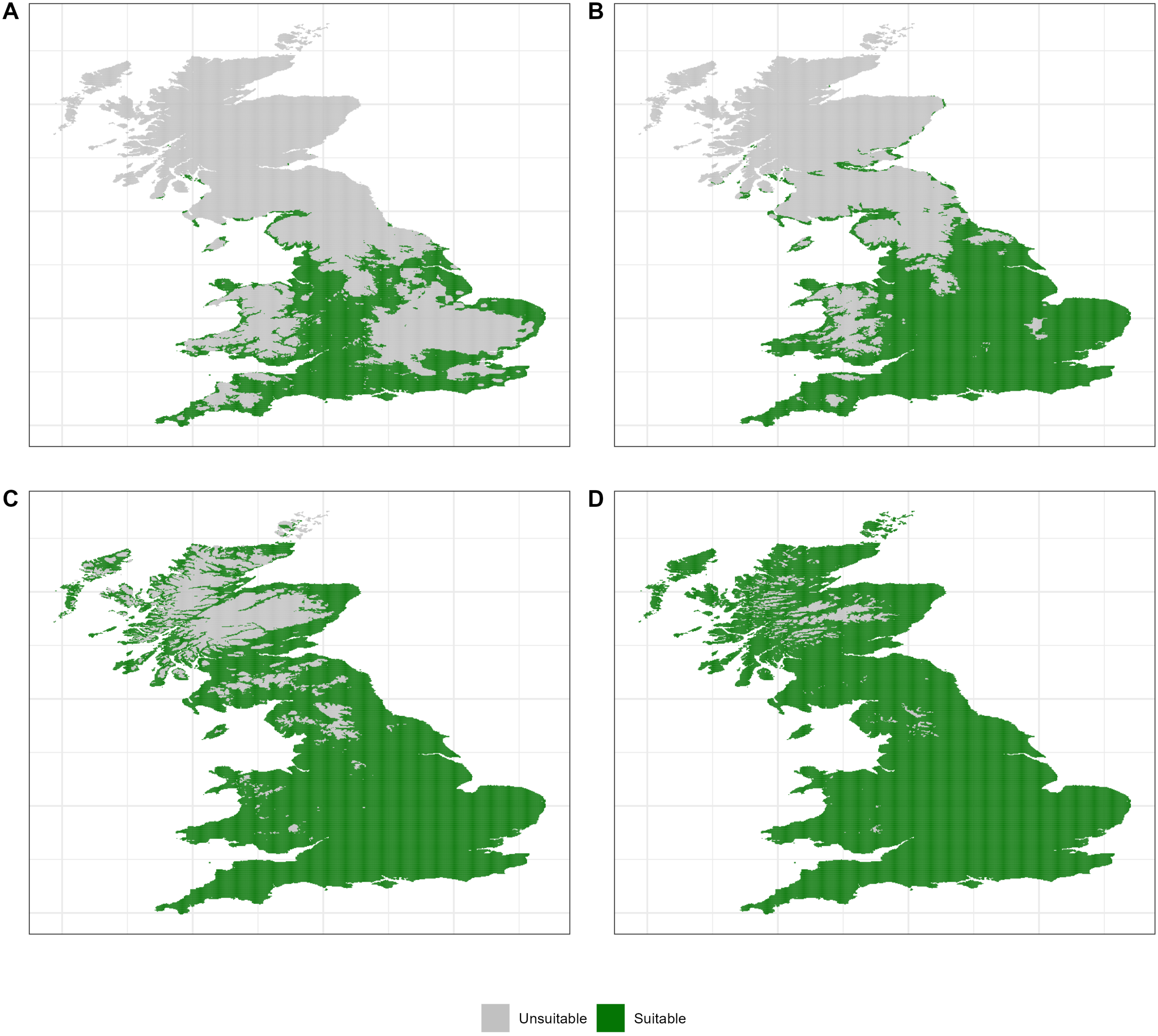

Figure S1. MaxEnt climate envelope maps for *Andrena scotica*. Showing climate envelope for 1980-89 **(A)**, 2010-19 **(B)**, and 2070-79 under RCP 4.5 **(C)** and RCP 8.5 **(D)**.
10th percentile training presence cloglog threshold = 0.3597

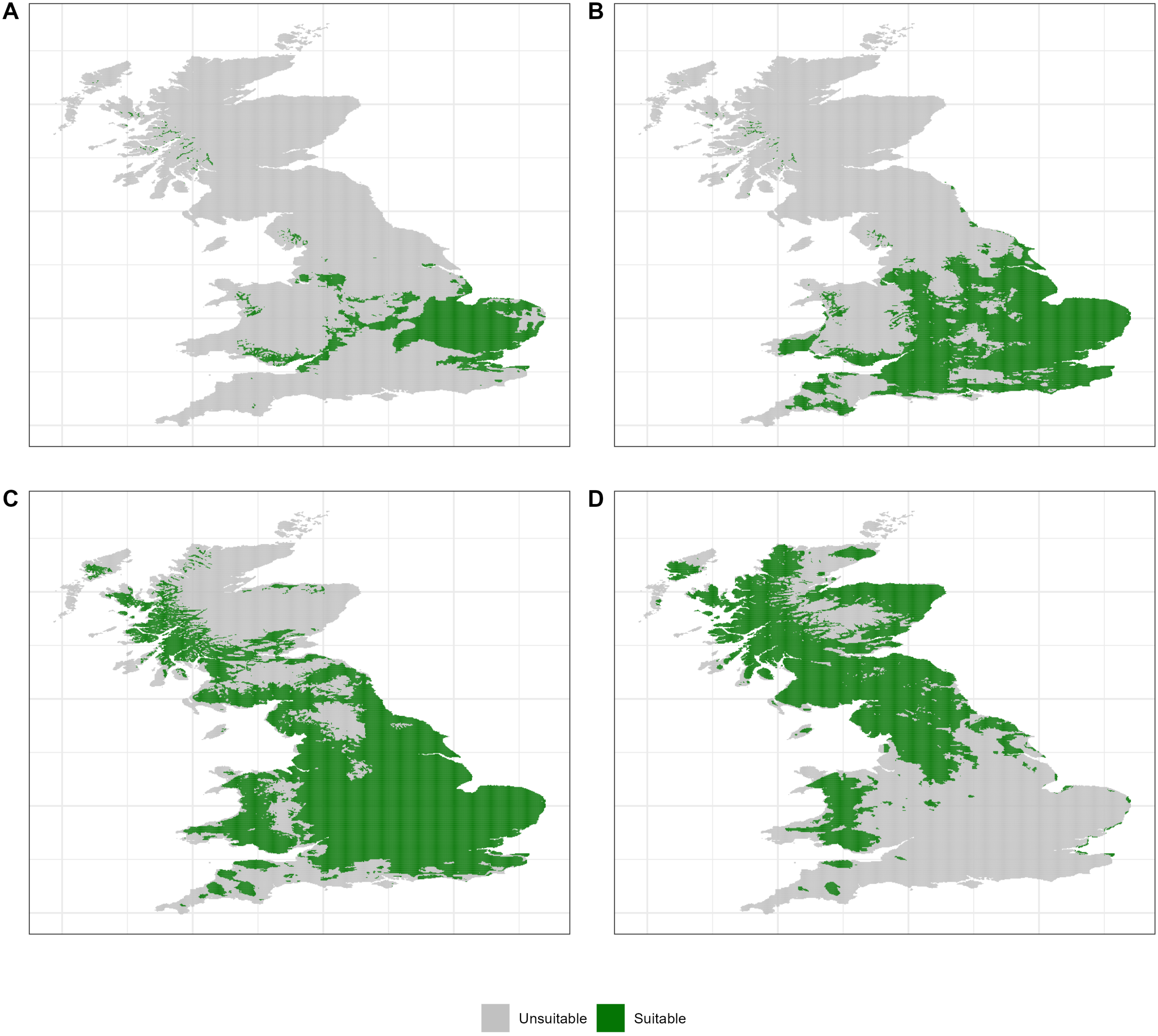

Figure S1. MaxEnt climate envelope maps for *Andrena synadelpha*. Showing climate envelope for 1980-89 **(A)**, 2010-19 **(B)**, and 2070-79 under RCP 4.5 **(C)** and RCP 8.5 **(D)**.
10th percentile training presence cloglog threshold = 0.3099

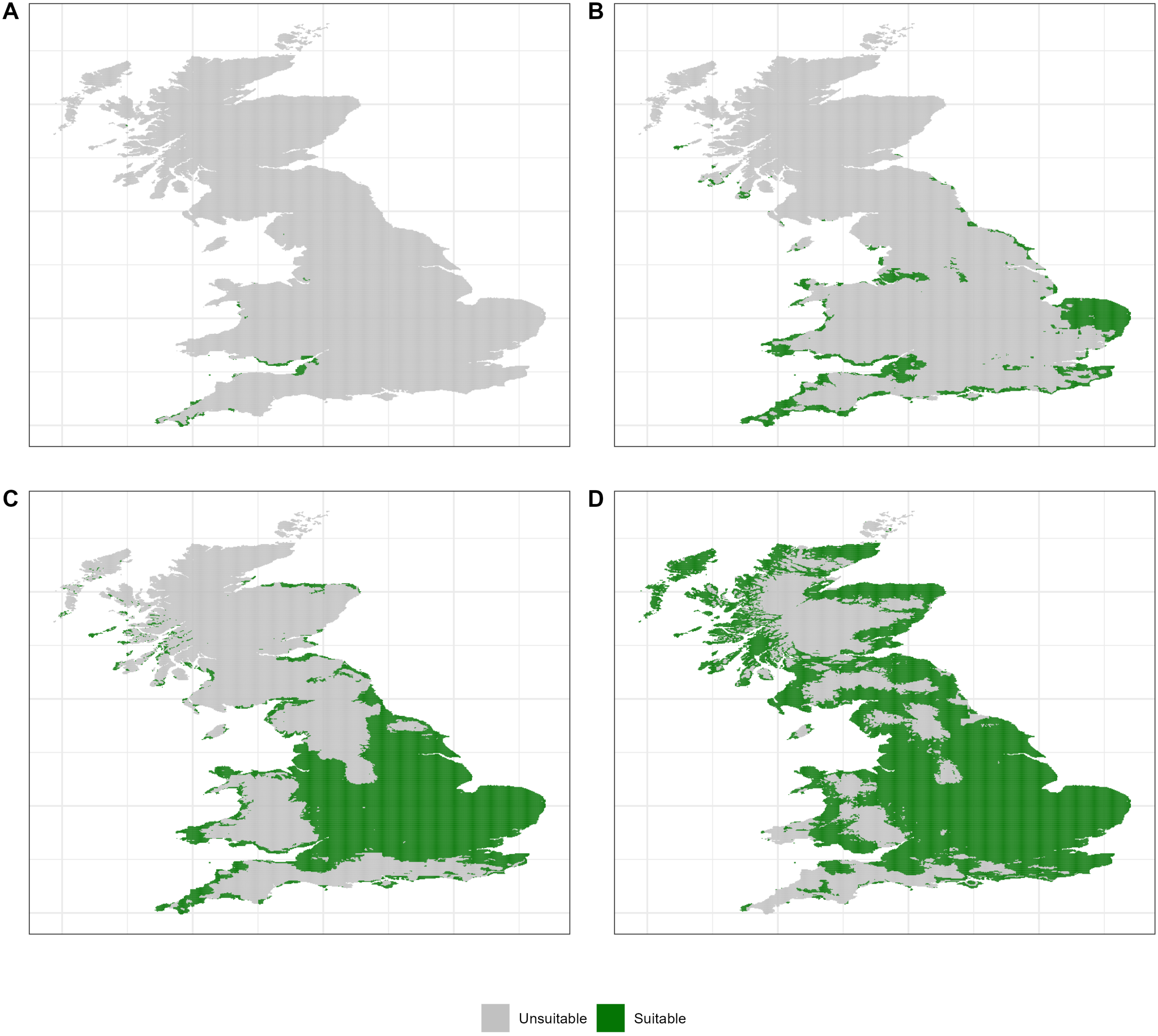

Figure S1. MaxEnt climate envelope maps for *Andrena thoracica*. Showing climate envelope for 1980-89 **(A)**, 2010-19 **(B)**, and 2070-79 under RCP 4.5 **(C)** and RCP 8.5 **(D)**.
10th percentile training presence cloglog threshold = 0.2442

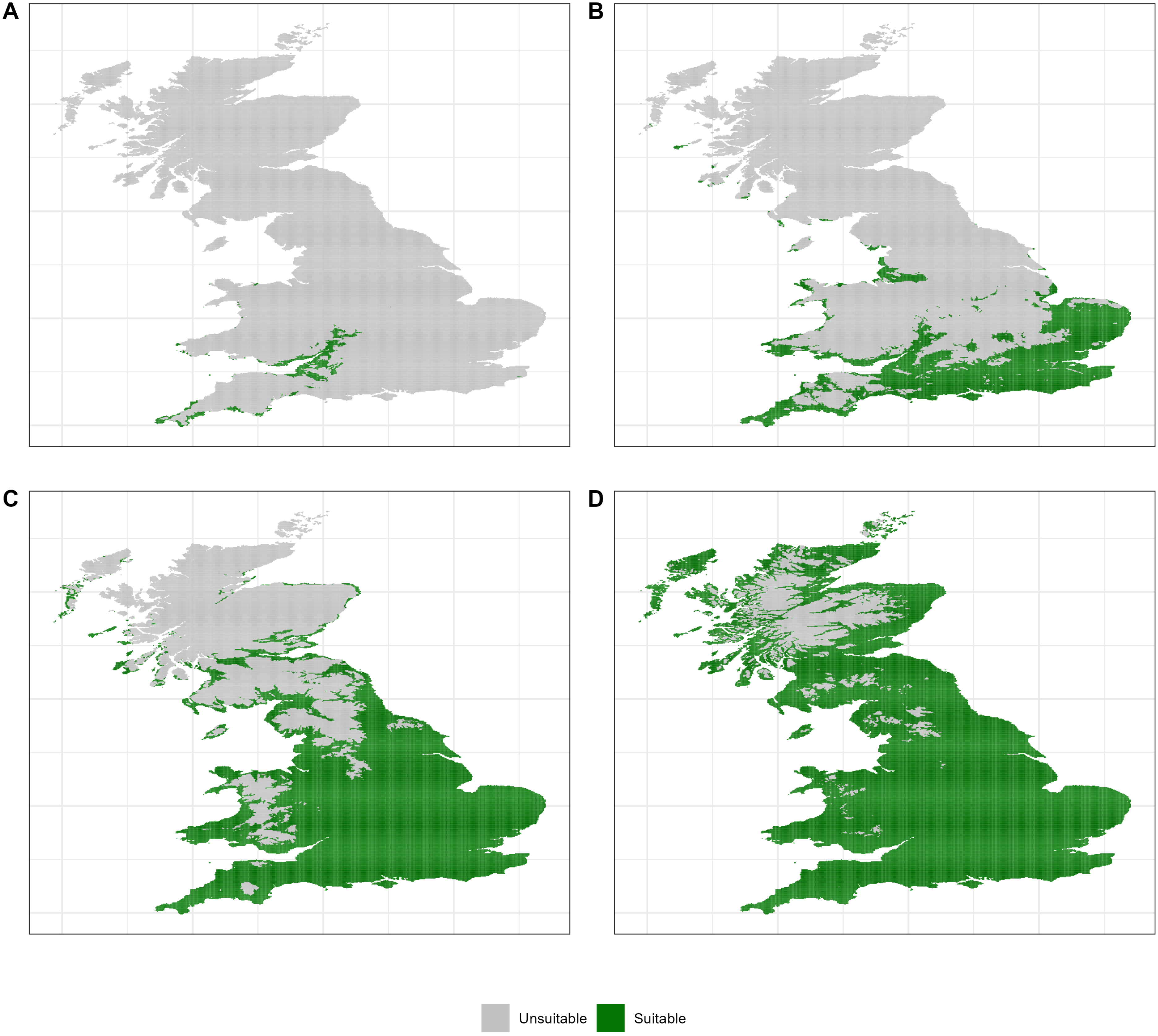

Figure S1. MaxEnt climate envelope maps for *Andrena trimmerana*. Showing climate envelope for 1980-89 **(A)**, 2010-19 **(B)**, and 2070-79 under RCP 4.5 **(C)** and RCP 8.5 **(D)**.
10th percentile training presence cloglog threshold = 0.1963

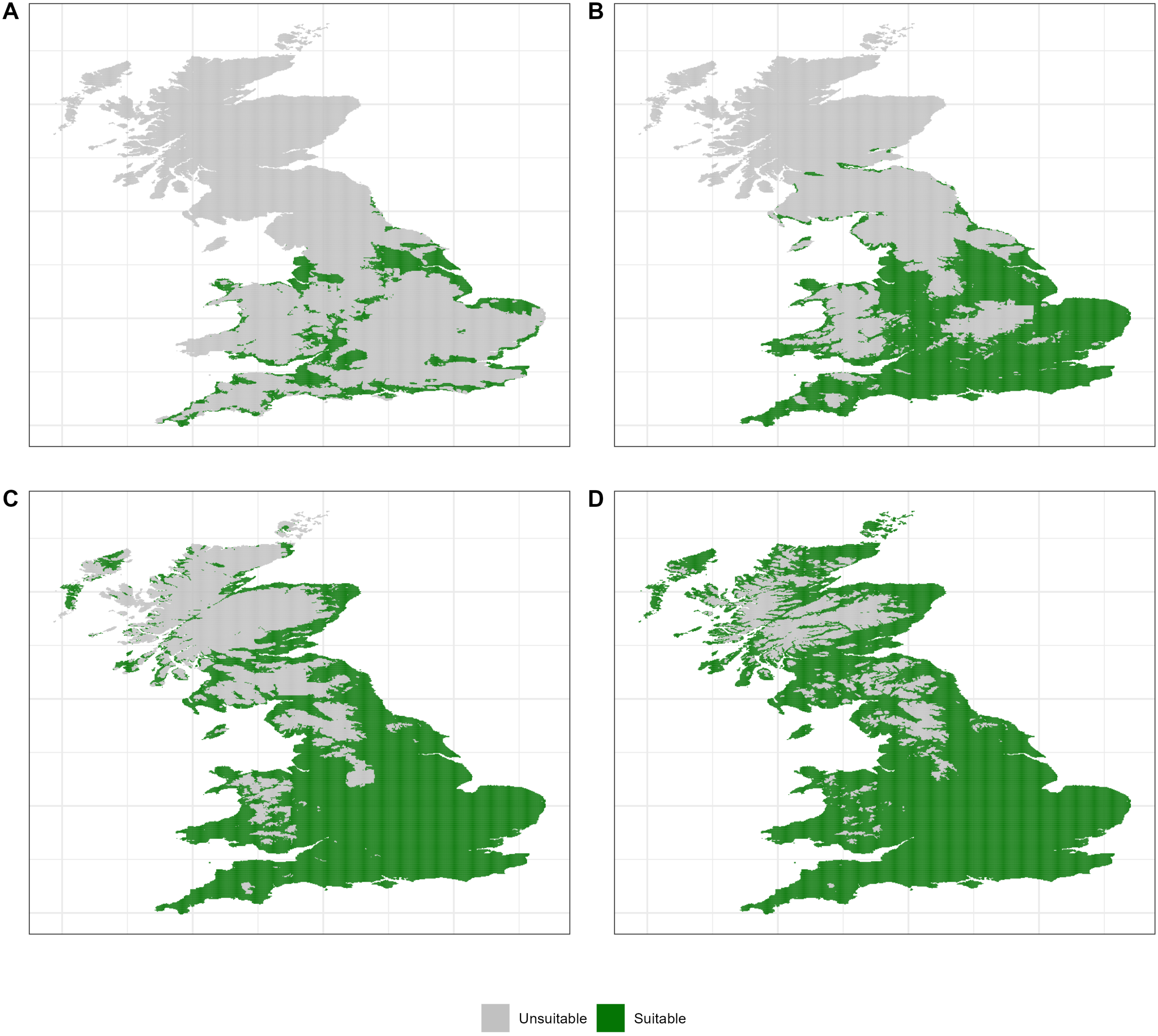

Figure S1. MaxEnt climate envelope maps for *Anthidium manicatum*. Showing climate envelope for 1980-89 **(A)**, 2010-19 **(B)**, and 2070-79 under RCP 4.5 **(C)** and RCP 8.5 **(D)**.
10th percentile training presence cloglog threshold = 0.2756

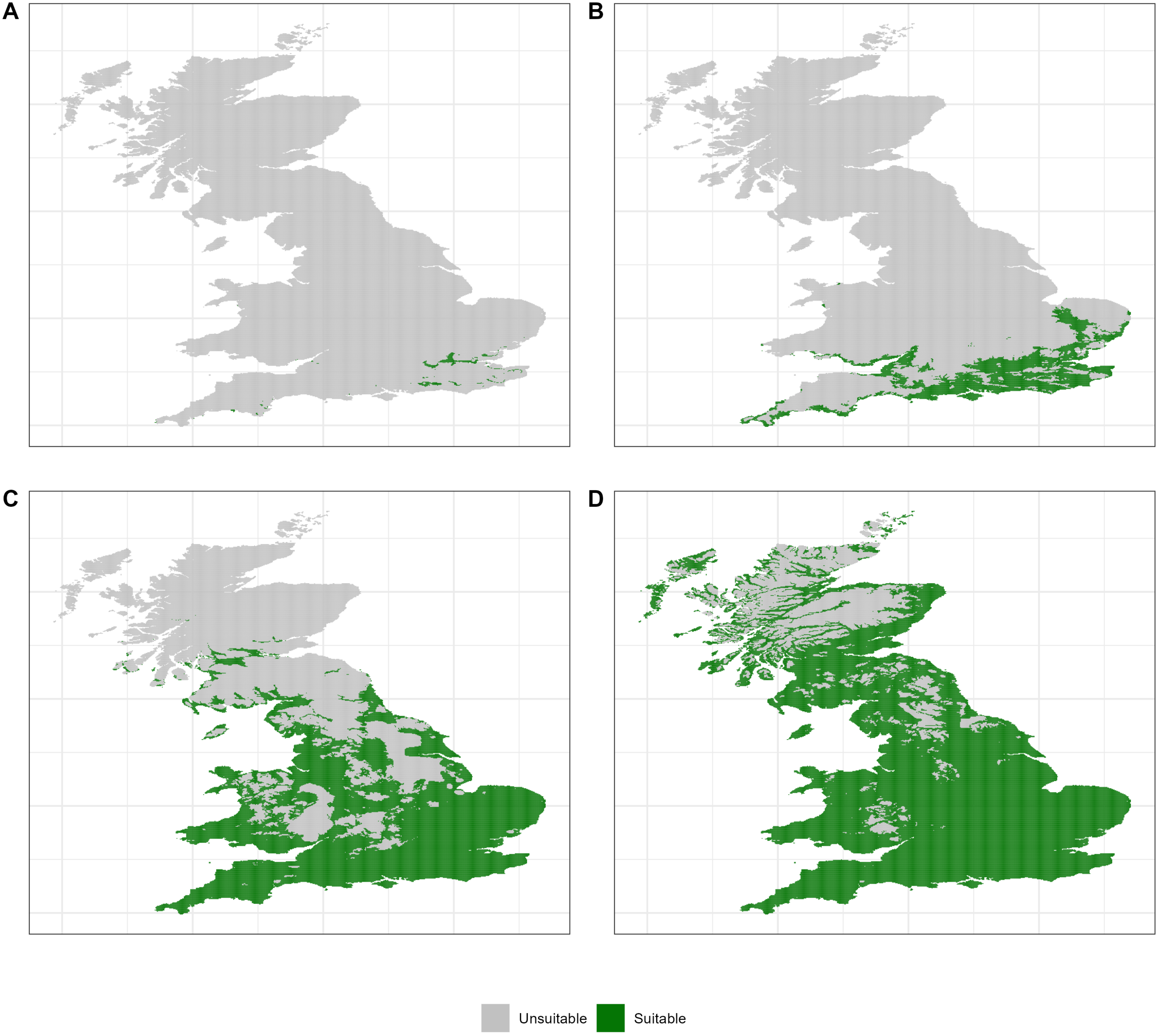

Figure S1. MaxEnt climate envelope maps for *Anthophora bimaculata*. Showing climate envelope for 1980-89 **(A)**, 2010-19 **(B)**, and 2070-79 under RCP 4.5 **(C)** and RCP 8.5 **(D)**.
10th percentile training presence cloglog threshold = 0.3039

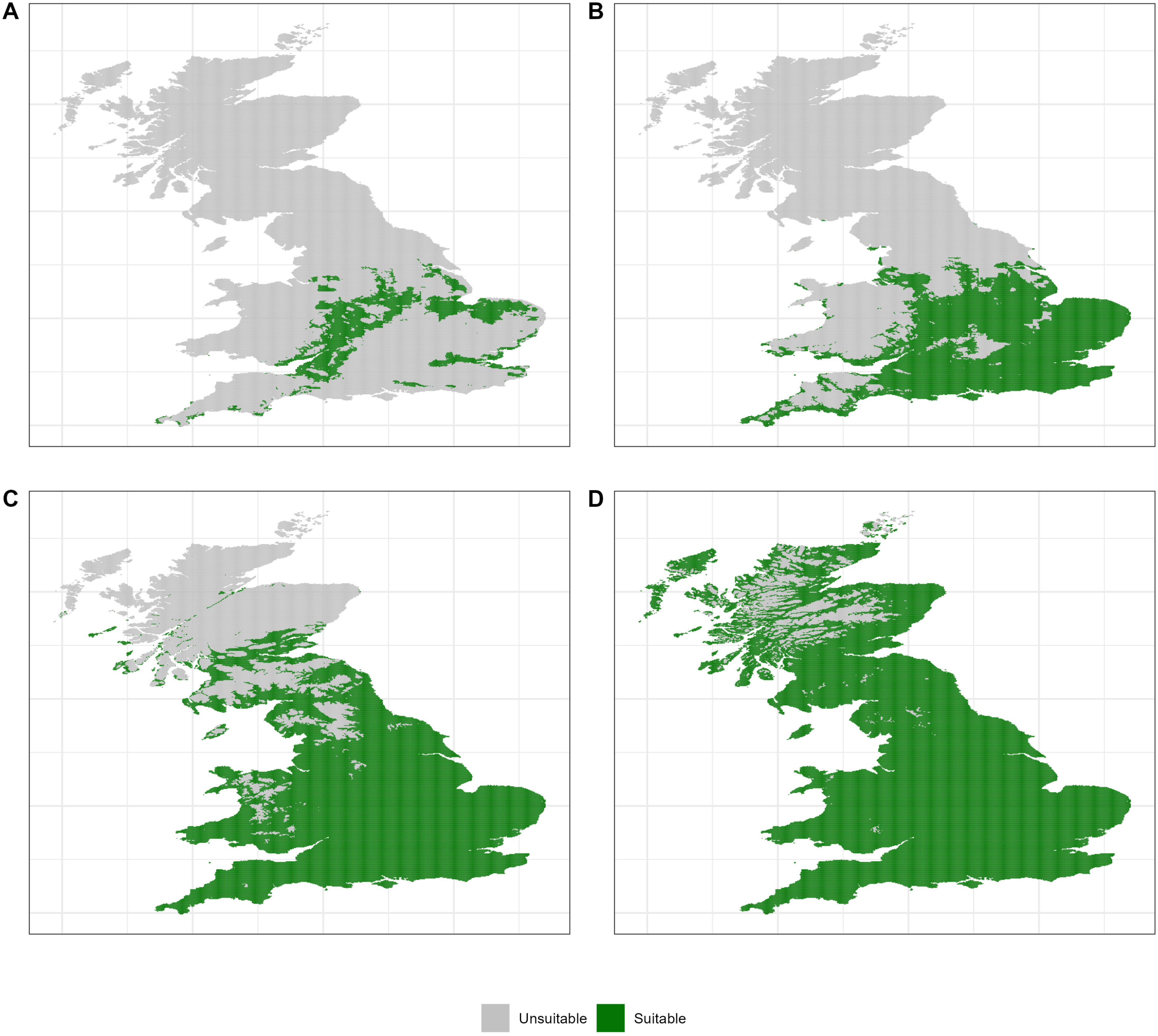

Figure S1. MaxEnt climate envelope maps for *Anthophora plumipes*. Showing climate envelope for 1980-89 **(A)**, 2010-19 **(B)**, and 2070-79 under RCP 4.5 **(C)** and RCP 8.5 **(D)**.
10th percentile training presence cloglog threshold = 0.2951

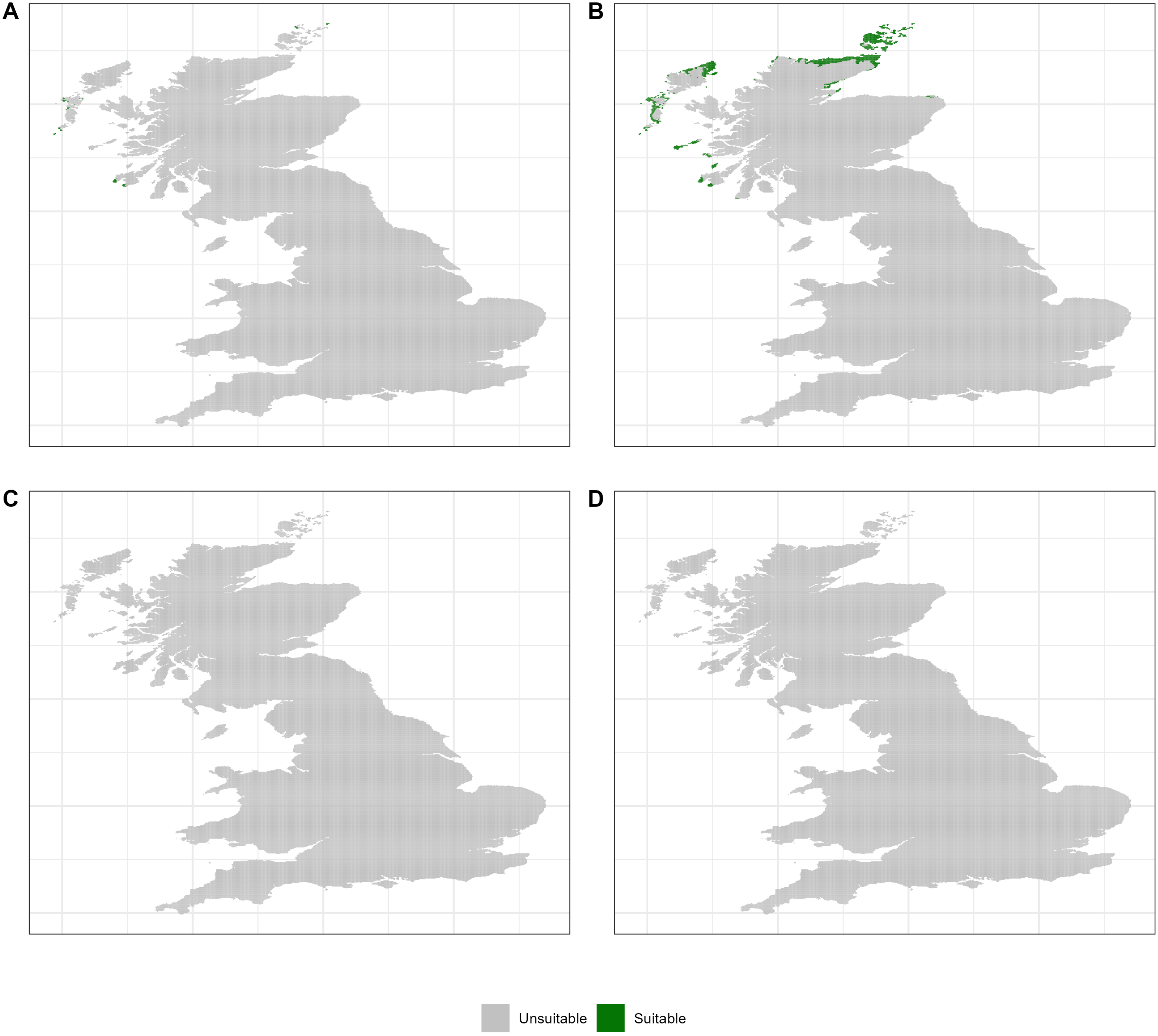

Figure S1. MaxEnt climate envelope maps for *Bombus distinguendus*. Showing climate envelope for 1980-89 **(A)**, 2010-19 **(B)**, and 2070-79 under RCP 4.5 **(C)** and RCP 8.5 **(D)**.
10th percentile training presence cloglog threshold = 0.3454

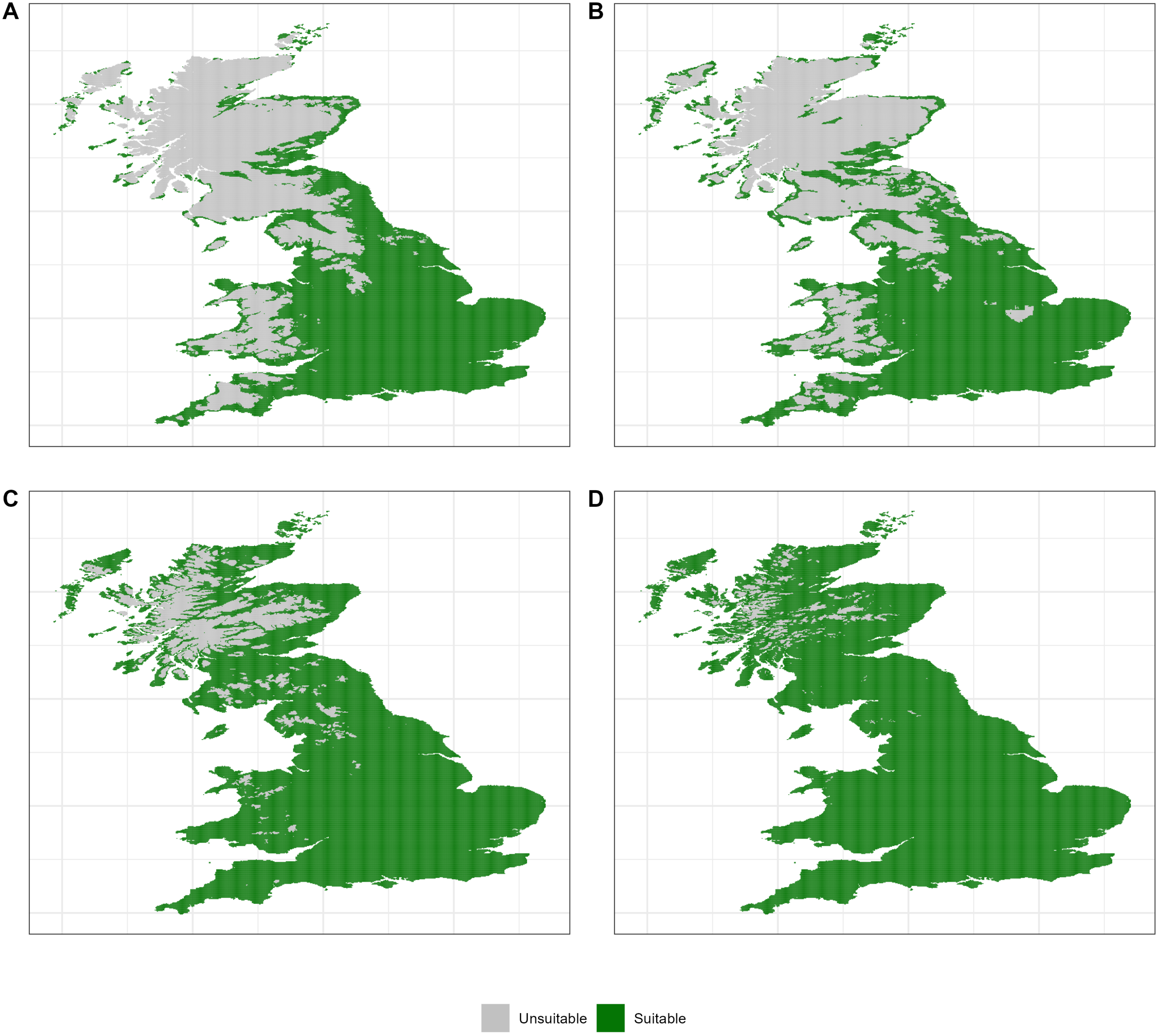

Figure S1. MaxEnt climate envelope maps for *Bombus hortorum*. Showing climate envelope for 1980-89 **(A)**, 2010-19 **(B)**, and 2070-79 under RCP 4.5 **(C)** and RCP 8.5 **(D)**.
10th percentile training presence cloglog threshold = 0.3167

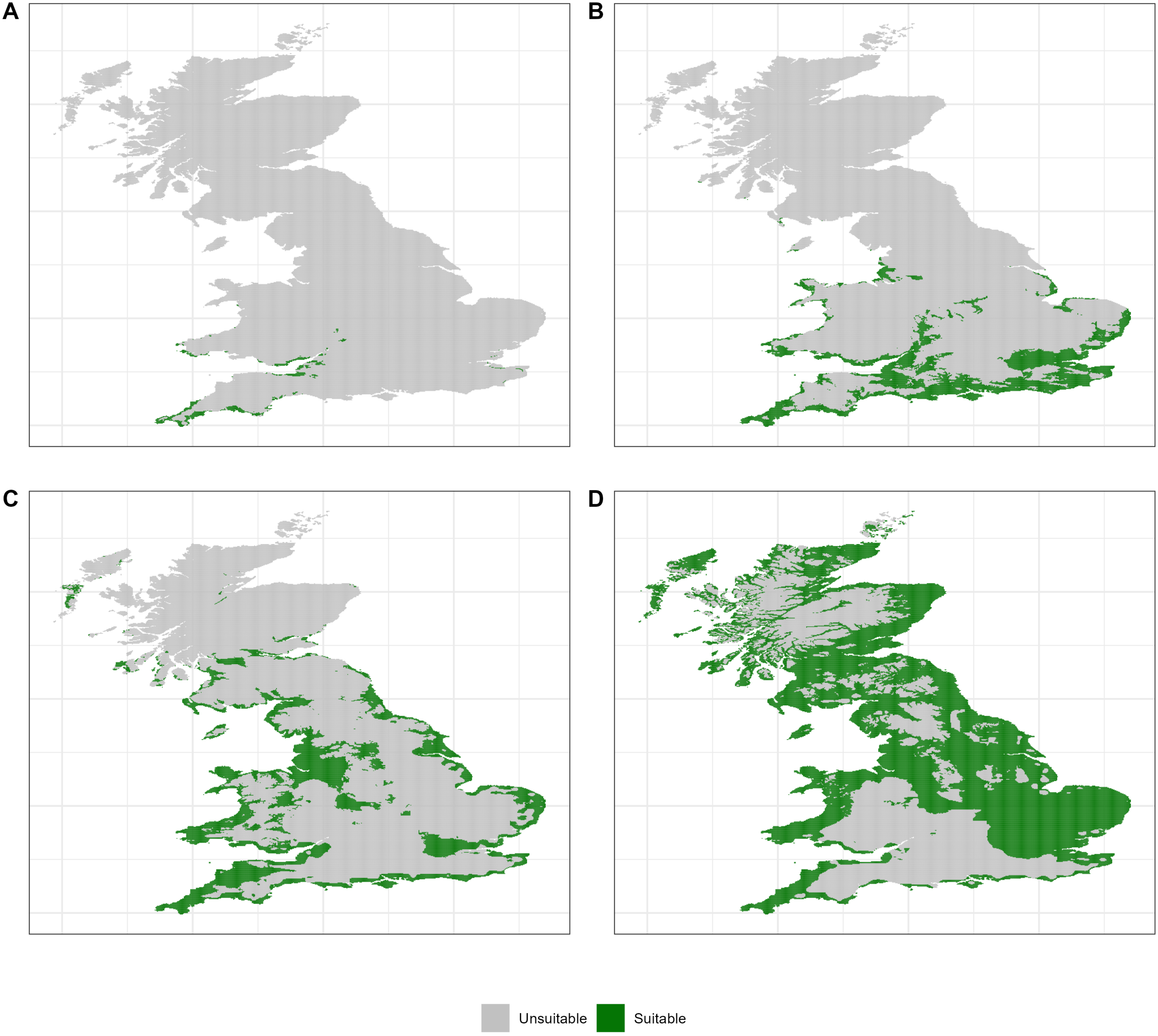

Figure S1. MaxEnt climate envelope maps for *Bombus humilis*. Showing climate envelope for 1980-89 **(A)**, 2010-19 **(B)**, and 2070-79 under RCP 4.5 **(C)** and RCP 8.5 **(D)**.
10th percentile training presence cloglog threshold = 0.149

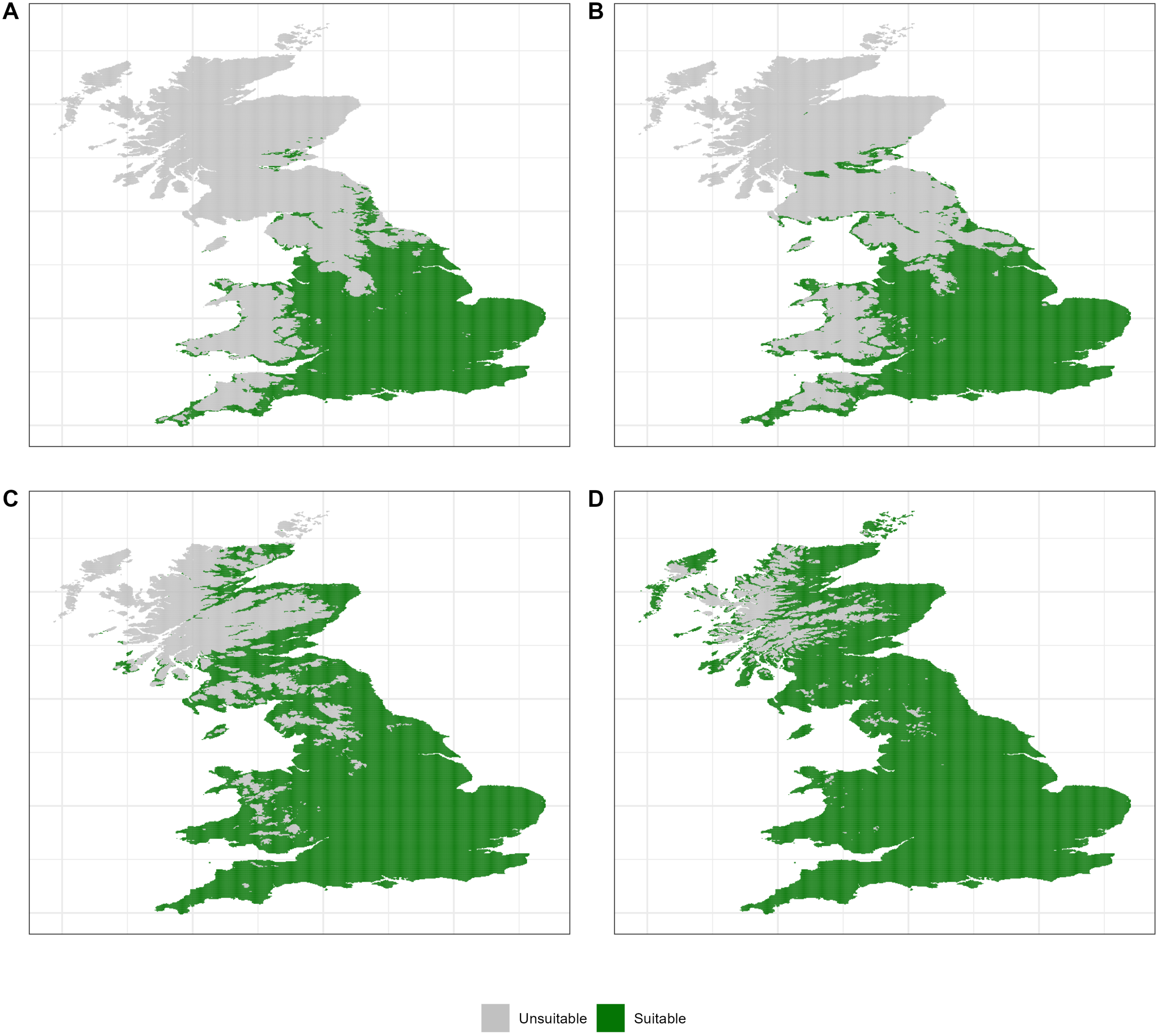

Figure S1. MaxEnt climate envelope maps for *Bombus lapidarius*. Showing climate envelope for 1980-89 **(A)**, 2010-19 **(B)**, and 2070-79 under RCP 4.5 **(C)** and RCP 8.5 **(D)**.
10th percentile training presence cloglog threshold = 0.3747

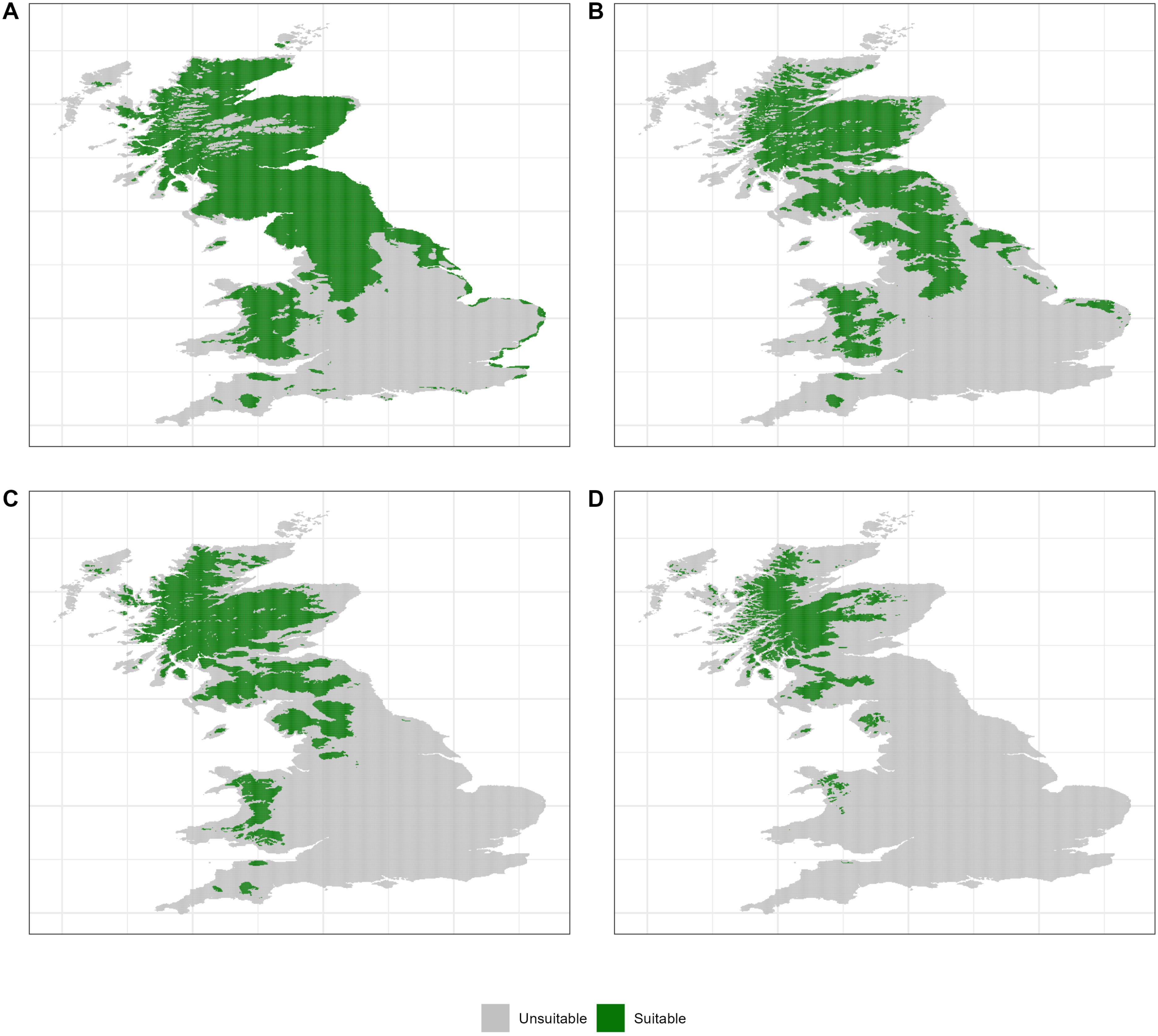

Figure S1. MaxEnt climate envelope maps for *Bombus monticola*. Showing climate envelope for 1980-89 **(A)**, 2010-19 **(B)**, and 2070-79 under RCP 4.5 **(C)** and RCP 8.5 **(D)**.
10th percentile training presence cloglog threshold = 0.244

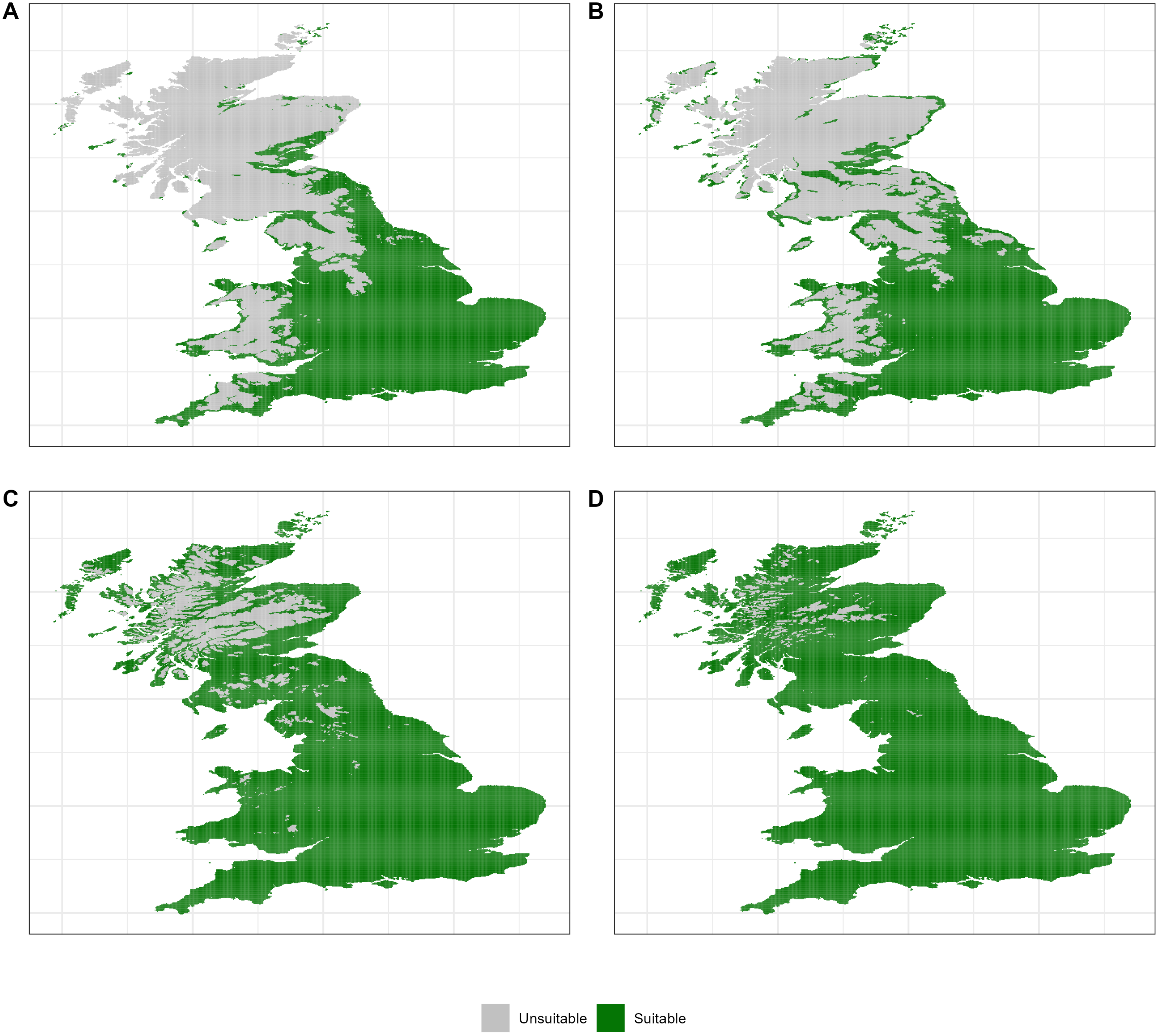

Figure S1. MaxEnt climate envelope maps for *Bombus pascuorum*. Showing climate envelope for 1980-89 **(A)**, 2010-19 **(B)**, and 2070-79 under RCP 4.5 **(C)** and RCP 8.5 **(D)**.
10th percentile training presence cloglog threshold = 0.3641

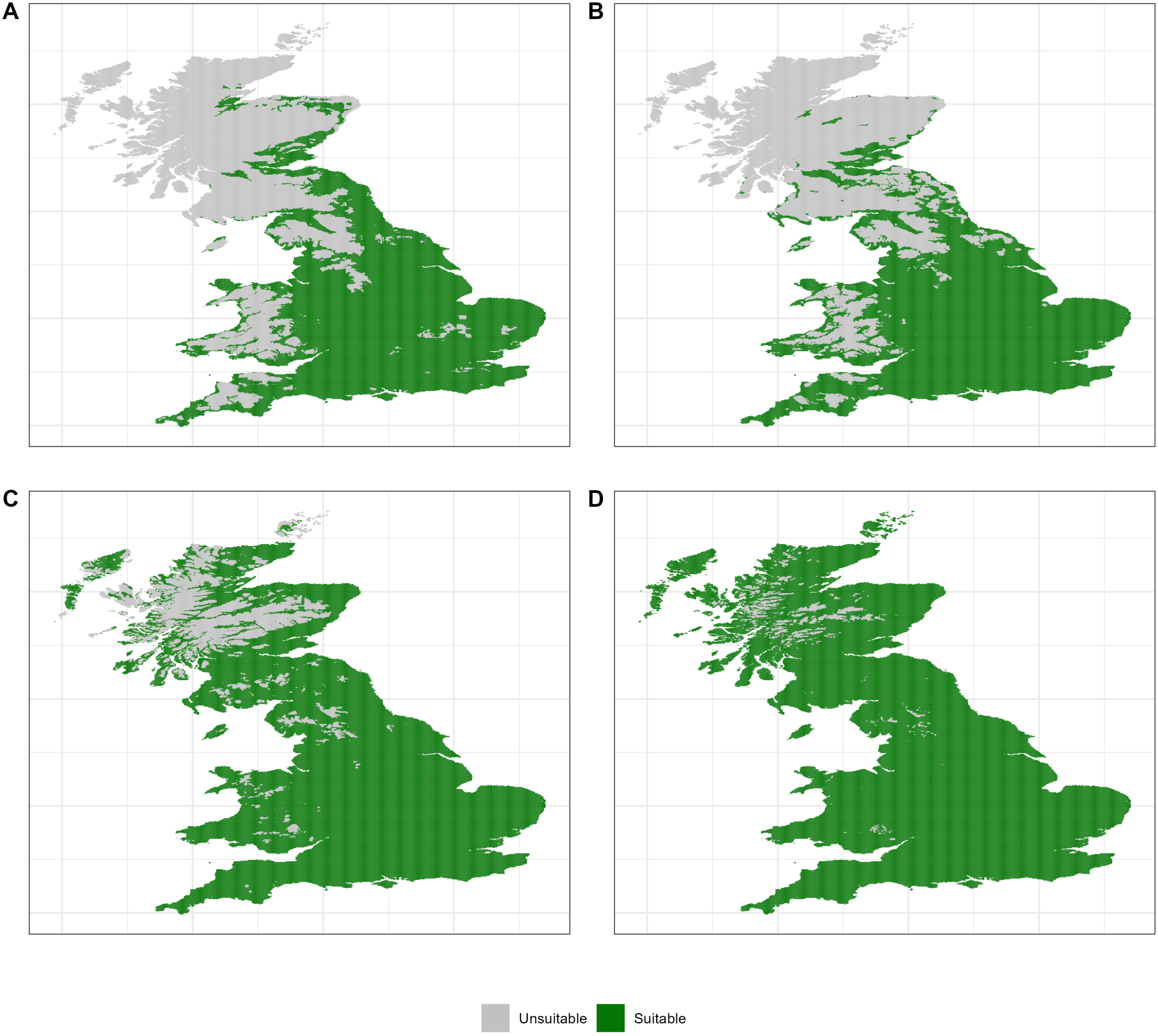

Figure S1. MaxEnt climate envelope maps for *Bombus pratorum*. Showing climate envelope for 1980-89 **(A)**, 2010-19 **(B)**, and 2070-79 under RCP 4.5 **(C)** and RCP 8.5 **(D)**.
10th percentile training presence cloglog threshold = 0.335

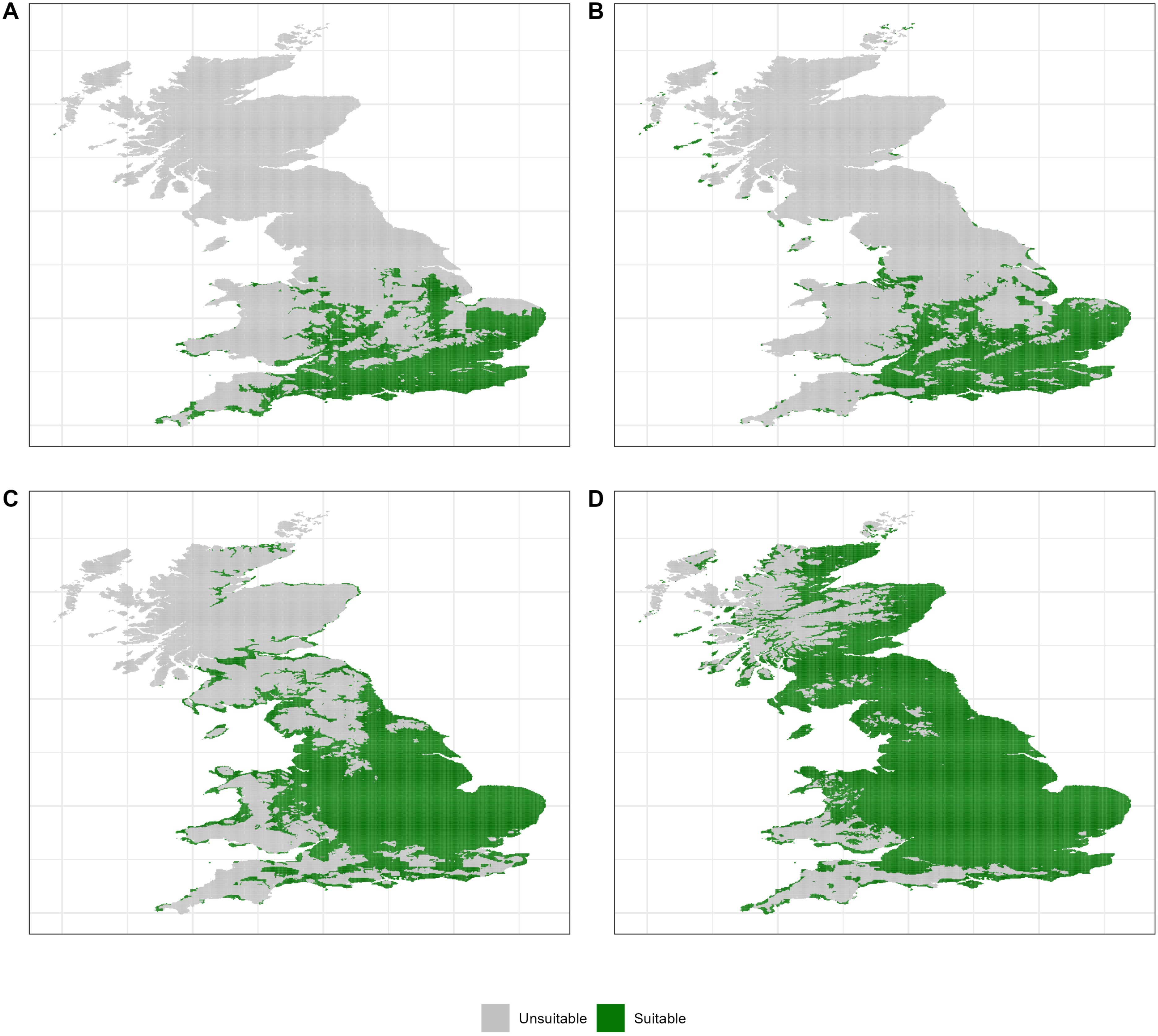

Figure S1. MaxEnt climate envelope maps for *Bombus ruderarius*. Showing climate envelope for 1980-89 **(A)**, 2010-19 **(B)**, and 2070-79 under RCP 4.5 **(C)** and RCP 8.5 **(D)**.
10th percentile training presence cloglog threshold = 0.2903

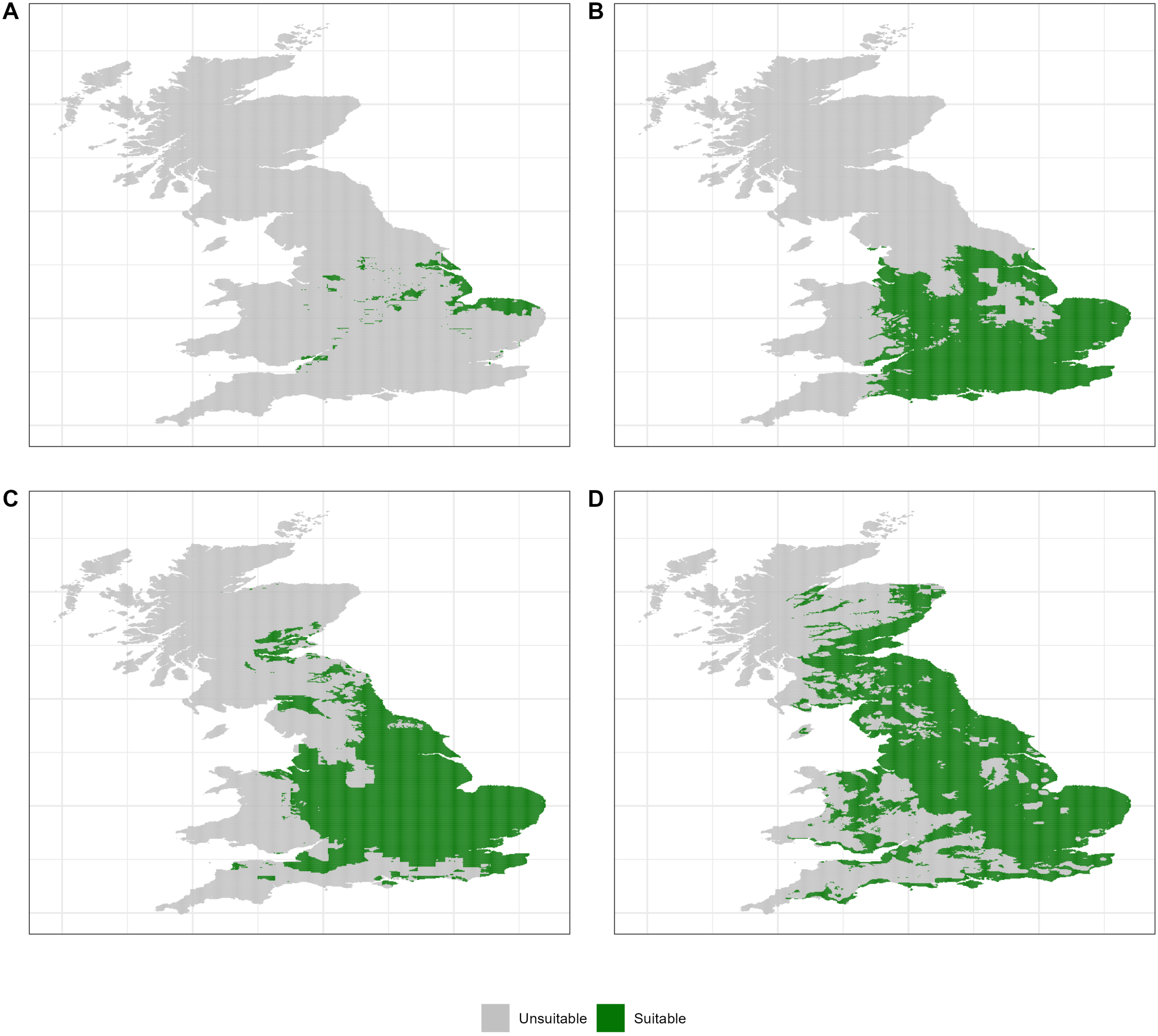

Figure S1. MaxEnt climate envelope maps for *Bombus rupestris*. Showing climate envelope for 1980-89 **(A)**, 2010-19 **(B)**, and 2070-79 under RCP 4.5 **(C)** and RCP 8.5 **(D)**.
10th percentile training presence cloglog threshold = 0.4458

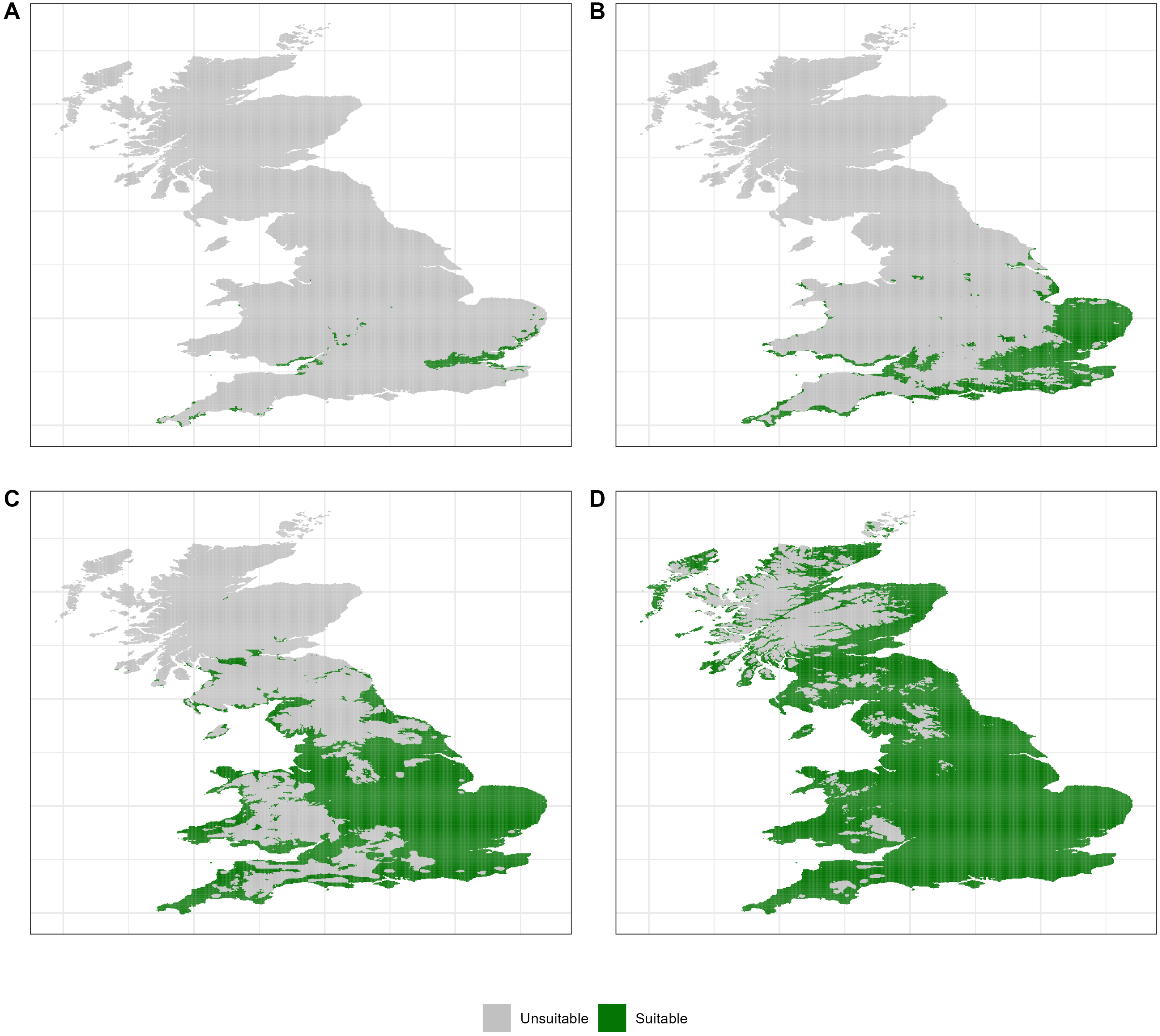

Figure S1. MaxEnt climate envelope maps for *Dasypoda hirtipes*. Showing climate envelope for 1980-89 **(A)**, 2010-19 **(B)**, and 2070-79 under RCP 4.5 **(C)** and RCP 8.5 **(D)**.
10th percentile training presence cloglog threshold = 0.2772

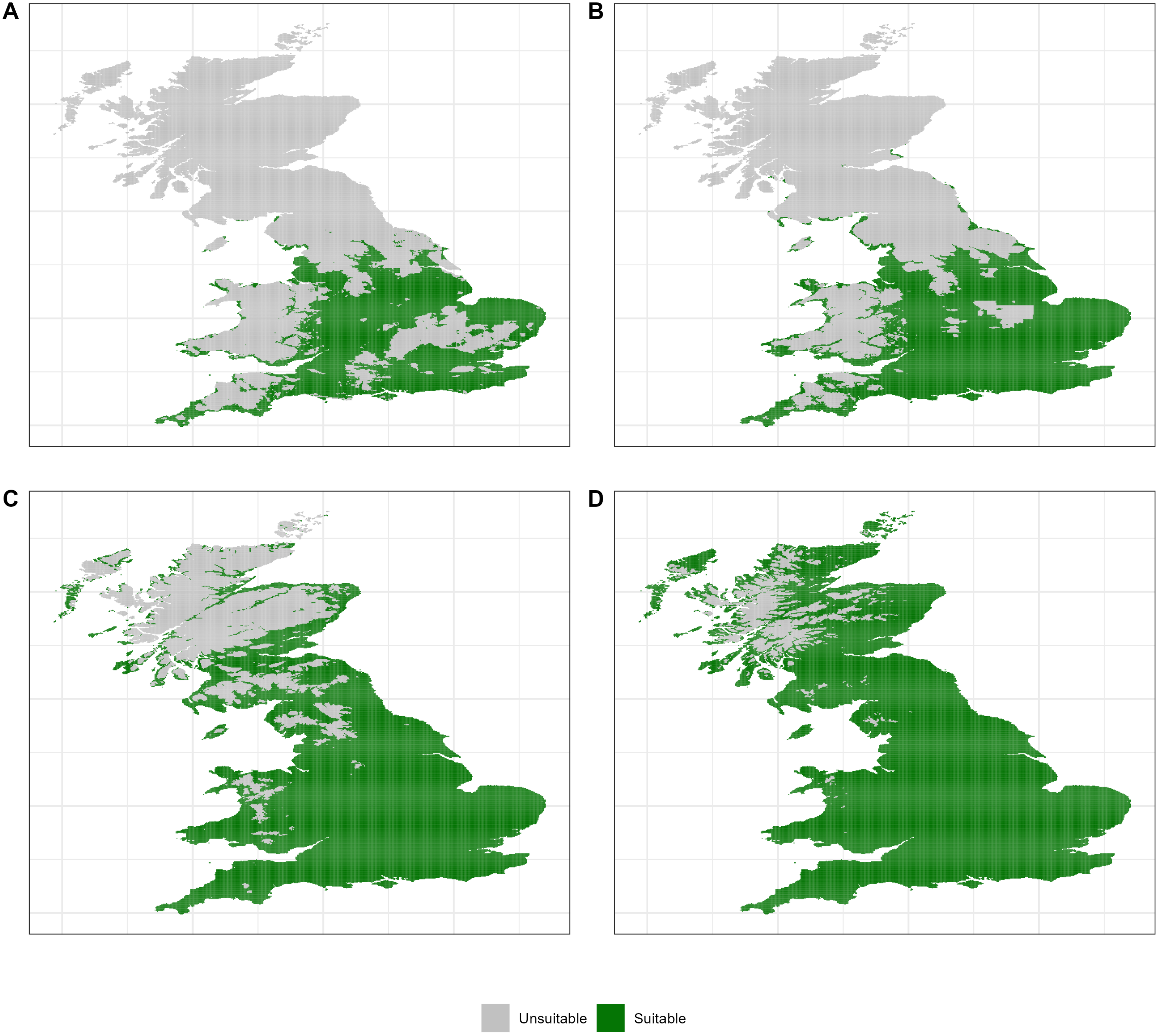

Figure S1. MaxEnt climate envelope maps for *Epeolus cruciger*. Showing climate envelope for 1980-89 **(A)**, 2010-19 **(B)**, and 2070-79 under RCP 4.5 **(C)** and RCP 8.5 **(D)**.
10th percentile training presence cloglog threshold = 0.2362

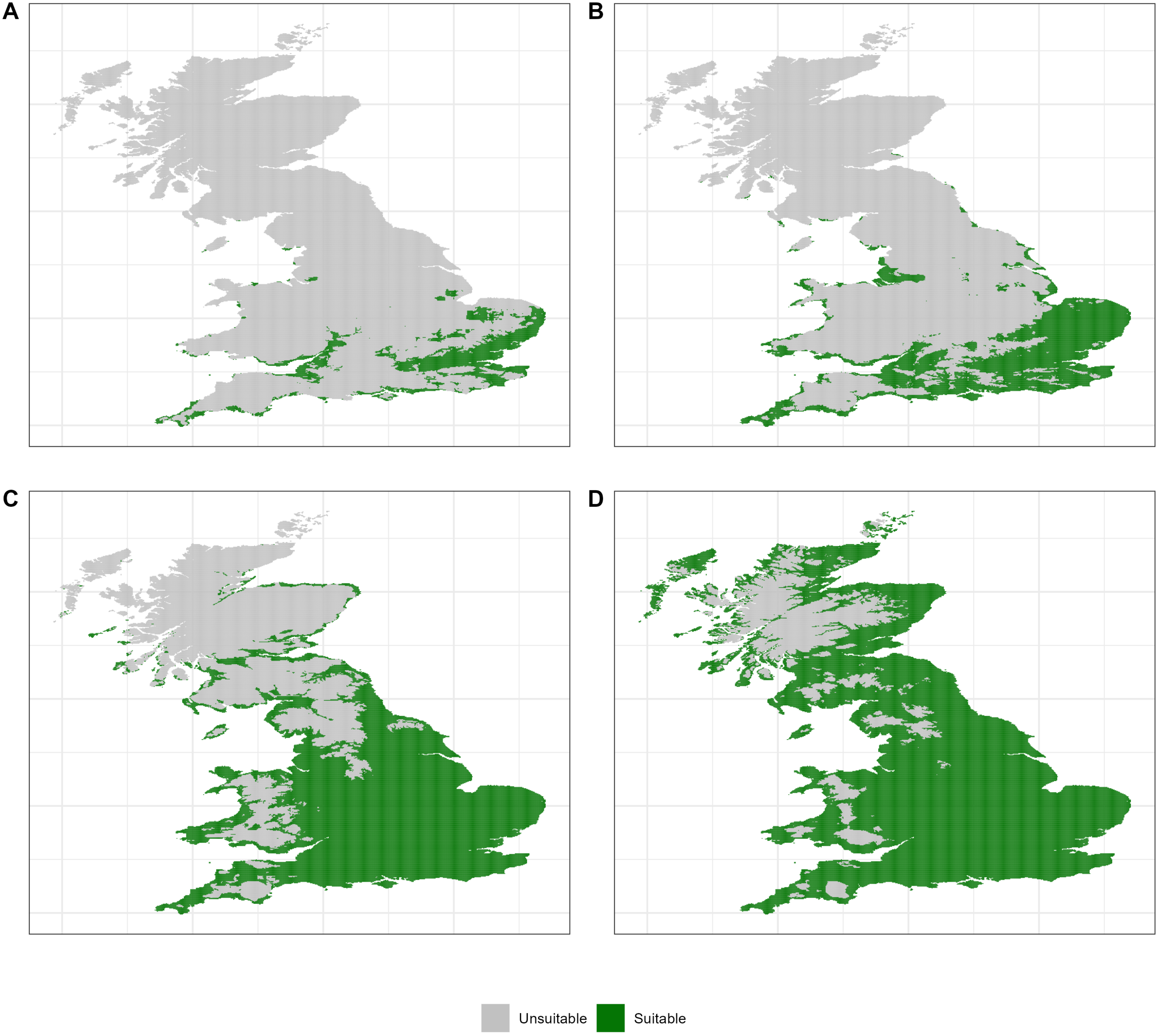

Figure S1. MaxEnt climate envelope maps for *Epeolus variegatus*. Showing climate envelope for 1980-89 **(A)**, 2010-19 **(B)**, and 2070-79 under RCP 4.5 **(C)** and RCP 8.5 **(D)**.
10th percentile training presence cloglog threshold = 0.2674

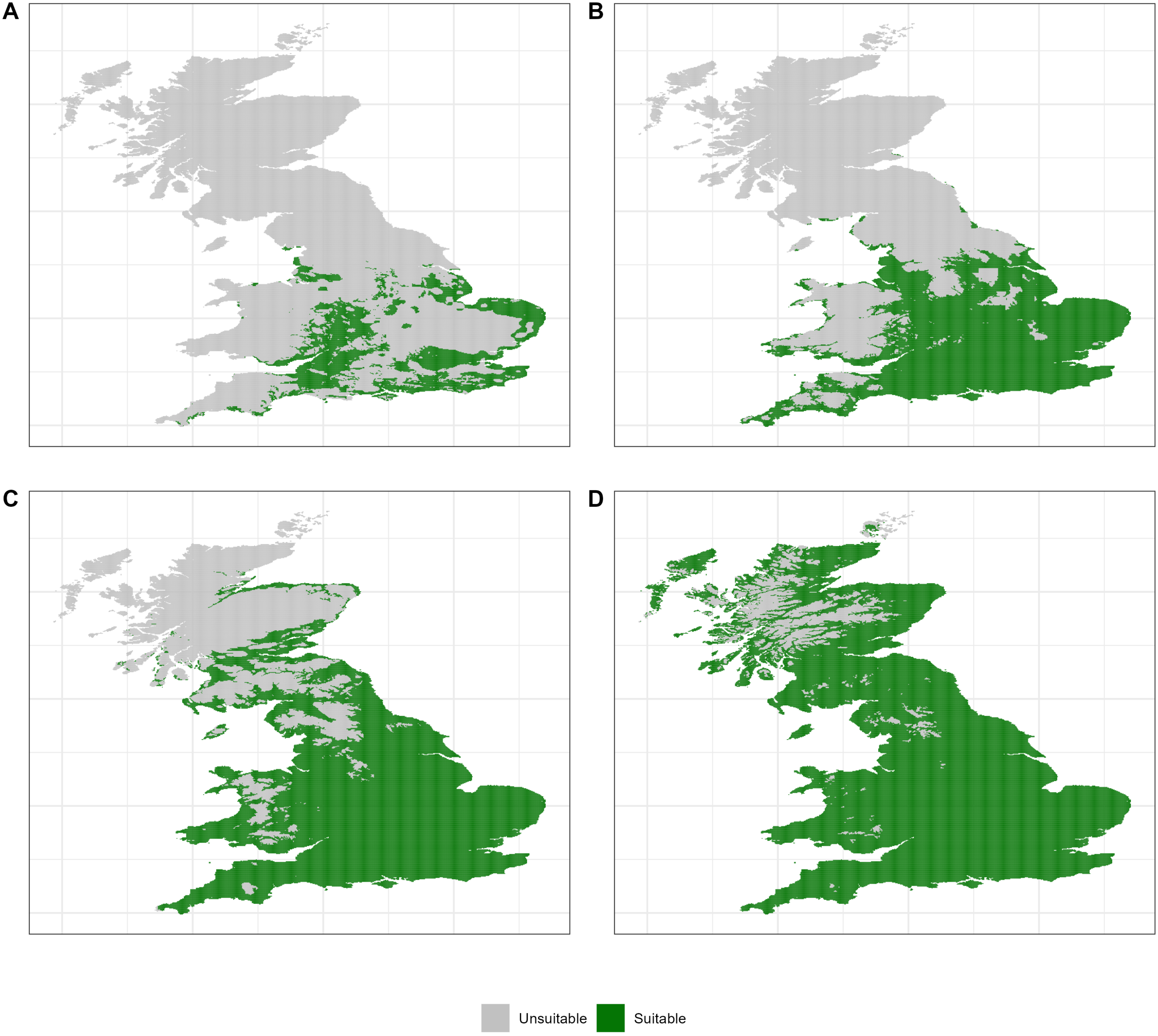

Figure S1. MaxEnt climate envelope maps for *Halictus tumulorum*. Showing climate envelope for 1980-89 **(A)**, 2010-19 **(B)**, and 2070-79 under RCP 4.5 **(C)** and RCP 8.5 **(D)**.
10th percentile training presence cloglog threshold = 0.3616

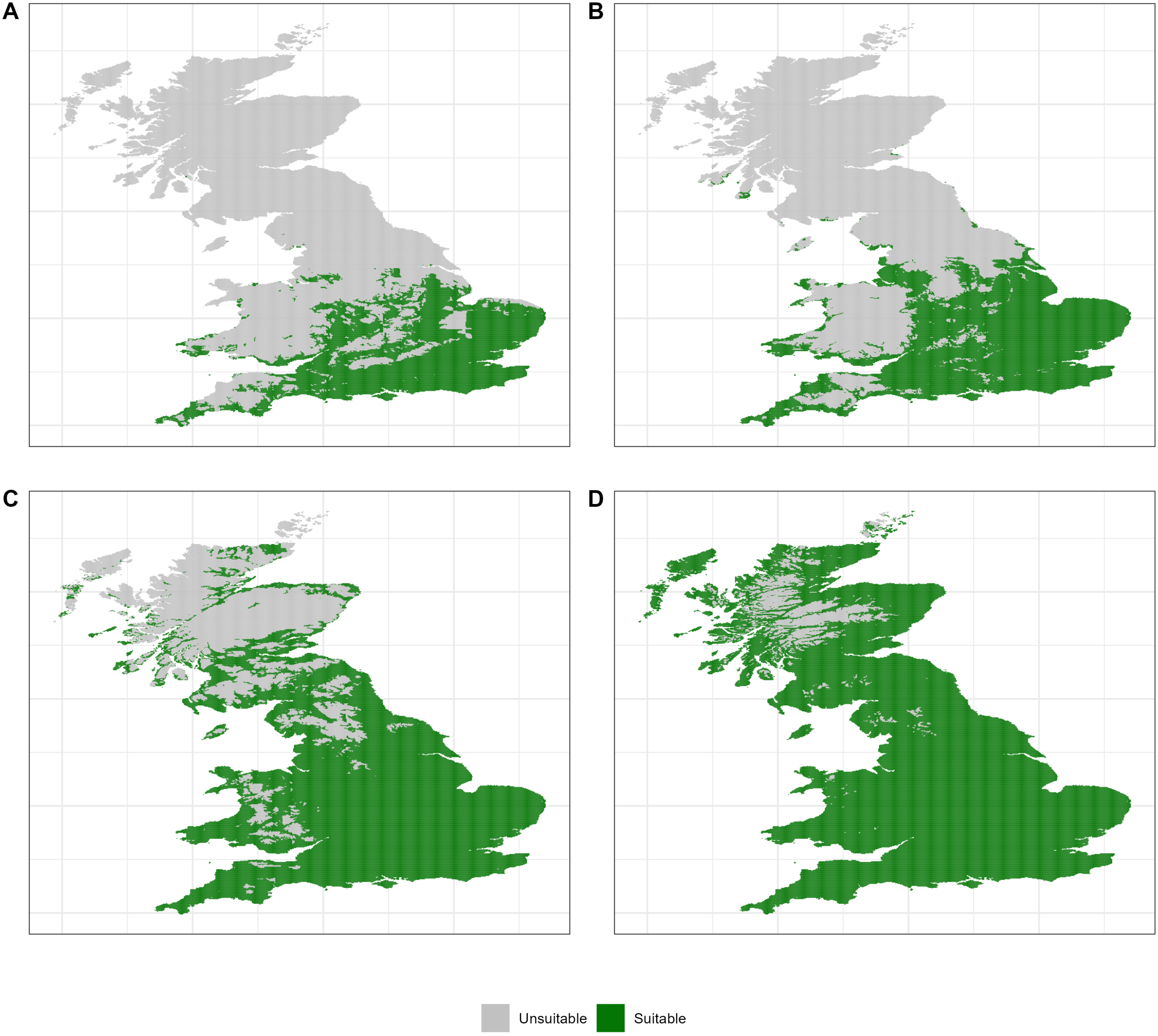

Figure S1. MaxEnt climate envelope maps for *Hylaeus brevicornis*. Showing climate envelope for 1980-89 **(A)**, 2010-19 **(B)**, and 2070-79 under RCP 4.5 **(C)** and RCP 8.5 **(D)**.
10th percentile training presence cloglog threshold = 0.2703

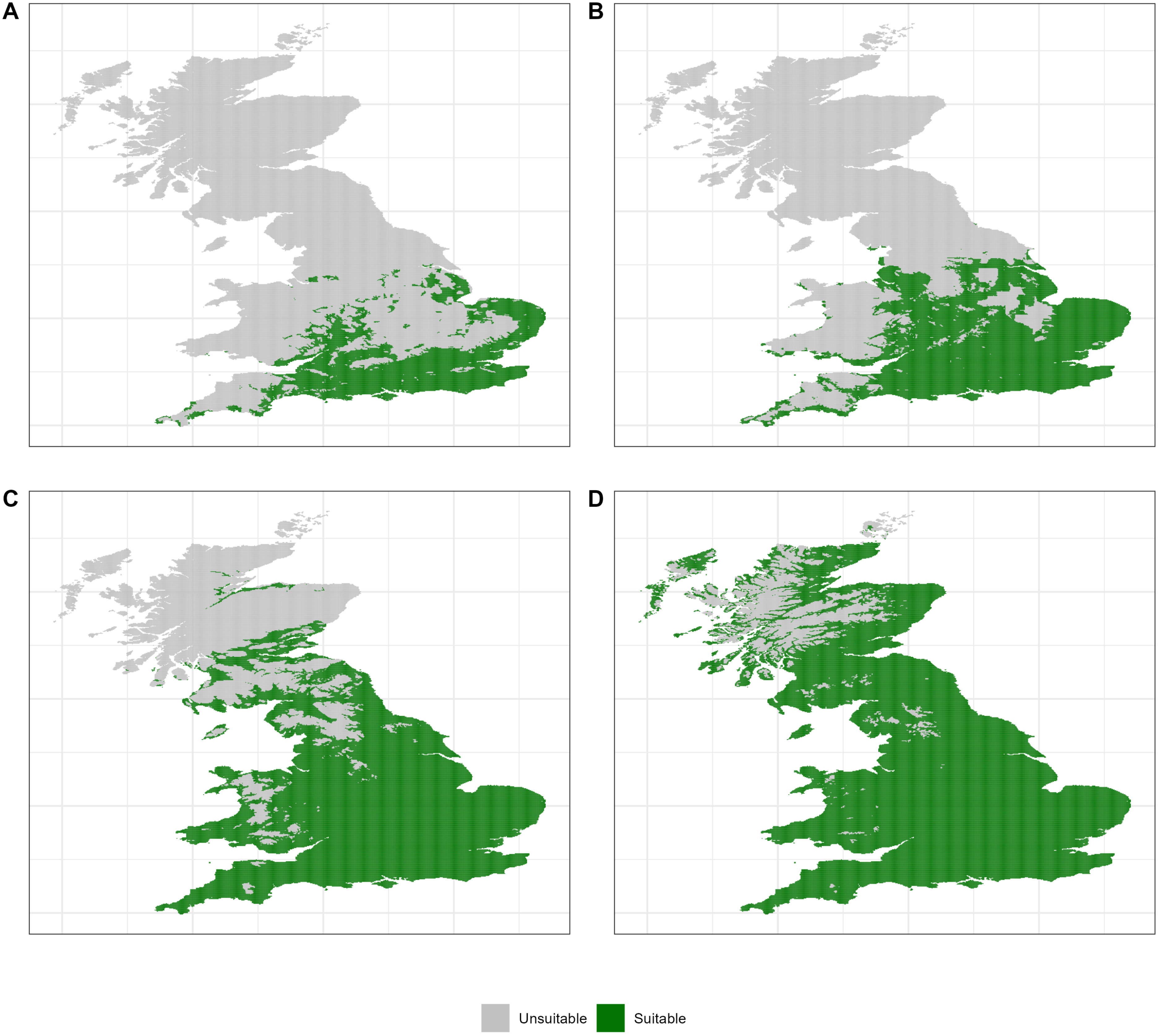

Figure S1. MaxEnt climate envelope maps for *Hylaeus communis*. Showing climate envelope for 1980-89 **(A)**, 2010-19 **(B)**, and 2070-79 under RCP 4.5 **(C)** and RCP 8.5 **(D)**.
10th percentile training presence cloglog threshold = 0.3506

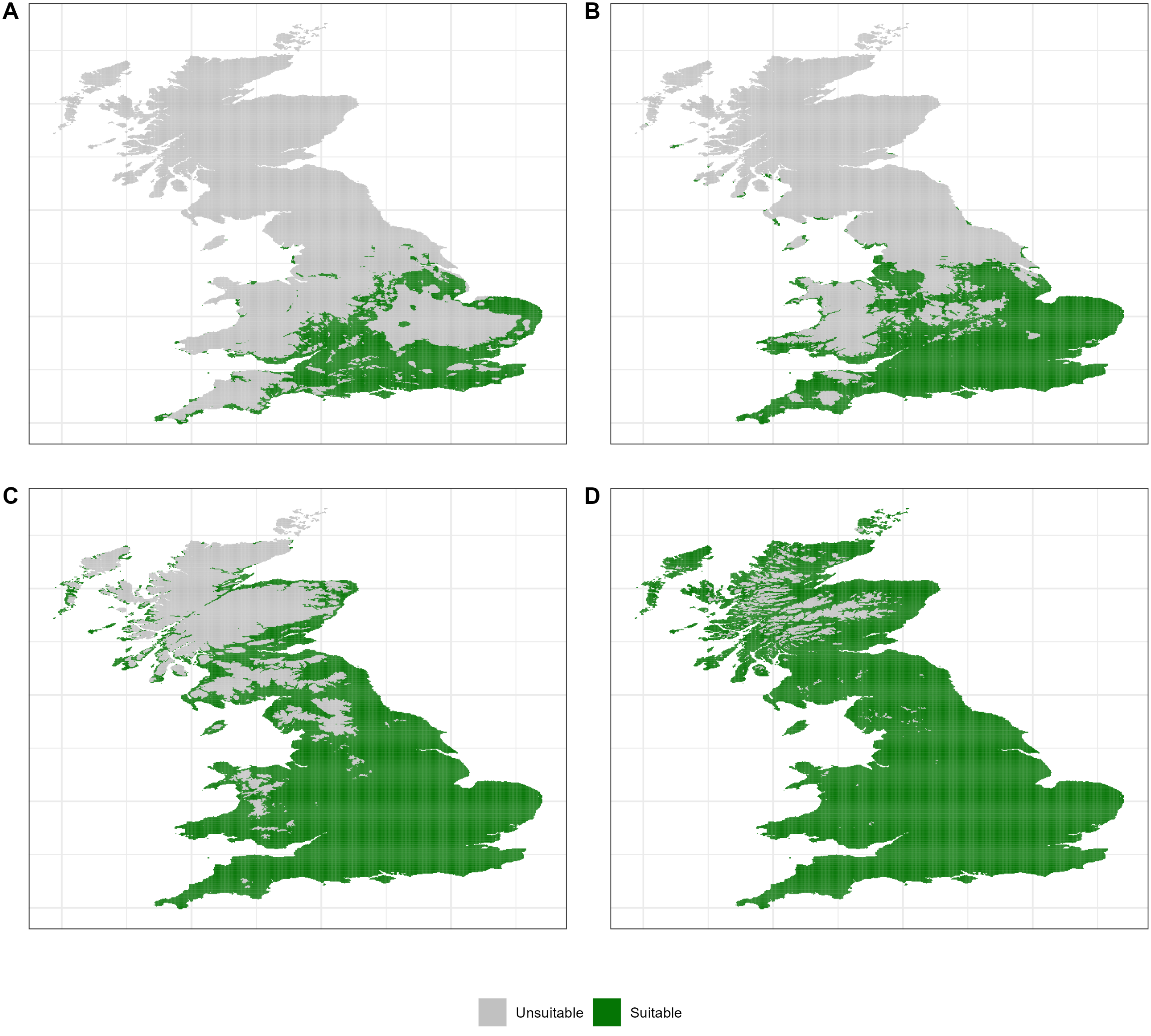

Figure S1. MaxEnt climate envelope maps for *Hylaeus confusus*. Showing climate envelope for 1980-89 **(A)**, 2010-19 **(B)**, and 2070-79 under RCP 4.5 **(C)** and RCP 8.5 **(D)**.
10th percentile training presence cloglog threshold = 0.2998

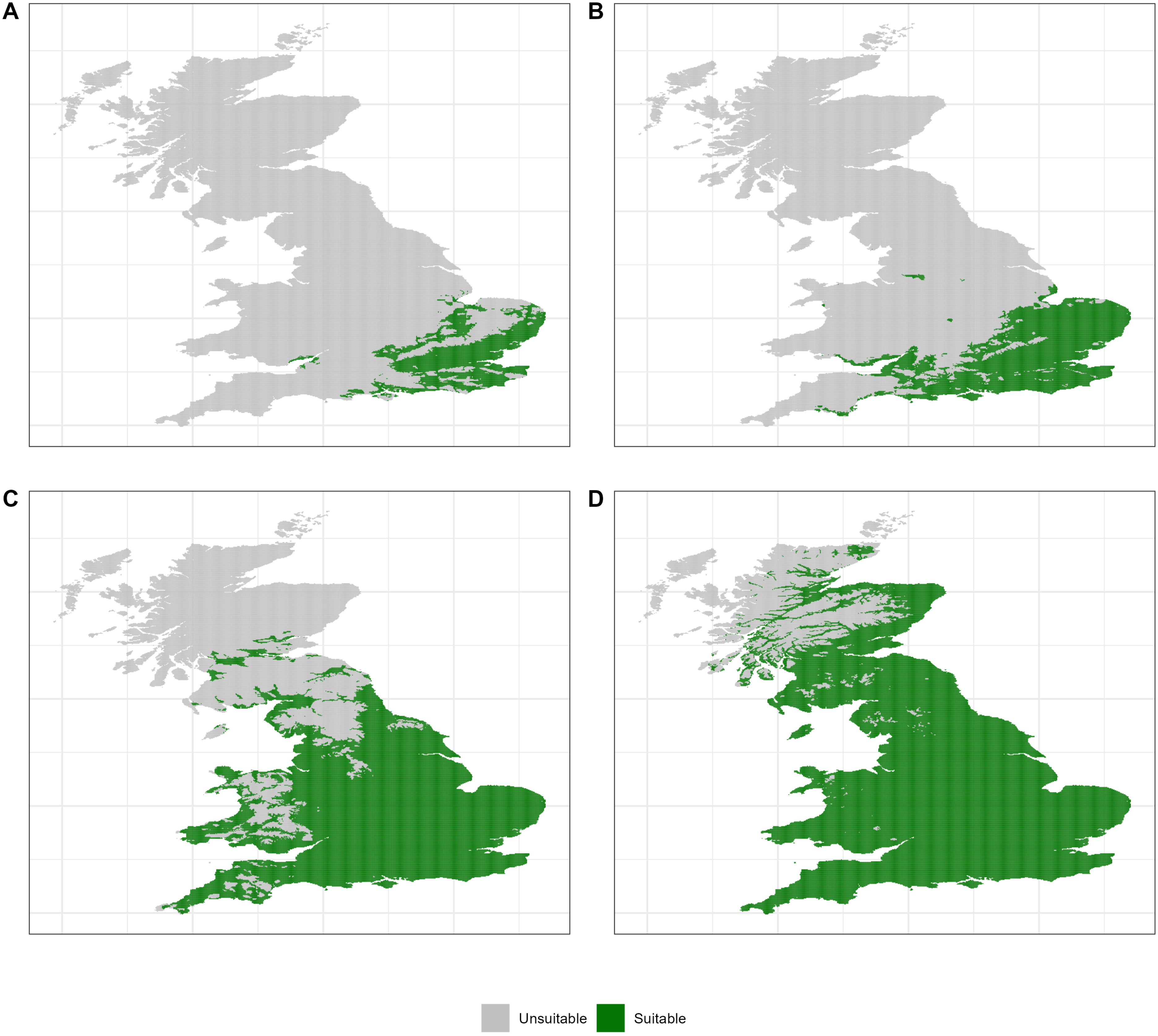

Figure S1. MaxEnt climate envelope maps for *Hylaeus dilatatus*. Showing climate envelope for 1980-89 **(A)**, 2010-19 **(B)**, and 2070-79 under RCP 4.5 **(C)** and RCP 8.5 **(D)**.
10th percentile training presence cloglog threshold = 0.2958

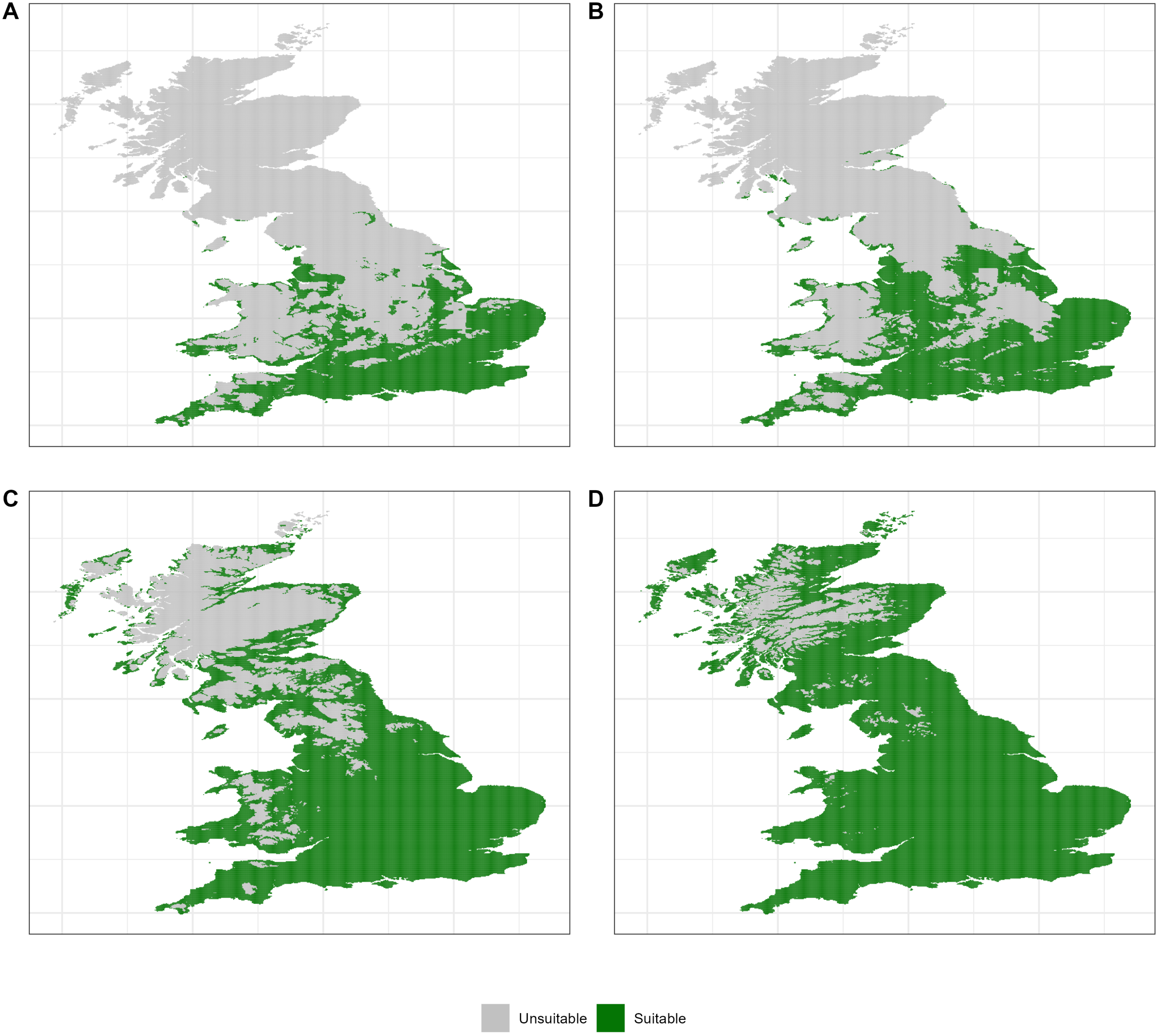

Figure S1. MaxEnt climate envelope maps for *Hylaeus hyalinatus*. Showing climate envelope for 1980-89 **(A)**, 2010-19 **(B)**, and 2070-79 under RCP 4.5 **(C)** and RCP 8.5 **(D)**.
10th percentile training presence cloglog threshold = 0.3138

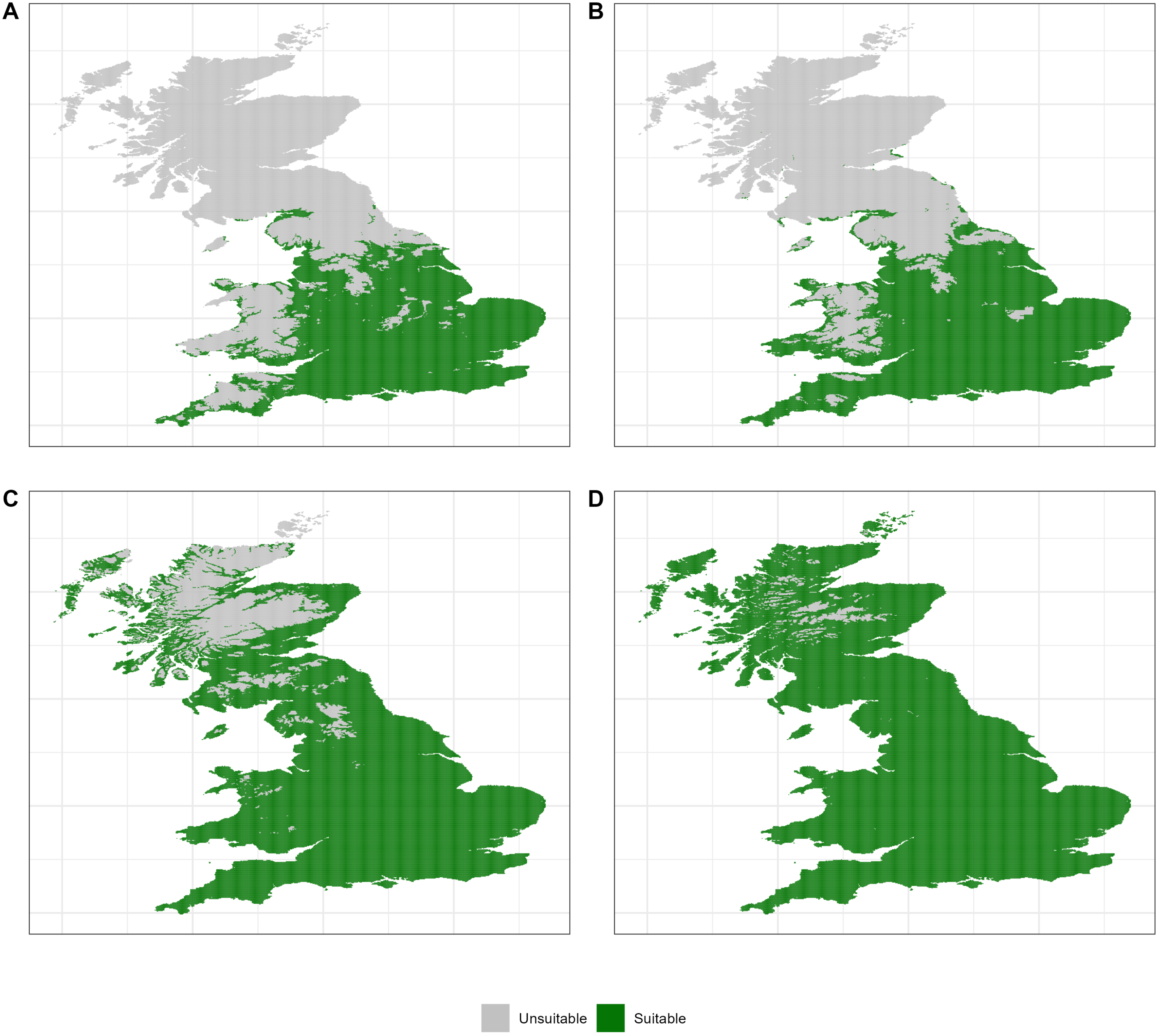

Figure S1. MaxEnt climate envelope maps for *Lasioglossum calceatum*. Showing climate envelope for 1980-89 **(A)**, 2010-19 **(B)**, and 2070-79 under RCP 4.5 **(C)** and RCP 8.5 **(D)**.
10th percentile training presence cloglog threshold = 0.3104

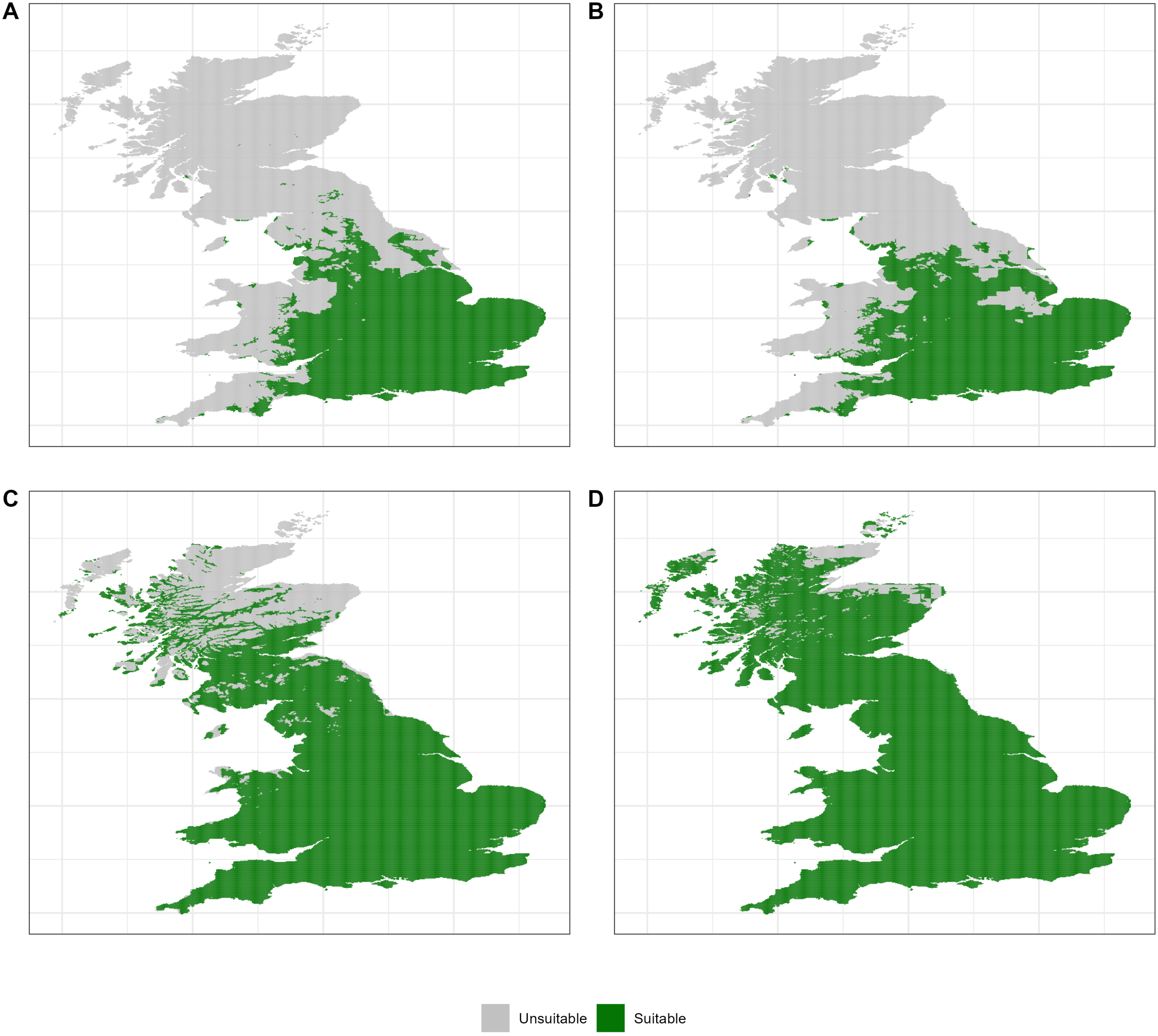

Figure S1. MaxEnt climate envelope maps for *Lasioglossum fulvicorne*. Showing climate envelope for 1980-89 **(A)**, 2010-19 **(B)**, and 2070-79 under RCP 4.5 **(C)** and RCP 8.5 **(D)**.
10th percentile training presence cloglog threshold = 0.2702

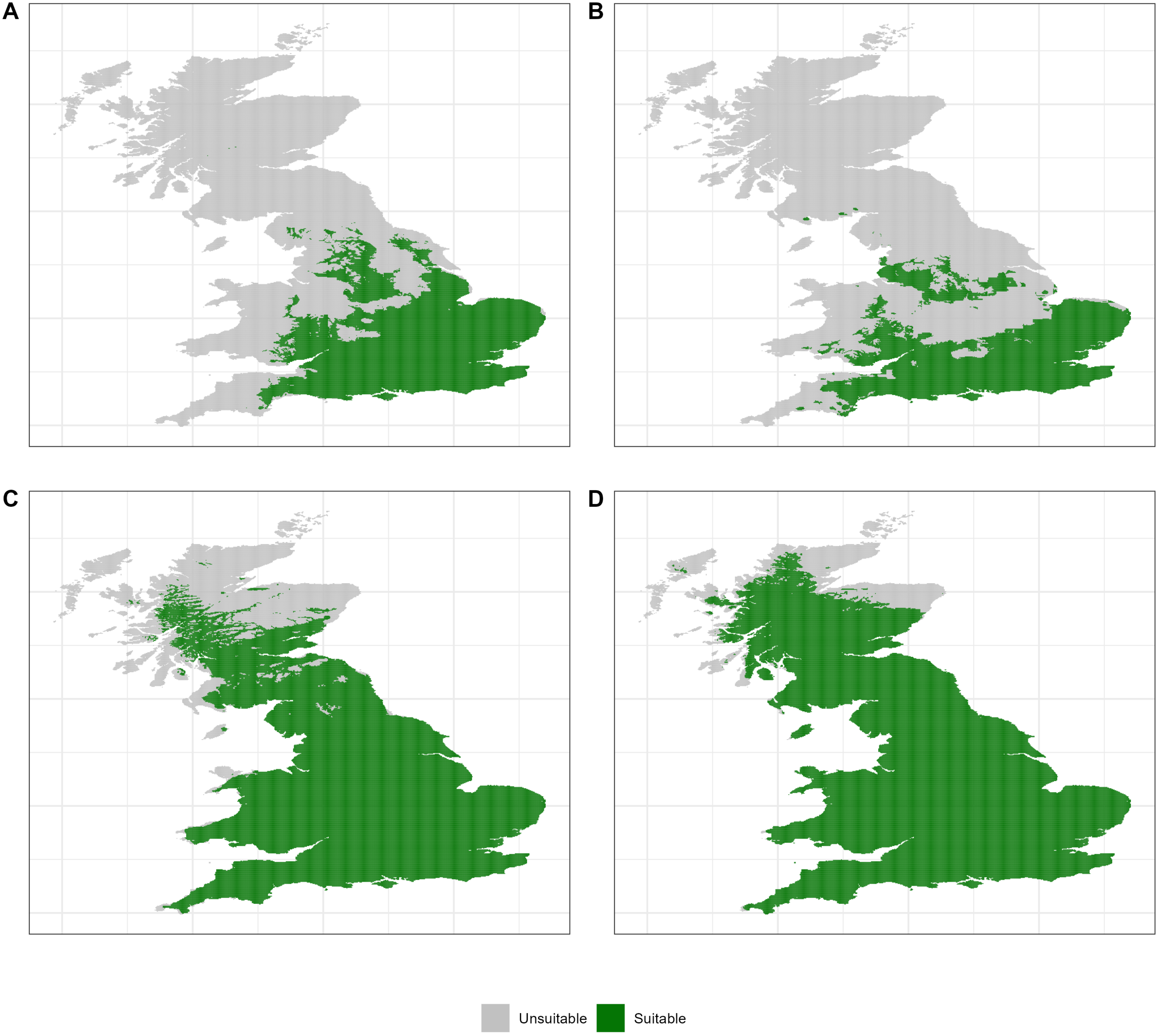

Figure S1. MaxEnt climate envelope maps for *Lasioglossum laevigatum*. Showing climate envelope for 1980-89 **(A)**, 2010-19 **(B)**, and 2070-79 under RCP 4.5 **(C)** and RCP 8.5 **(D)**.
10th percentile training presence cloglog threshold = 0.2103

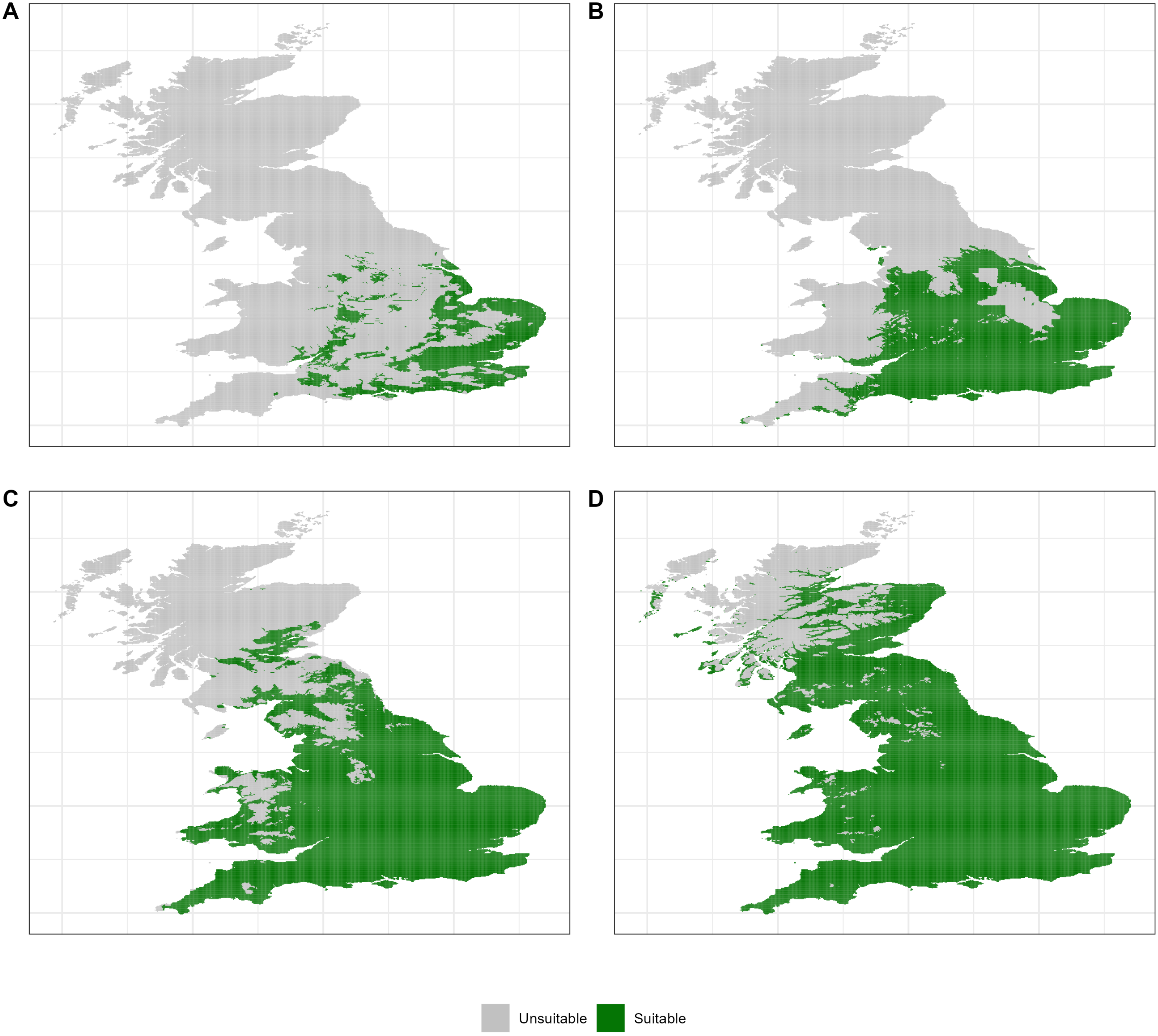

Figure S1. MaxEnt climate envelope maps for *Lasioglossum lativentre*. Showing climate envelope for 1980-89 **(A)**, 2010-19 **(B)**, and 2070-79 under RCP 4.5 **(C)** and RCP 8.5 **(D)**.
10th percentile training presence cloglog threshold = 0.3545

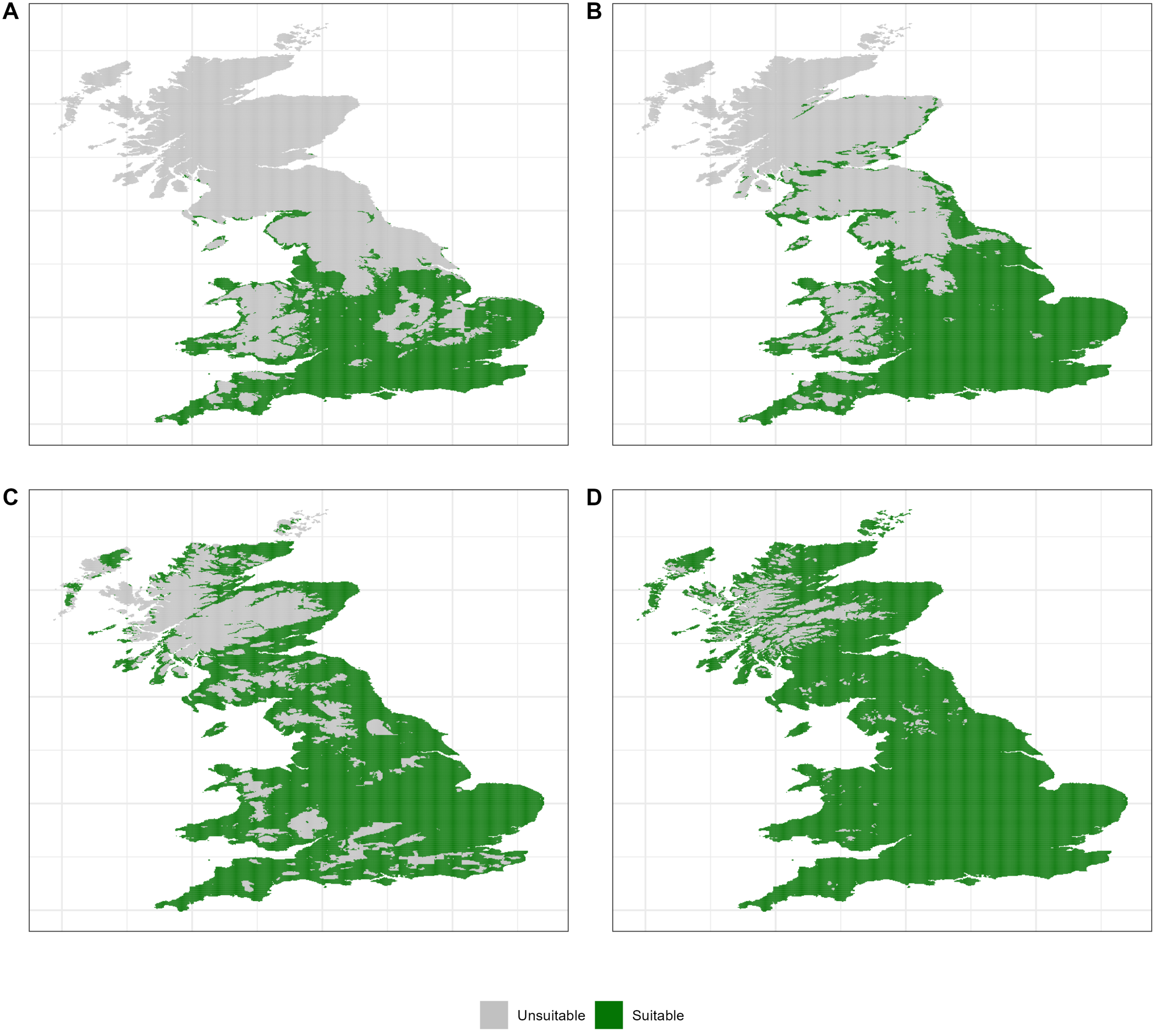

Figure S1. MaxEnt climate envelope maps for *Lasioglossum leucopus*. Showing climate envelope for 1980-89 **(A)**, 2010-19 **(B)**, and 2070-79 under RCP 4.5 **(C)** and RCP 8.5 **(D)**.
10th percentile training presence cloglog threshold = 0.2913

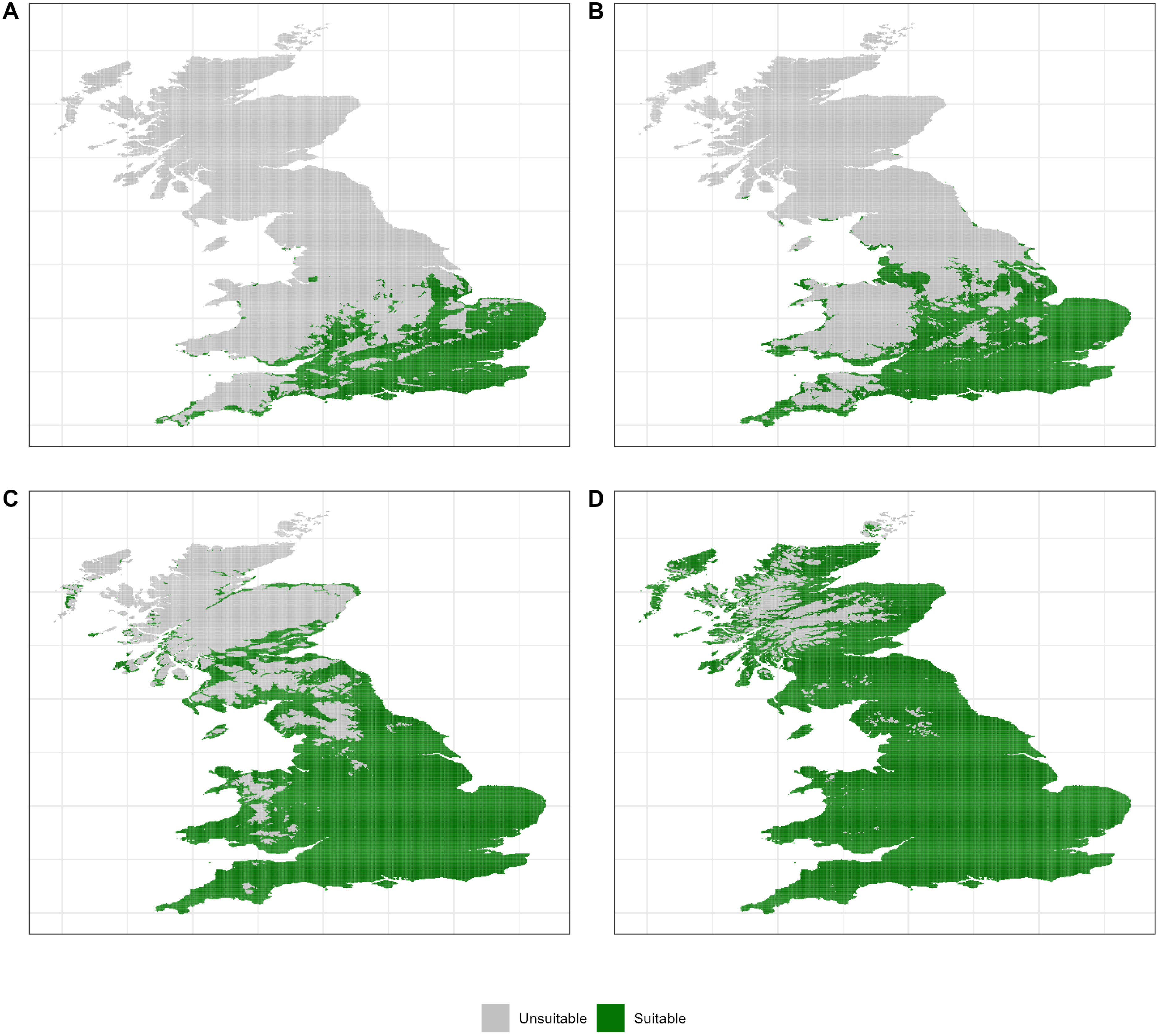

Figure S1. MaxEnt climate envelope maps for *Lasioglossum leucozonium*. Showing climate envelope for 1980-89 **(A)**, 2010-19 **(B)**, and 2070-79 under RCP 4.5 **(C)** and RCP 8.5 **(D)**.
10th percentile training presence cloglog threshold = 0.3057

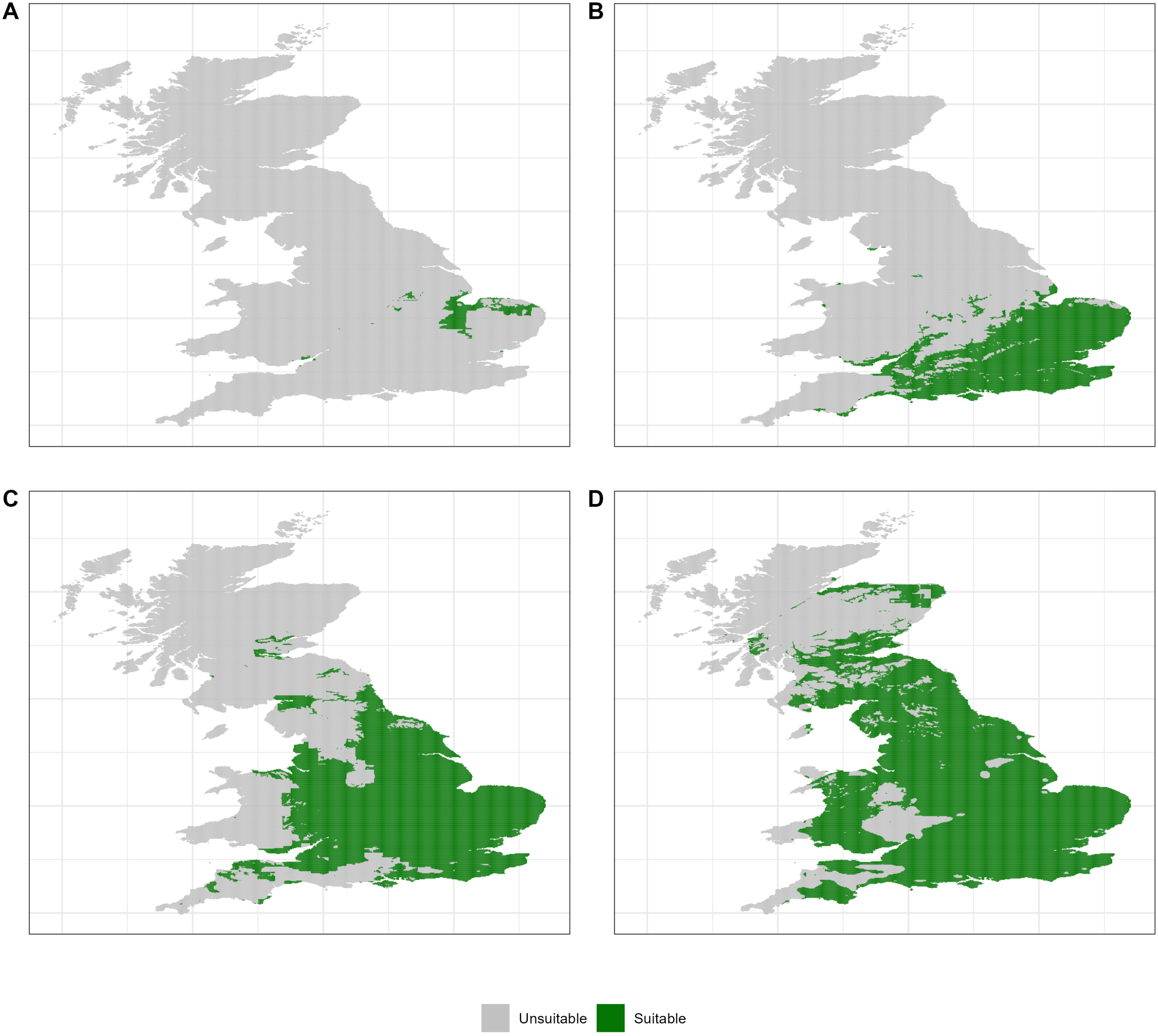

Figure S1. MaxEnt climate envelope maps for *Lasioglossum malachurum*. Showing climate envelope for 1980-89 **(A)**, 2010-19 **(B)**, and 2070-79 under RCP 4.5 **(C)** and RCP 8.5 **(D)**.
10th percentile training presence cloglog threshold = 0.3252

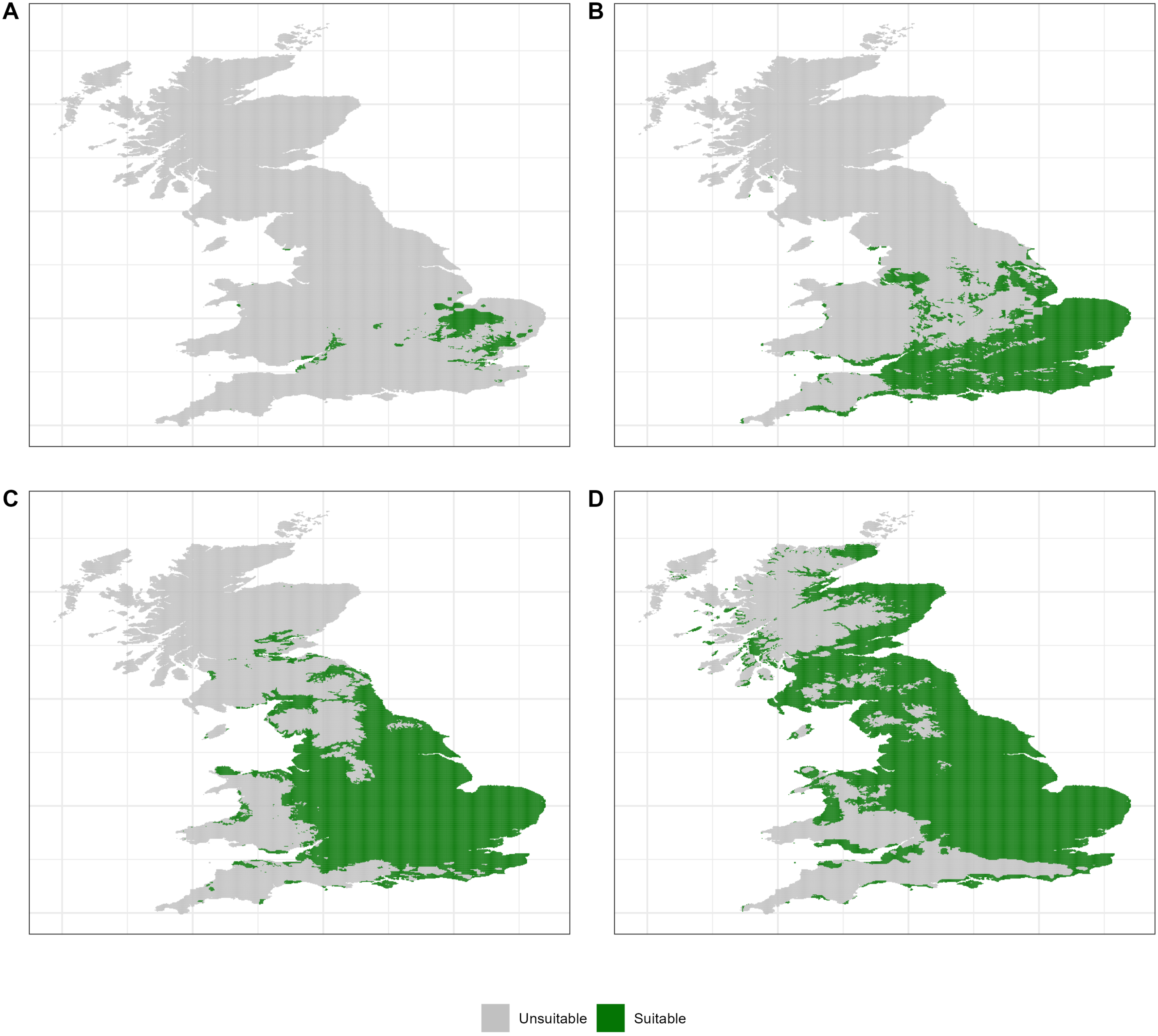

Figure S1. MaxEnt climate envelope maps for *Lasioglossum minutissimum*. Showing climate envelope for 1980-89 **(A)**, 2010-19 **(B)**, and 2070-79 under RCP 4.5 **(C)** and RCP 8.5 **(D)**.
10th percentile training presence cloglog threshold = 0.2861

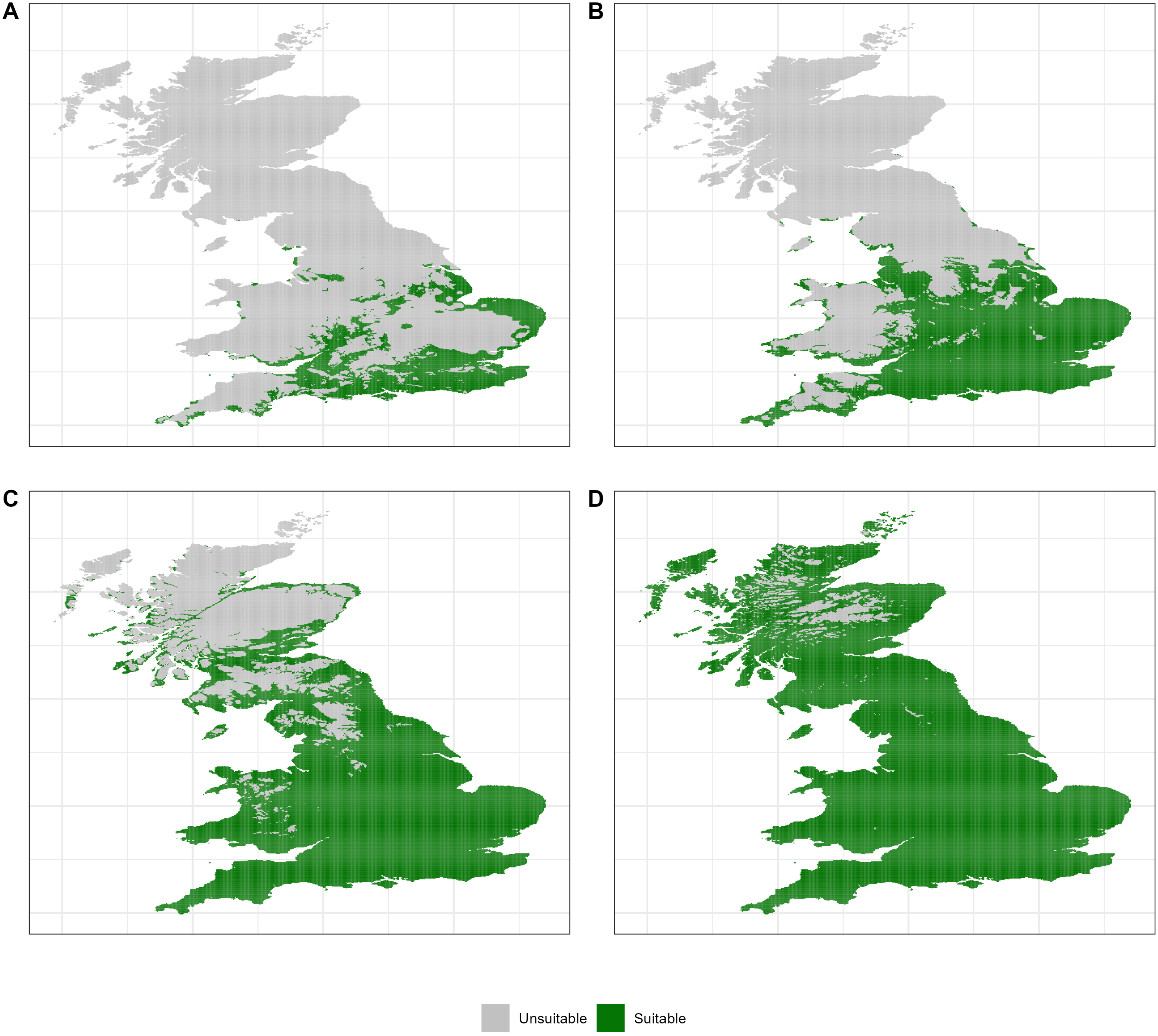

Figure S1. MaxEnt climate envelope maps for *Lasioglossum morio*. Showing climate envelope for 1980-89 **(A)**, 2010-19 **(B)**, and 2070-79 under RCP 4.5 **(C)** and RCP 8.5 **(D)**.
10th percentile training presence cloglog threshold = 0.3192

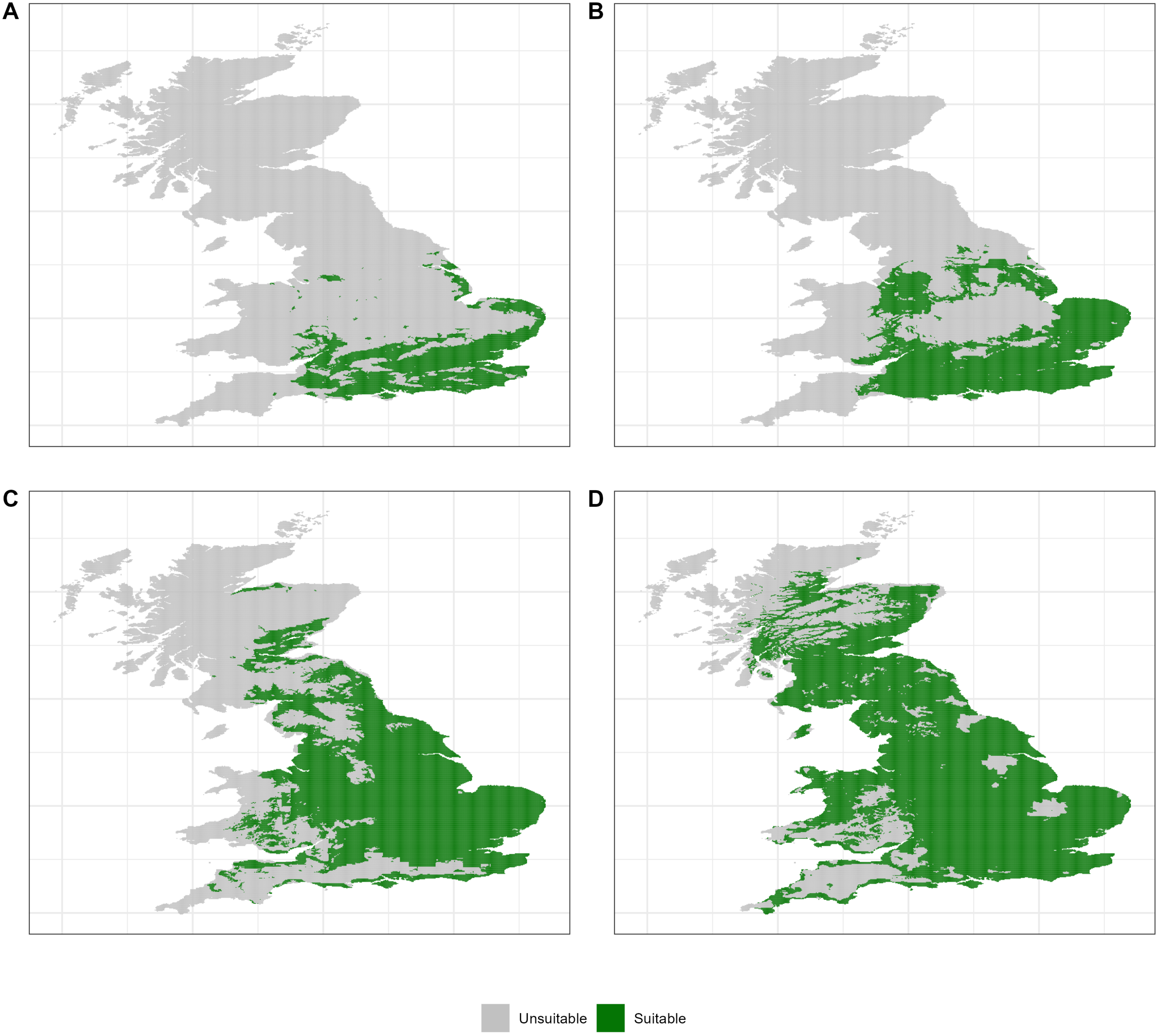

Figure S1. MaxEnt climate envelope maps for *Lasioglossum parvulum*. Showing climate envelope for 1980-89 **(A)**, 2010-19 **(B)**, and 2070-79 under RCP 4.5 **(C)** and RCP 8.5 **(D)**.
10th percentile training presence cloglog threshold = 0.3439

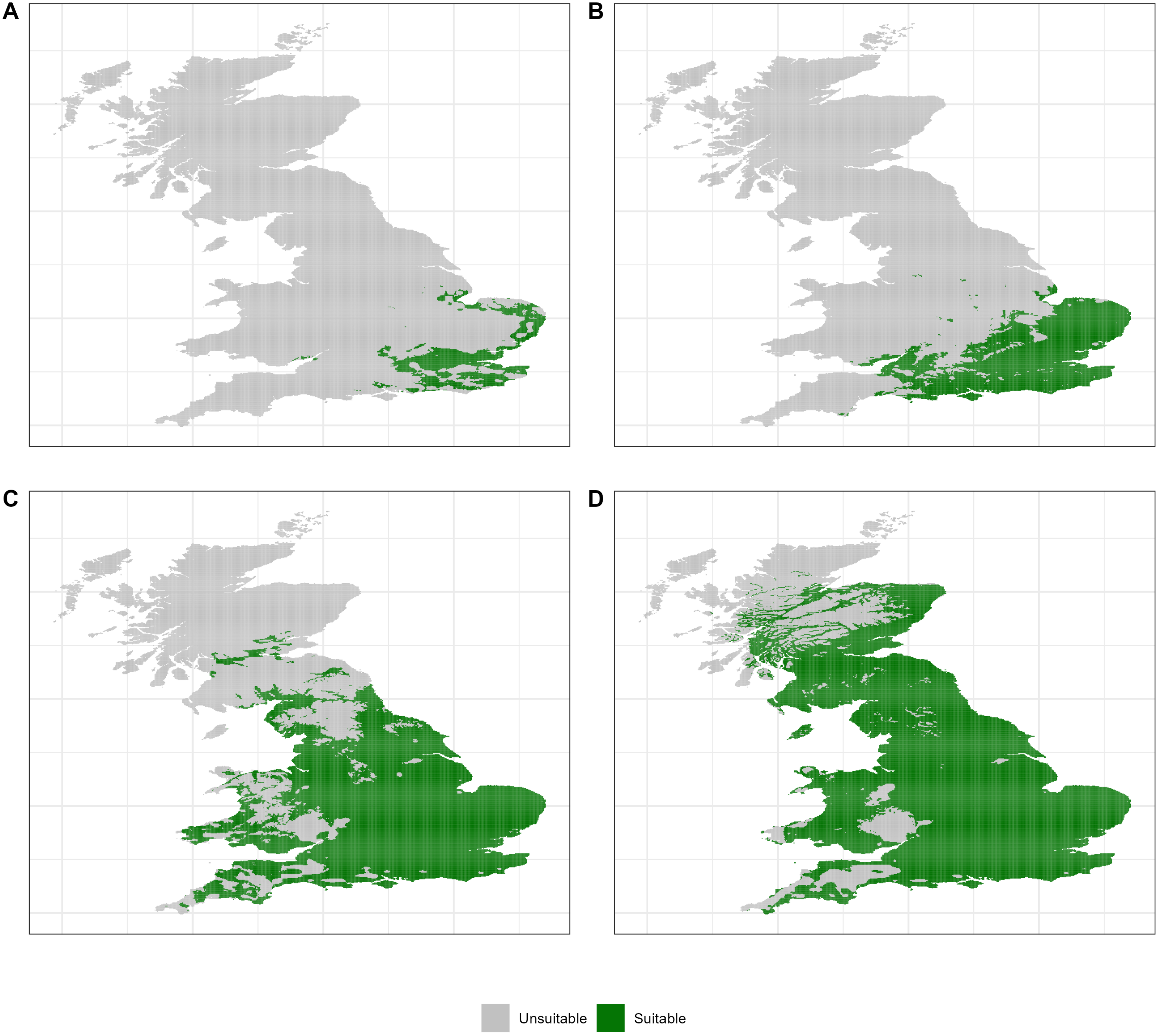

Figure S1. MaxEnt climate envelope maps for *Lasioglossum pauxillum*. Showing climate envelope for 1980-89 **(A)**, 2010-19 **(B)**, and 2070-79 under RCP 4.5 **(C)** and RCP 8.5 **(D)**.
10th percentile training presence cloglog threshold = 0.3318

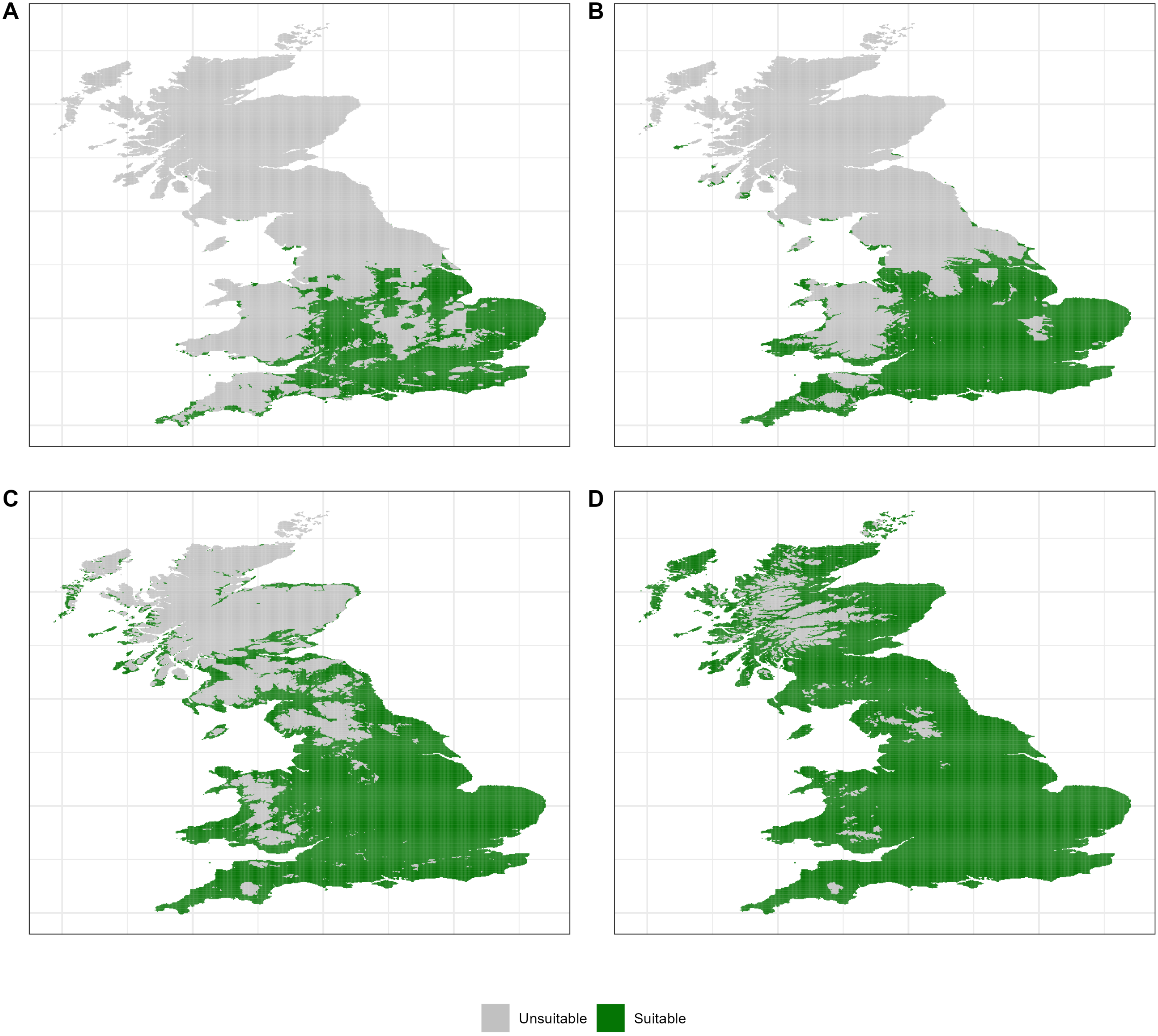

Figure S1. MaxEnt climate envelope maps for *Lasioglossum smeathmanellum*. Showing climate envelope for 1980-89 **(A)**, 2010-19 **(B)**, and 2070-79 under RCP 4.5 **(C)** and RCP 8.5 **(D)**.
10th percentile training presence cloglog threshold = 0.2869

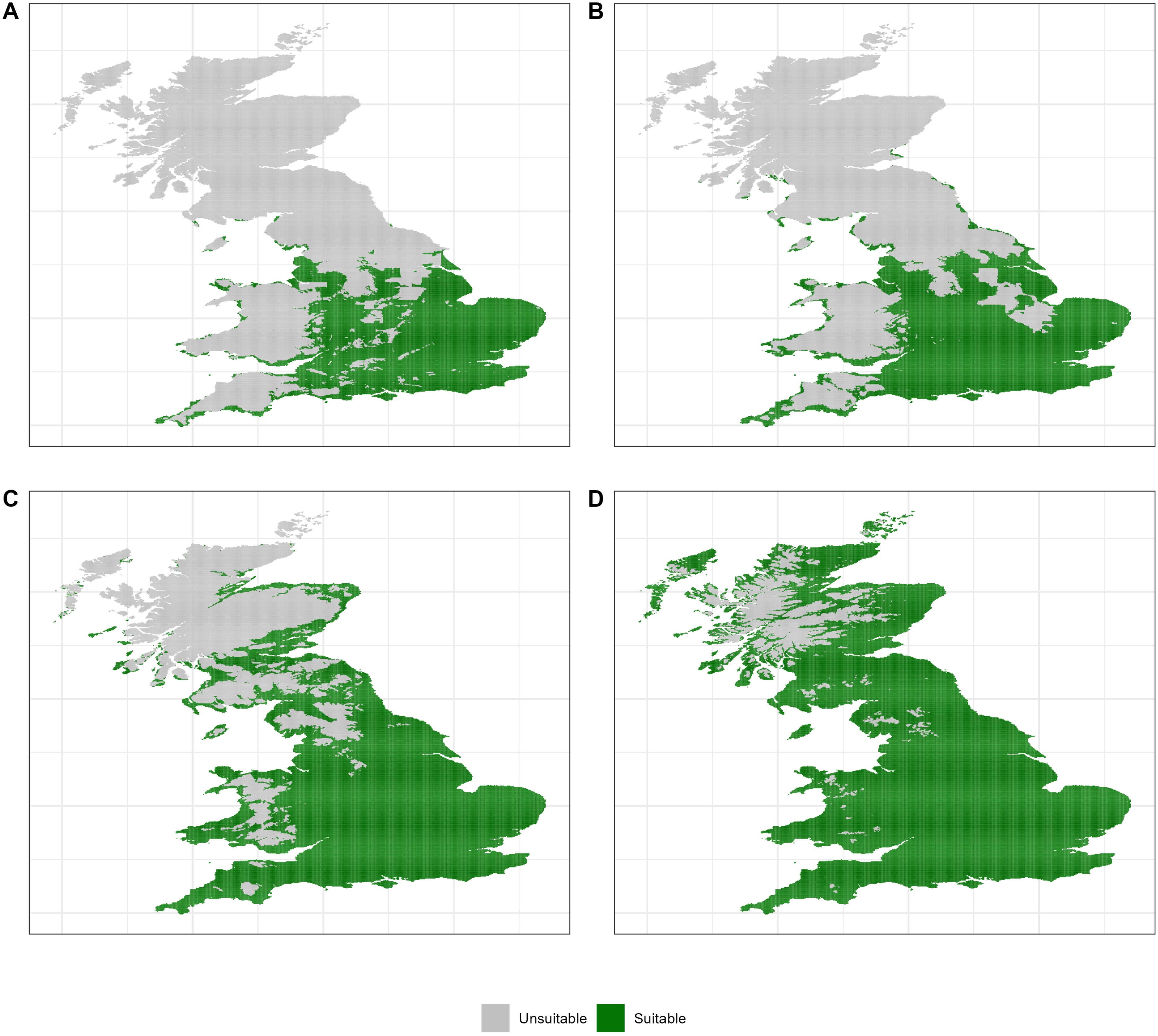

Figure S1. MaxEnt climate envelope maps for *Lasioglossum villosulum*. Showing climate envelope for 1980-89 **(A)**, 2010-19 **(B)**, and 2070-79 under RCP 4.5 **(C)** and RCP 8.5 **(D)**.
10th percentile training presence cloglog threshold = 0.2739

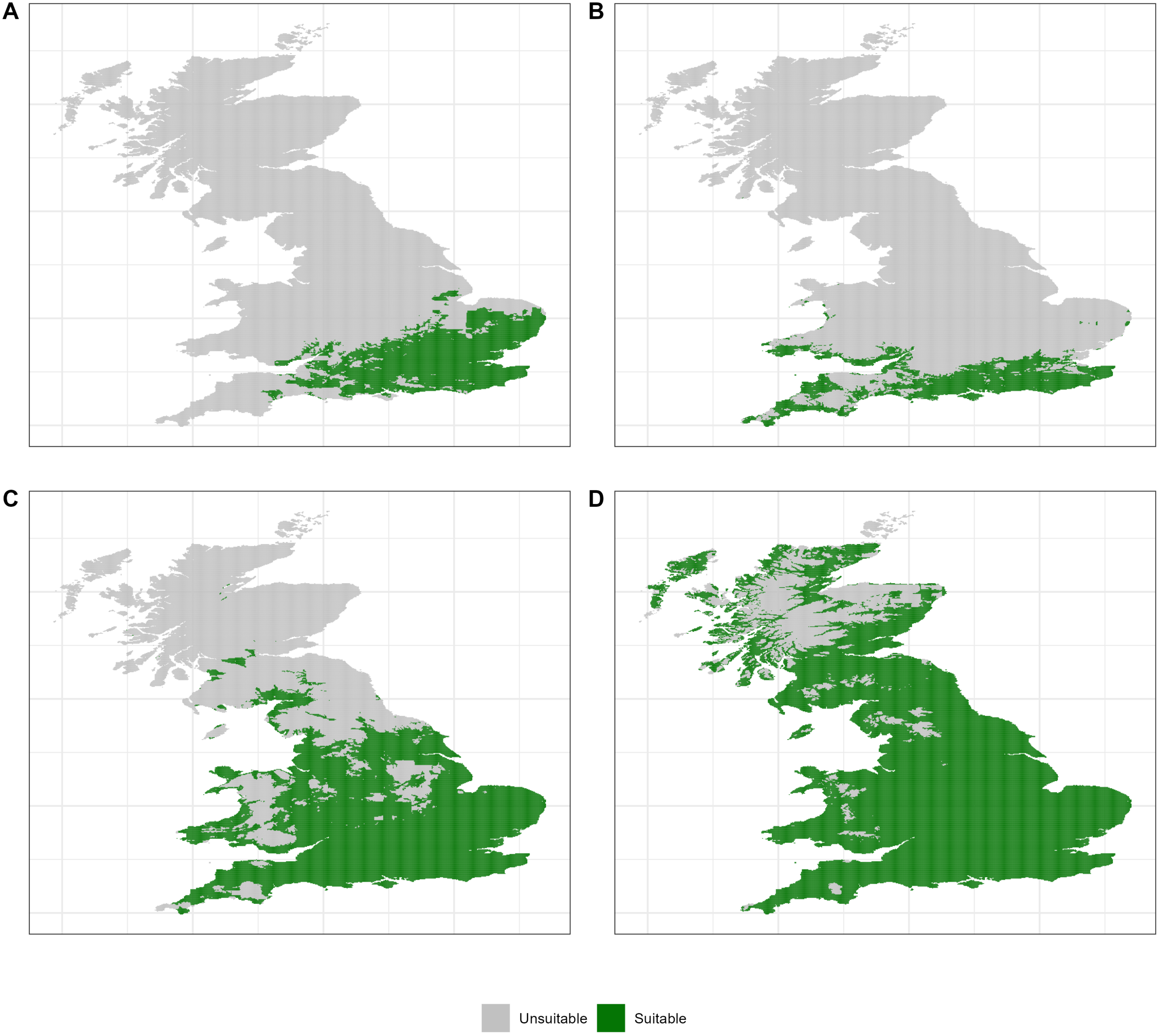

Figure S1. MaxEnt climate envelope maps for *Lasioglossum zonulum*. Showing climate envelope for 1980-89 **(A)**, 2010-19 **(B)**, and 2070-79 under RCP 4.5 **(C)** and RCP 8.5 **(D)**.
10th percentile training presence cloglog threshold = 0.2777

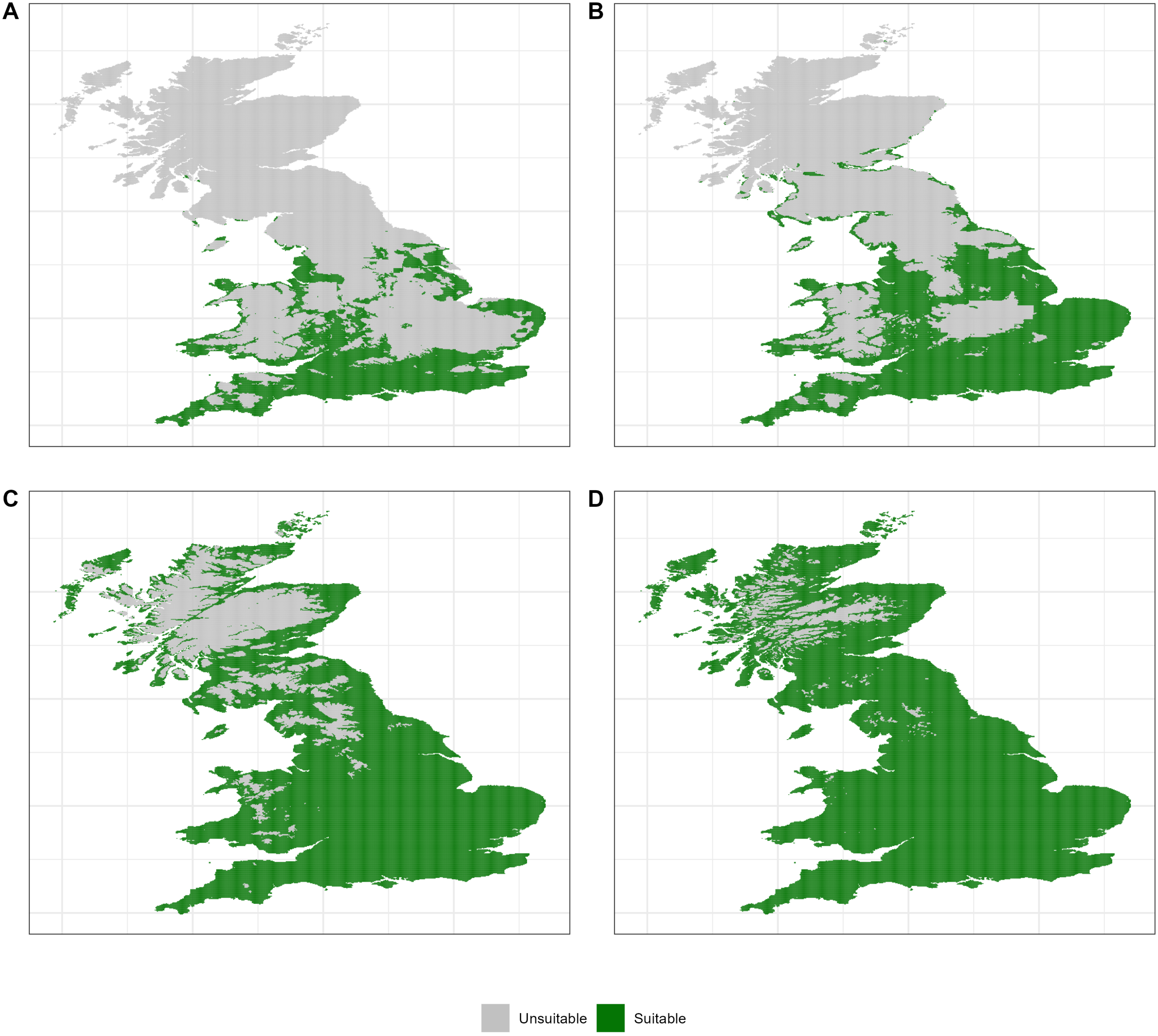

Figure S1. MaxEnt climate envelope maps for *Megachile centuncularis*. Showing climate envelope for 1980-89 **(A)**, 2010-19 **(B)**, and 2070-79 under RCP 4.5 **(C)** and RCP 8.5 **(D)**.
10th percentile training presence cloglog threshold = 0.2936

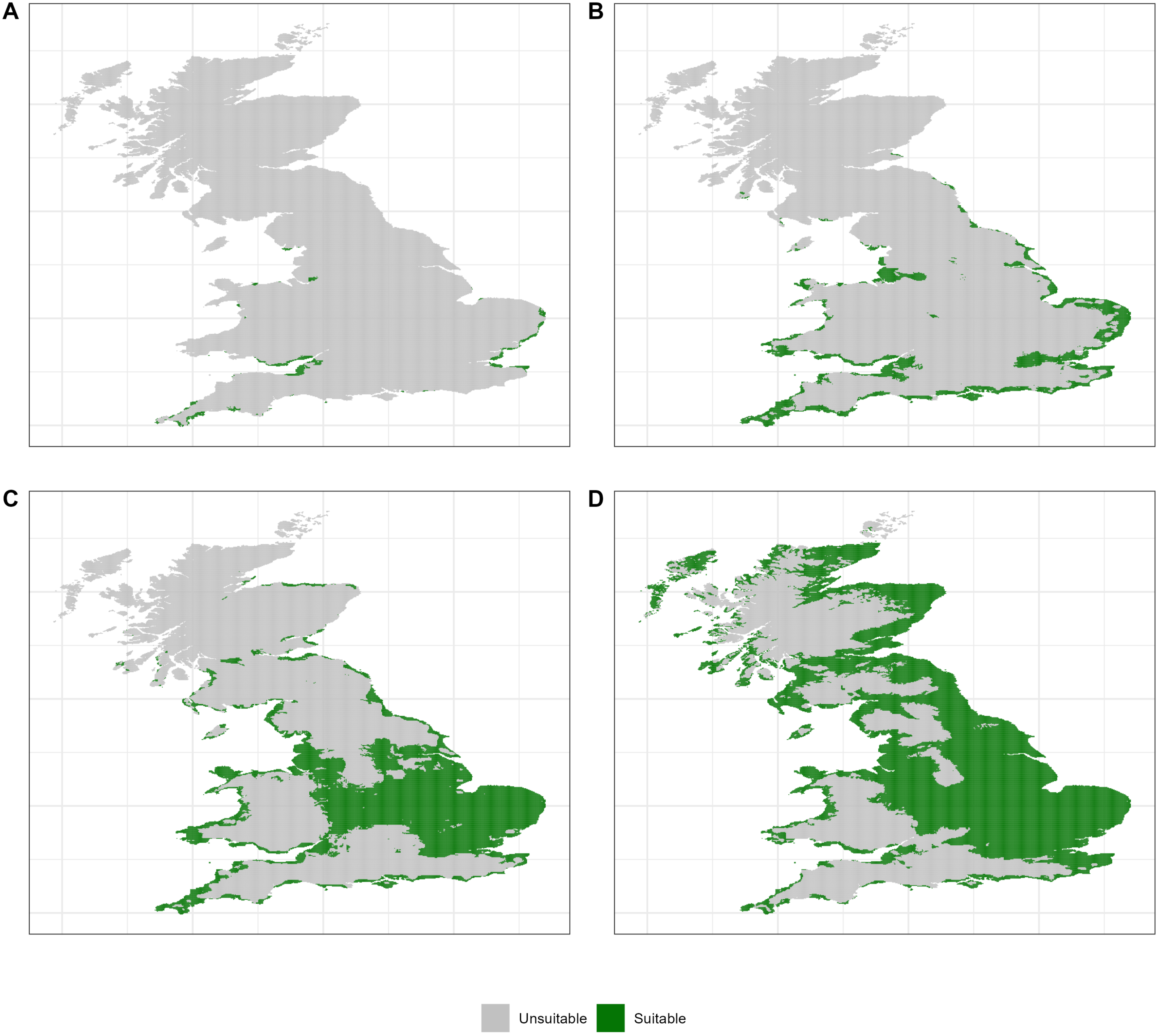

Figure S1. MaxEnt climate envelope maps for *Megachile leachella*. Showing climate envelope for 1980-89 **(A)**, 2010-19 **(B)**, and 2070-79 under RCP 4.5 **(C)** and RCP 8.5 **(D)**.
10th percentile training presence cloglog threshold = 0.1361

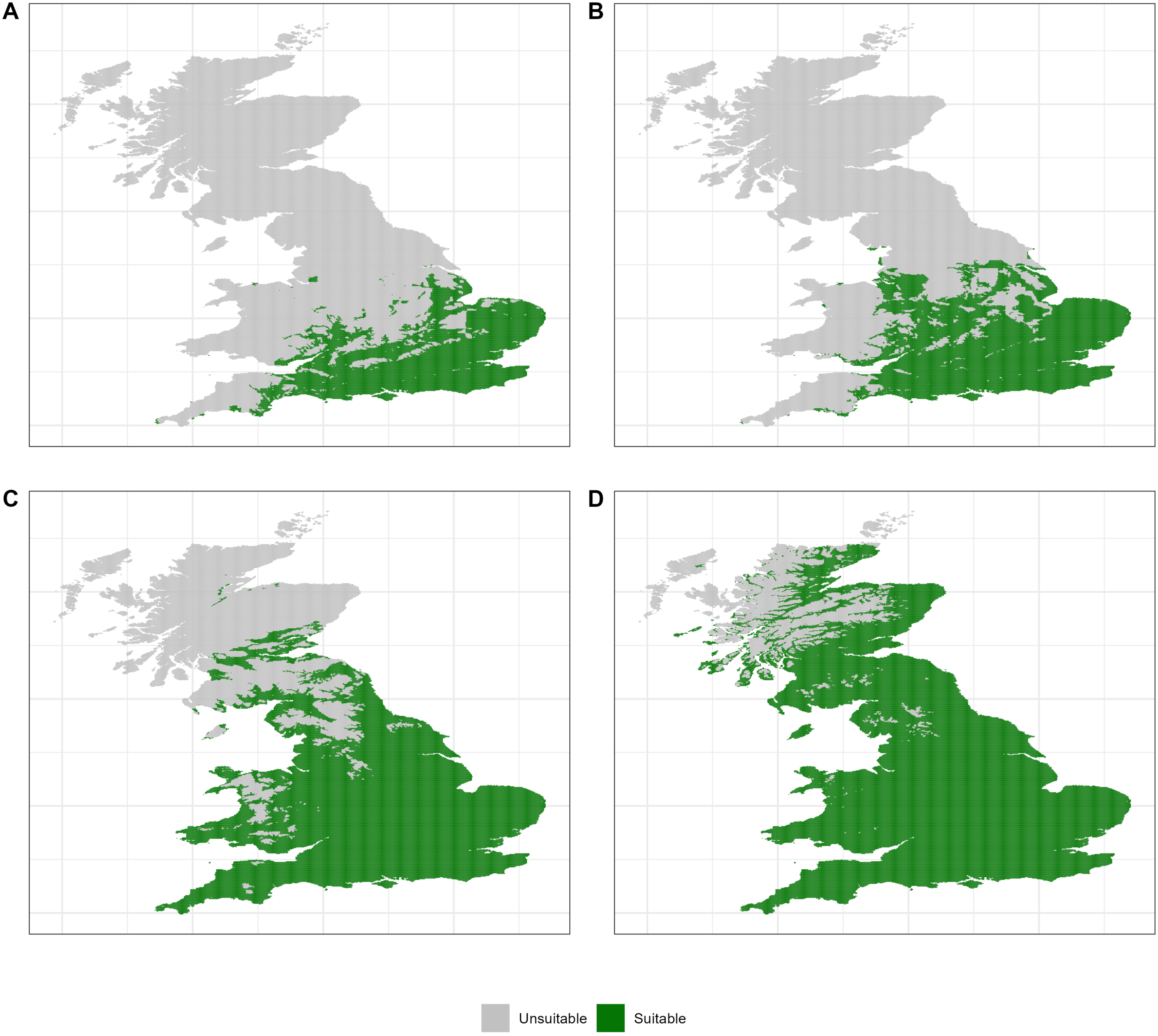

Figure S1. MaxEnt climate envelope maps for *Megachile ligniseca*. Showing climate envelope for 1980-89 **(A)**, 2010-19 **(B)**, and 2070-79 under RCP 4.5 **(C)** and RCP 8.5 **(D)**.
10th percentile training presence cloglog threshold = 0.3624

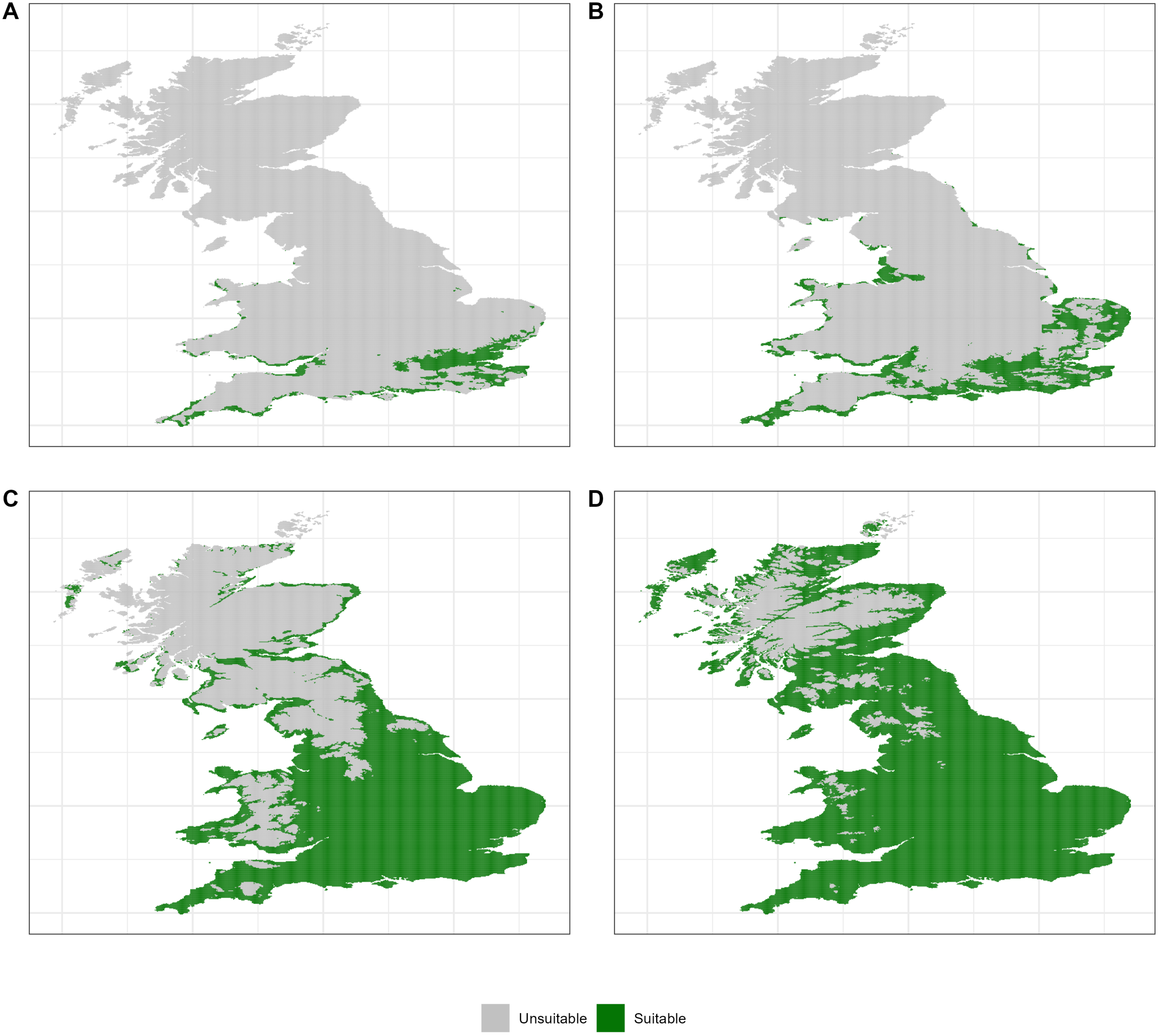

Figure S1. MaxEnt climate envelope maps for *Megachile maritima*. Showing climate envelope for 1980-89 **(A)**, 2010-19 **(B)**, and 2070-79 under RCP 4.5 **(C)** and RCP 8.5 **(D)**.
10th percentile training presence cloglog threshold = 0.2611

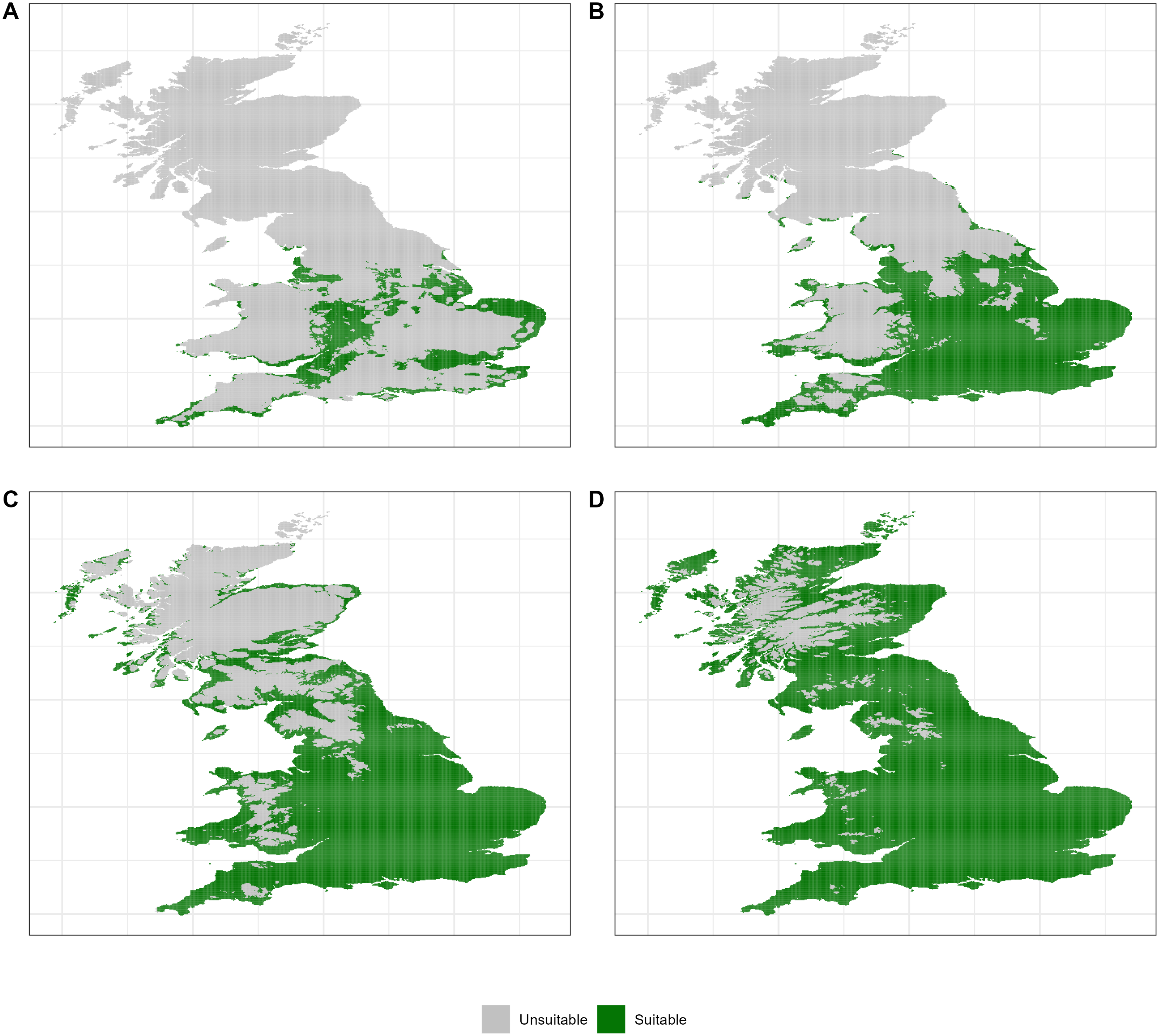

Figure S1. MaxEnt climate envelope maps for *Megachile versicolor*. Showing climate envelope for 1980-89 **(A)**, 2010-19 **(B)**, and 2070-79 under RCP 4.5 **(C)** and RCP 8.5 **(D)**.
10th percentile training presence cloglog threshold = 0.3356

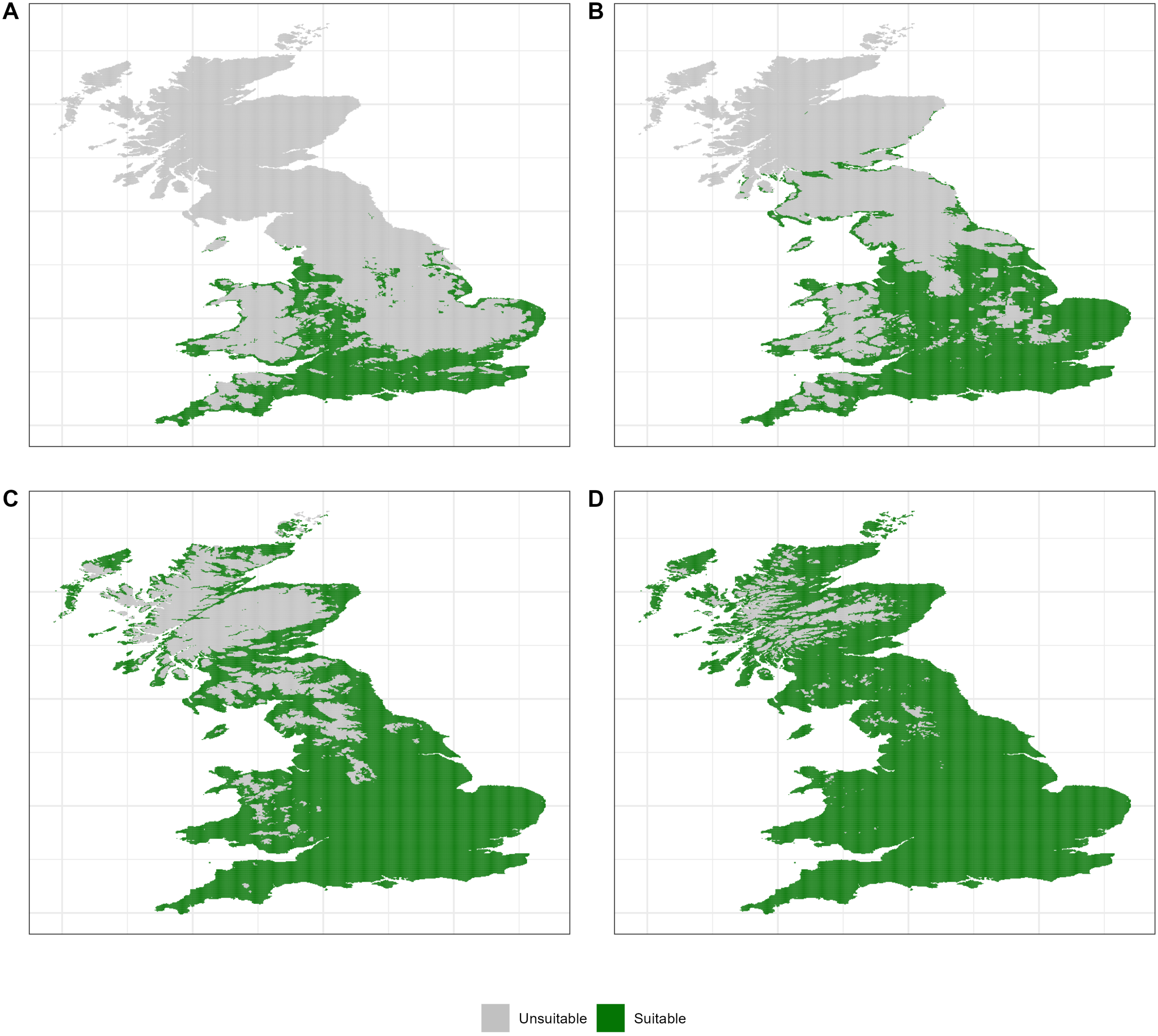

Figure S1. MaxEnt climate envelope maps for *Megachile willughbiella*. Showing climate envelope for 1980-89 **(A)**, 2010-19 **(B)**, and 2070-79 under RCP 4.5 **(C)** and RCP 8.5 **(D)**.
10th percentile training presence cloglog threshold = 0.3412

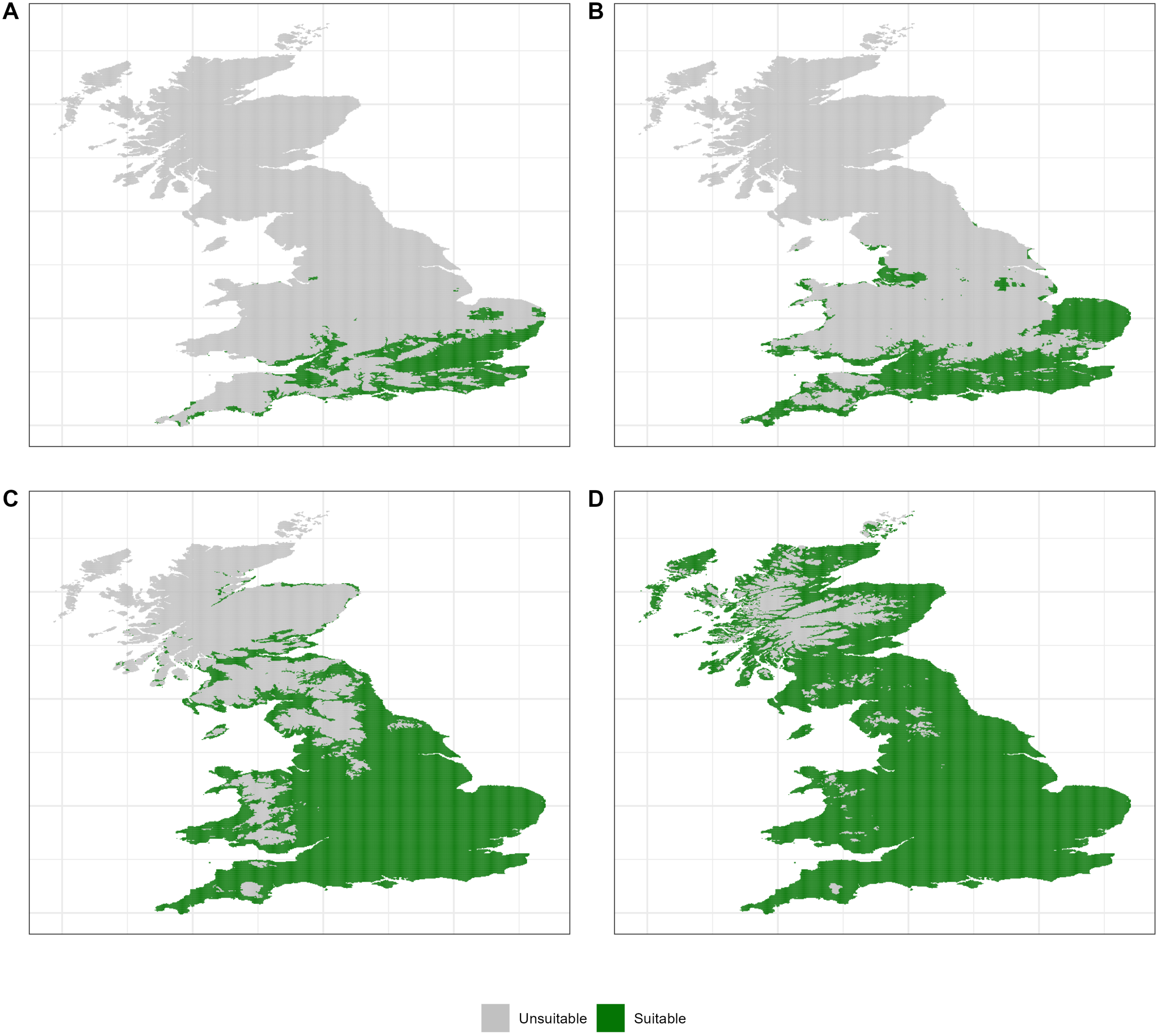

Figure S1. MaxEnt climate envelope maps for *Melitta leporina*. Showing climate envelope for 1980-89 **(A)**, 2010-19 **(B)**, and 2070-79 under RCP 4.5 **(C)** and RCP 8.5 **(D)**.
10th percentile training presence cloglog threshold = 0.2094

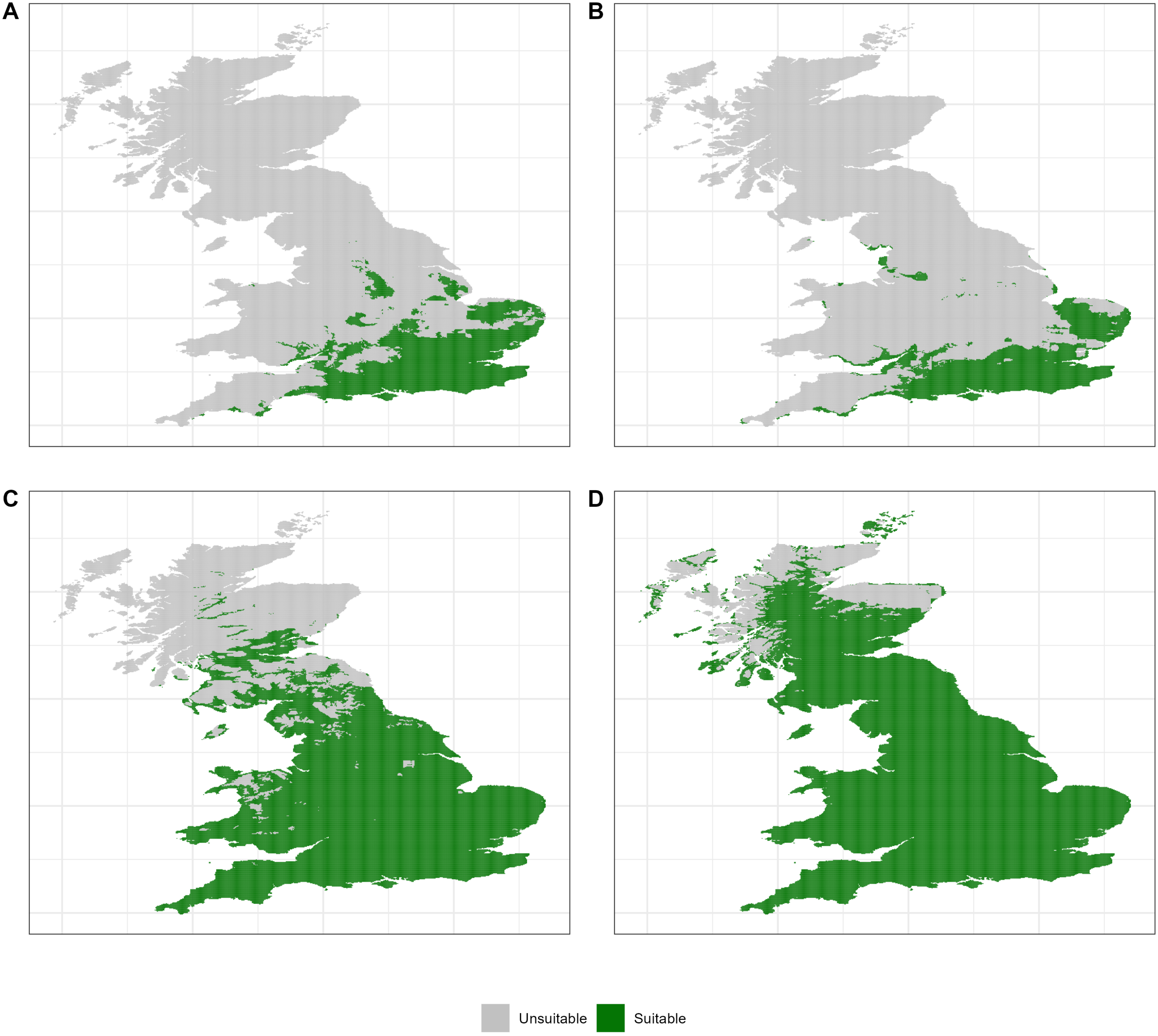

Figure S1. MaxEnt climate envelope maps for *Melitta tricincta*. Showing climate envelope for 1980-89 **(A)**, 2010-19 **(B)**, and 2070-79 under RCP 4.5 **(C)** and RCP 8.5 **(D)**.
10th percentile training presence cloglog threshold = 0.2551

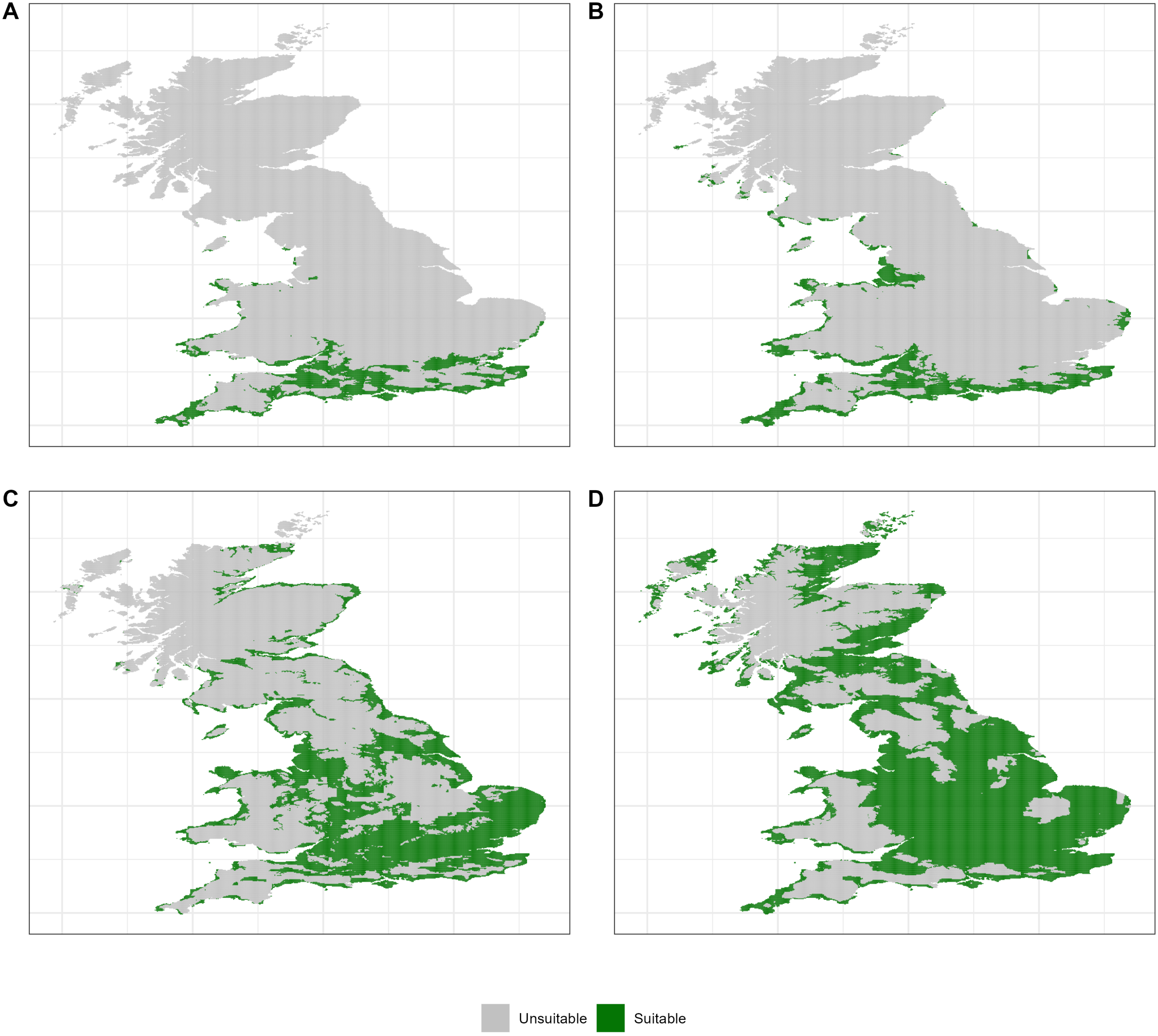

Figure S1. MaxEnt climate envelope maps for *Osmia aurulenta*. Showing climate envelope for 1980-89 **(A)**, 2010-19 **(B)**, and 2070-79 under RCP 4.5 **(C)** and RCP 8.5 **(D)**.
10th percentile training presence cloglog threshold = 0.156

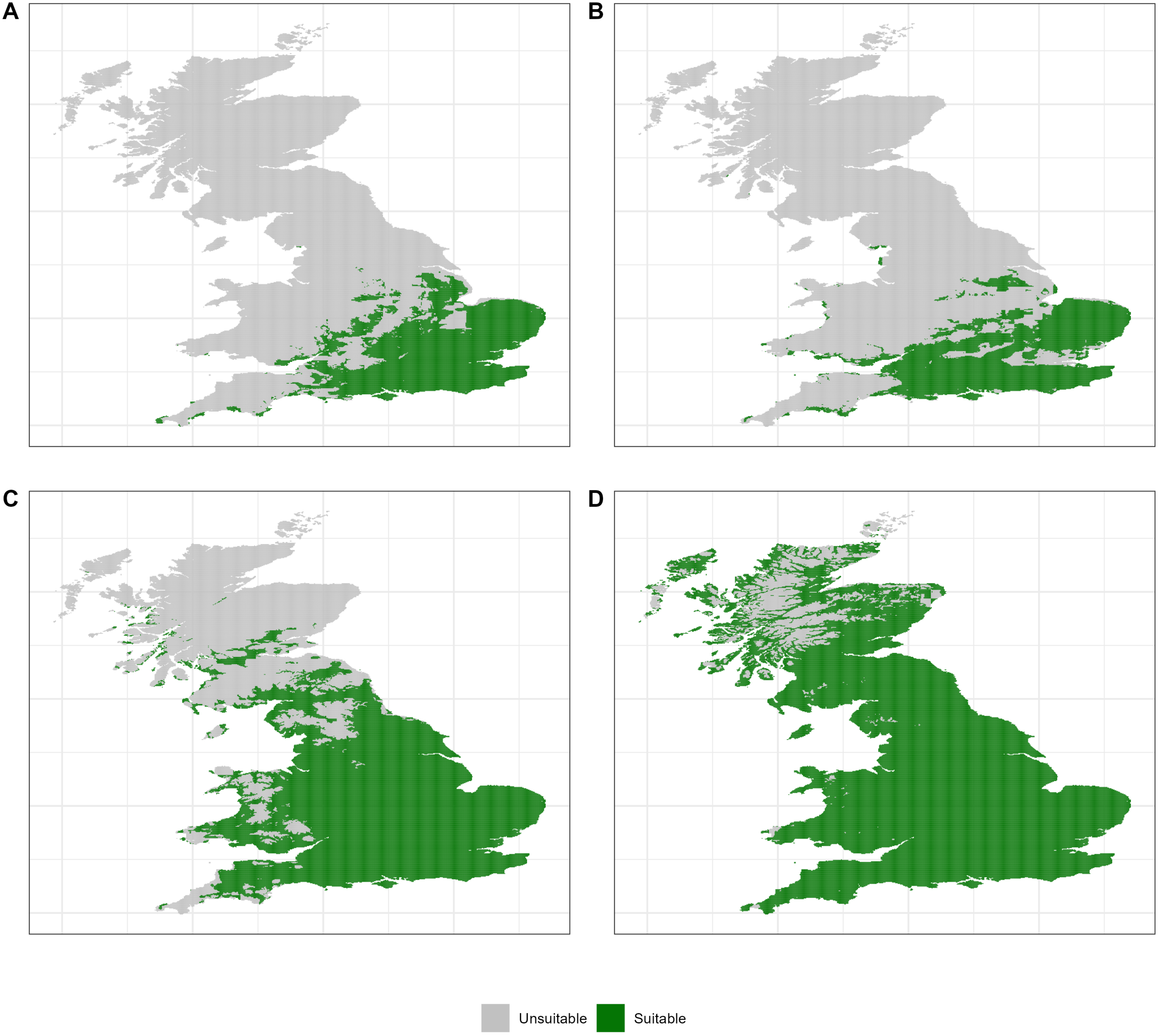

Figure S1. MaxEnt climate envelope maps for *Osmia bicolor*. Showing climate envelope for 1980-89 **(A)**, 2010-19 **(B)**, and 2070-79 under RCP 4.5 **(C)** and RCP 8.5 **(D)**.
10th percentile training presence cloglog threshold = 0.3248

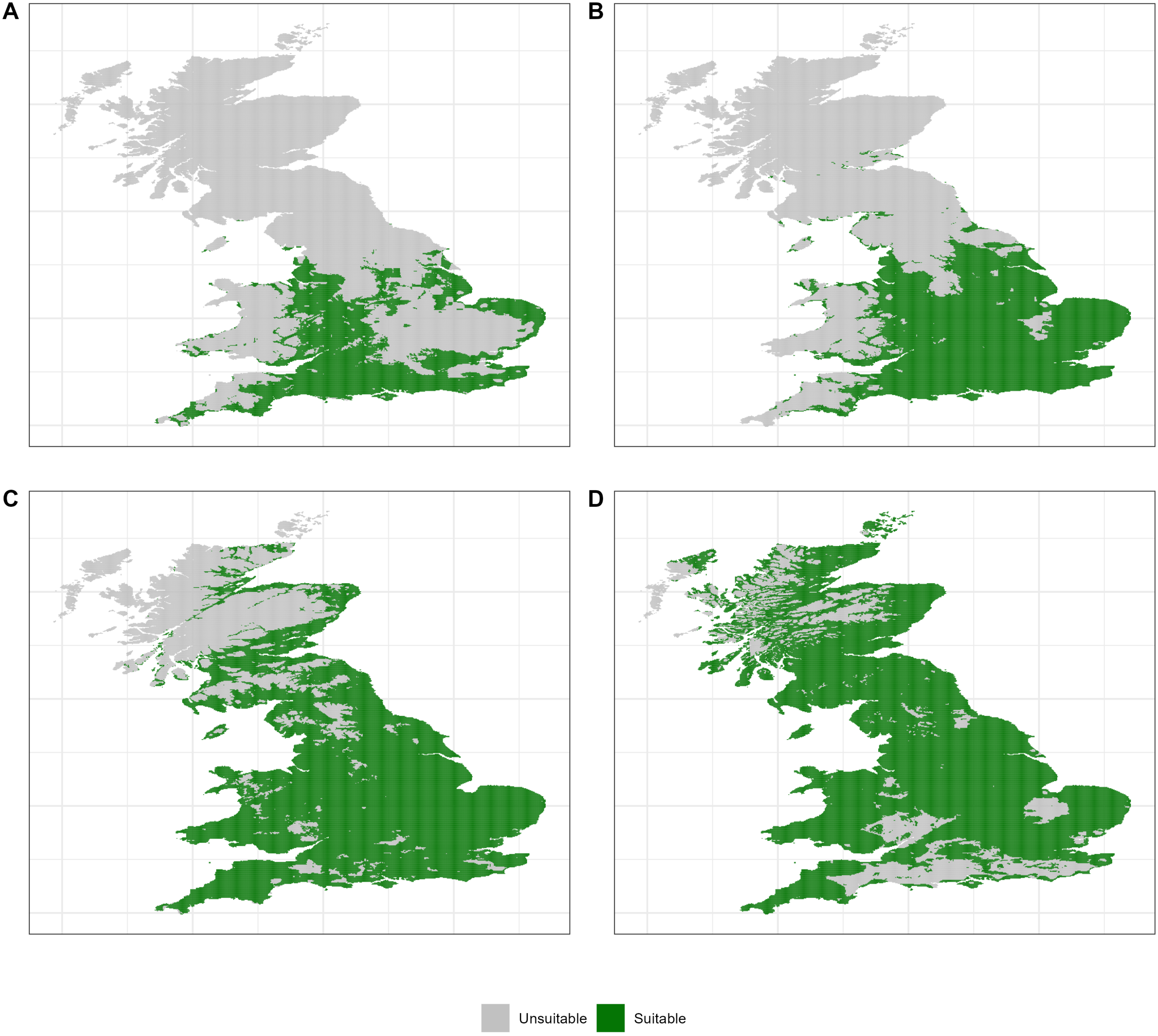

Figure S1. MaxEnt climate envelope maps for *Osmia bicornis*. Showing climate envelope for 1980-89 **(A)**, 2010-19 **(B)**, and 2070-79 under RCP 4.5 **(C)** and RCP 8.5 **(D)**.
10th percentile training presence cloglog threshold = 0.3684

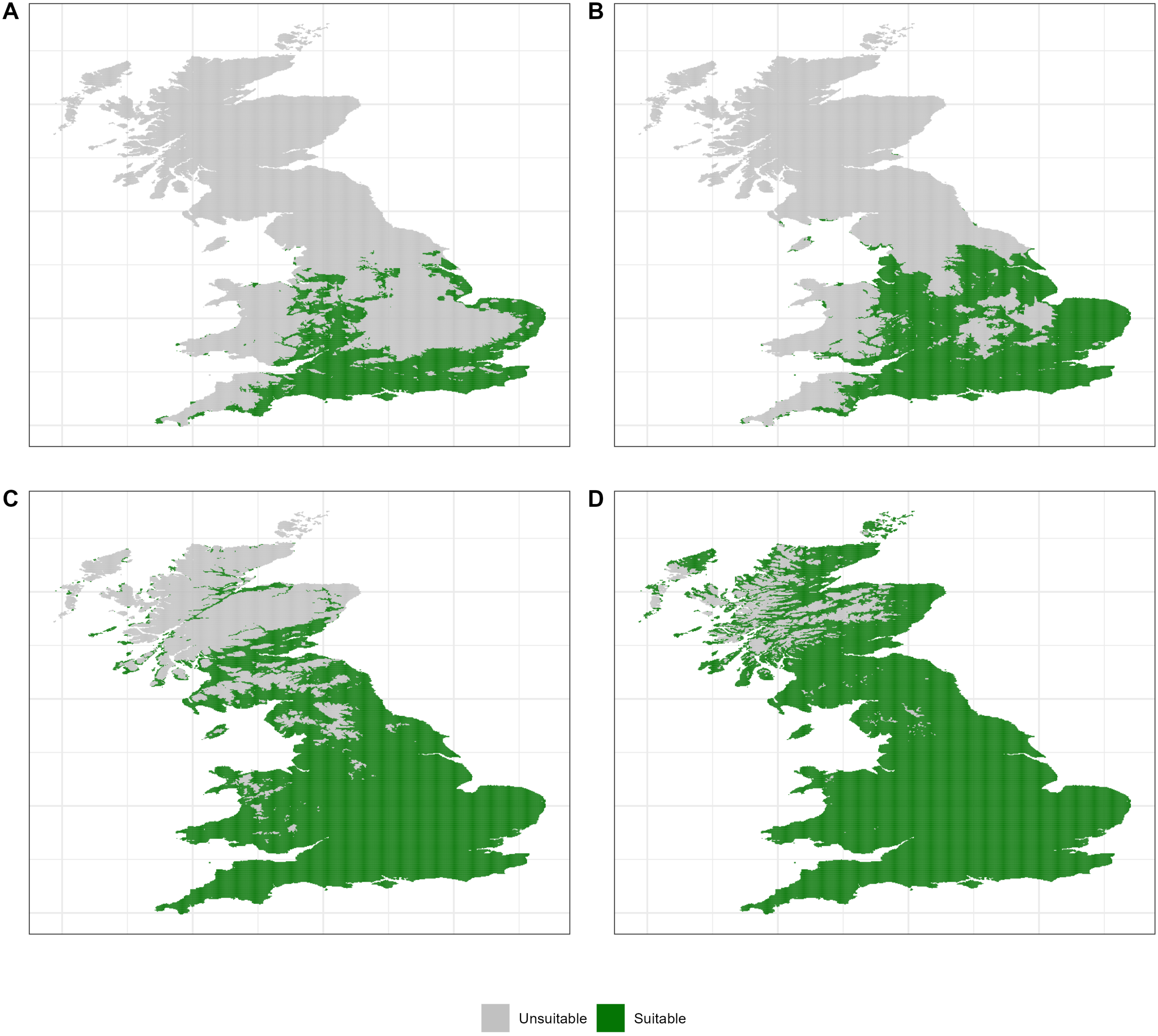

Figure S1. MaxEnt climate envelope maps for *Osmia caerulescens*. Showing climate envelope for 1980-89 **(A)**, 2010-19 **(B)**, and 2070-79 under RCP 4.5 **(C)** and RCP 8.5 **(D)**.
10th percentile training presence cloglog threshold = 0.3401

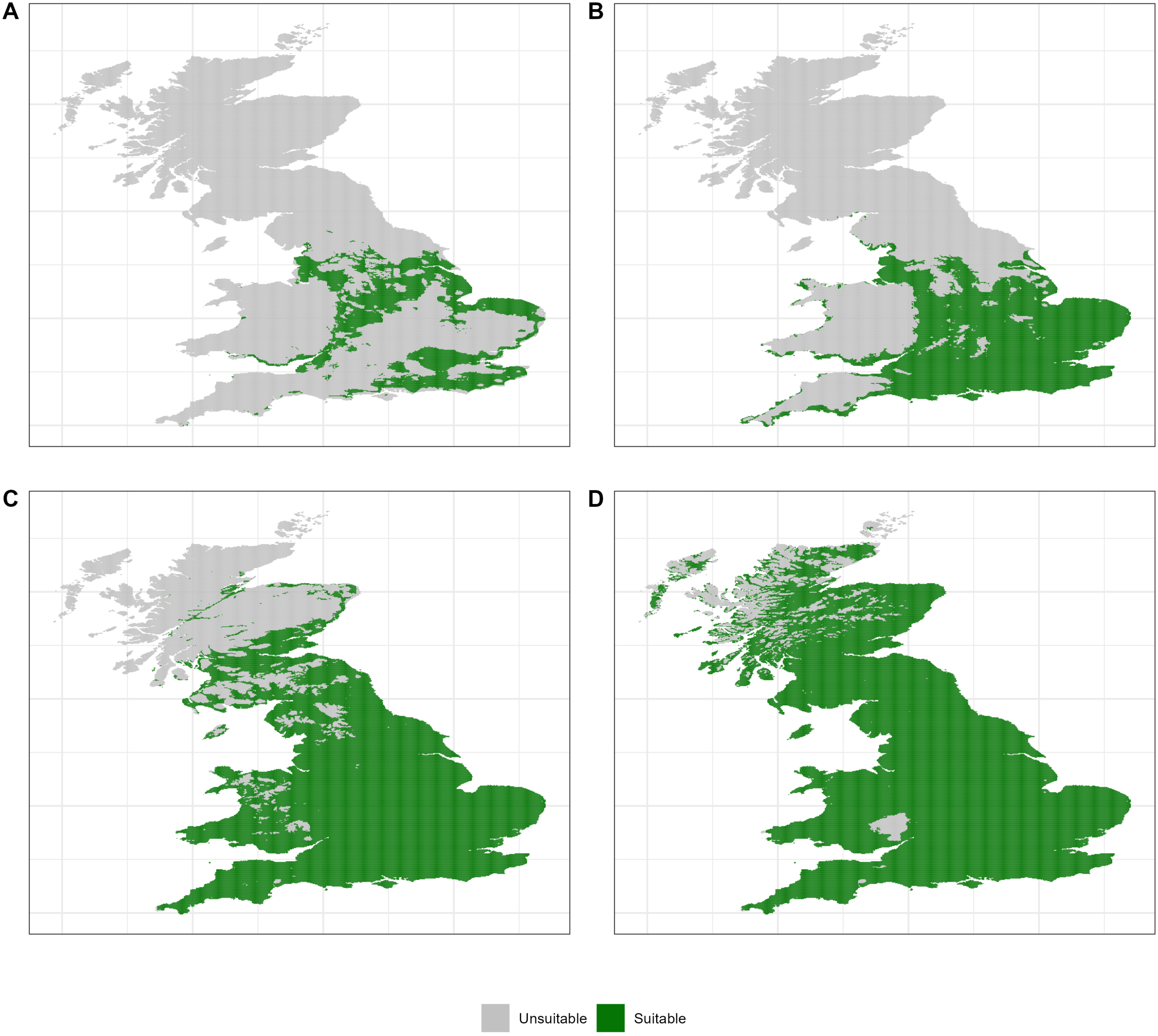

Figure S1. MaxEnt climate envelope maps for *Sphecodes crassus*. Showing climate envelope for 1980-89 **(A)**, 2010-19 **(B)**, and 2070-79 under RCP 4.5 **(C)** and RCP 8.5 **(D)**.
10th percentile training presence cloglog threshold = 0.2738

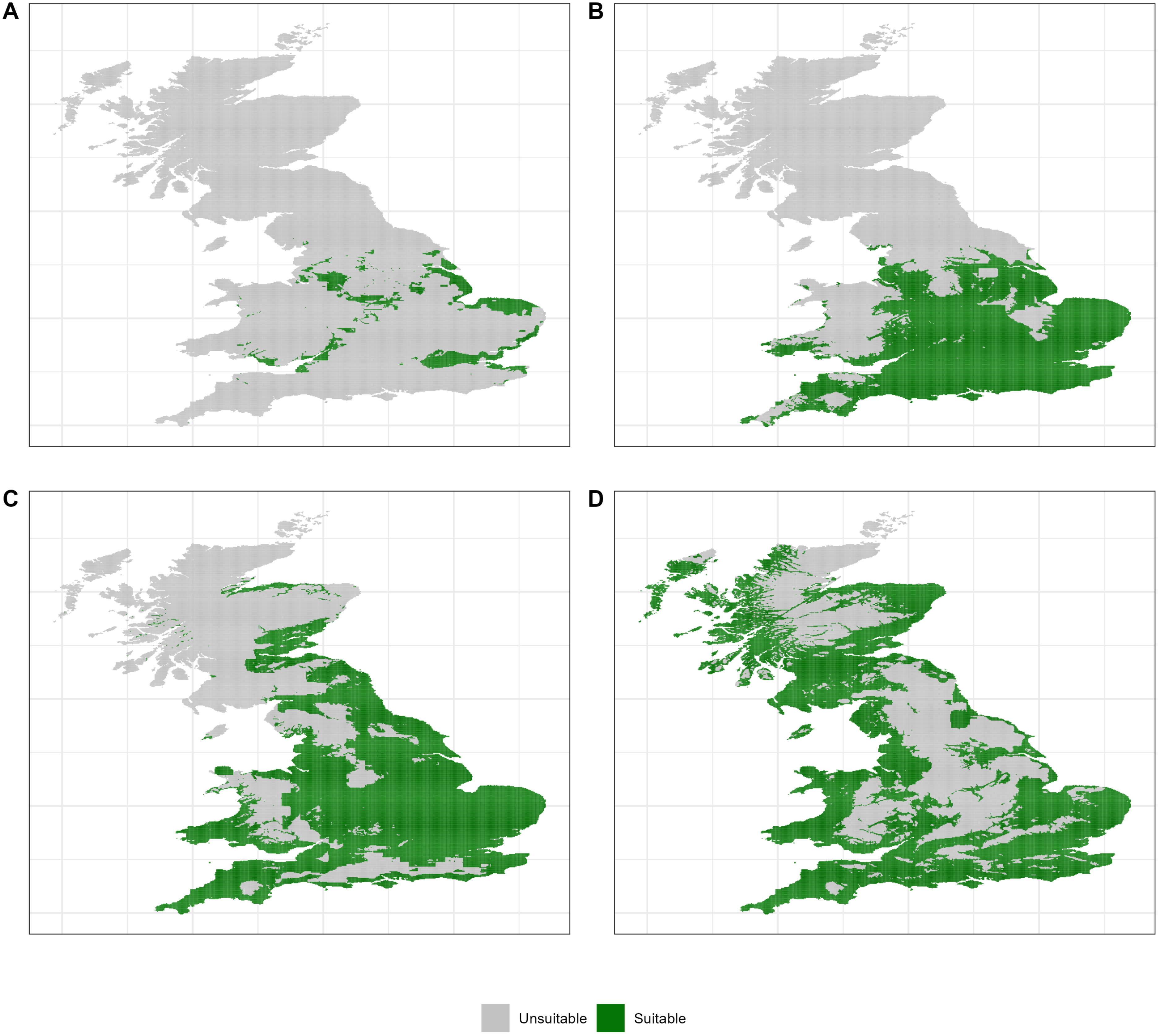

Figure S1. MaxEnt climate envelope maps for *Sphecodes ephippius*. Showing climate envelope for 1980-89 **(A)**, 2010-19 **(B)**, and 2070-79 under RCP 4.5 **(C)** and RCP 8.5 **(D)**.
10th percentile training presence cloglog threshold = 0.3275

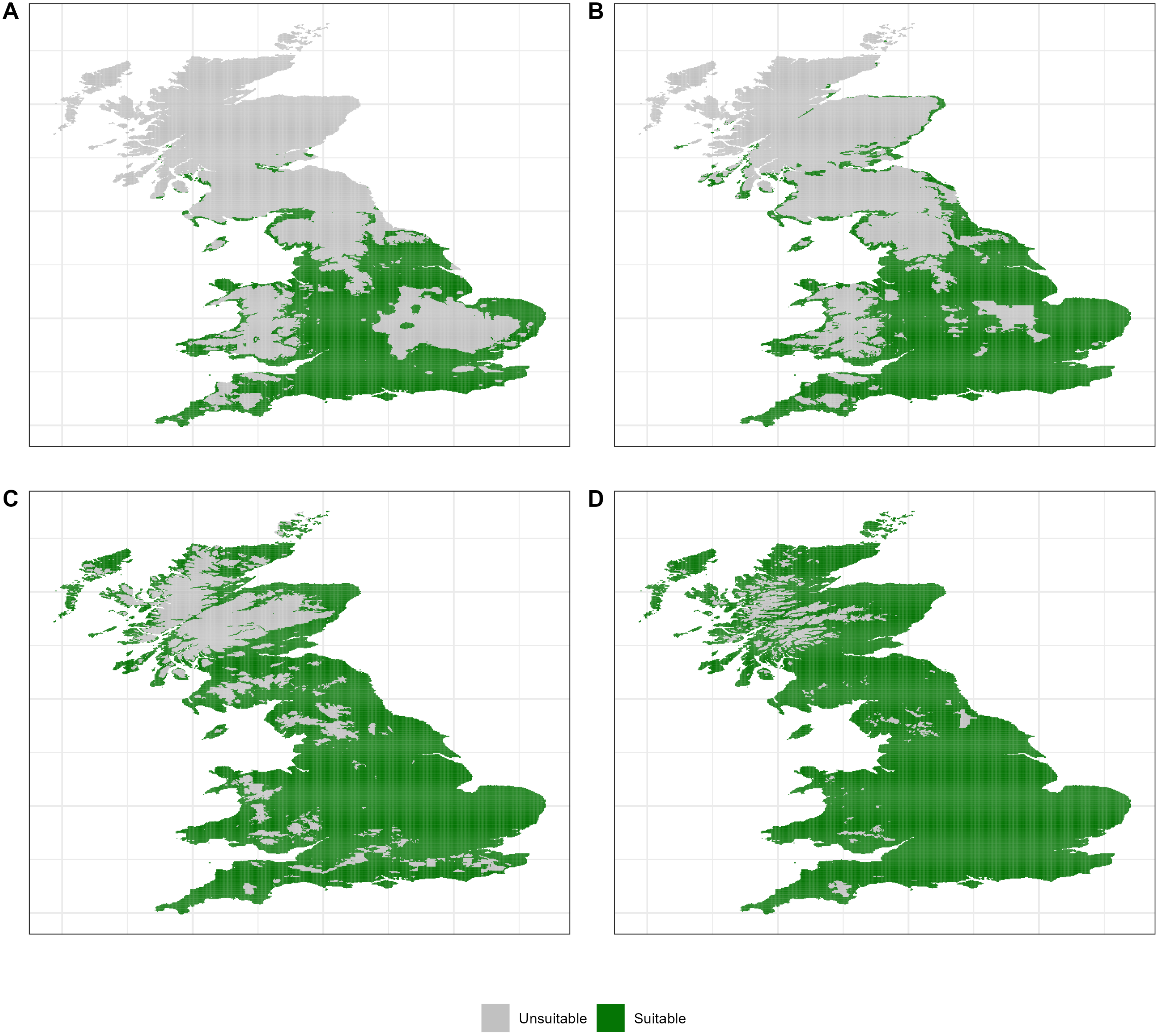

Figure S1. MaxEnt climate envelope maps for *Sphecodes geoffrellus*. Showing climate envelope for 1980-89 **(A)**, 2010-19 **(B)**, and 2070-79 under RCP 4.5 **(C)** and RCP 8.5 **(D)**.
10th percentile training presence cloglog threshold = 0.2727

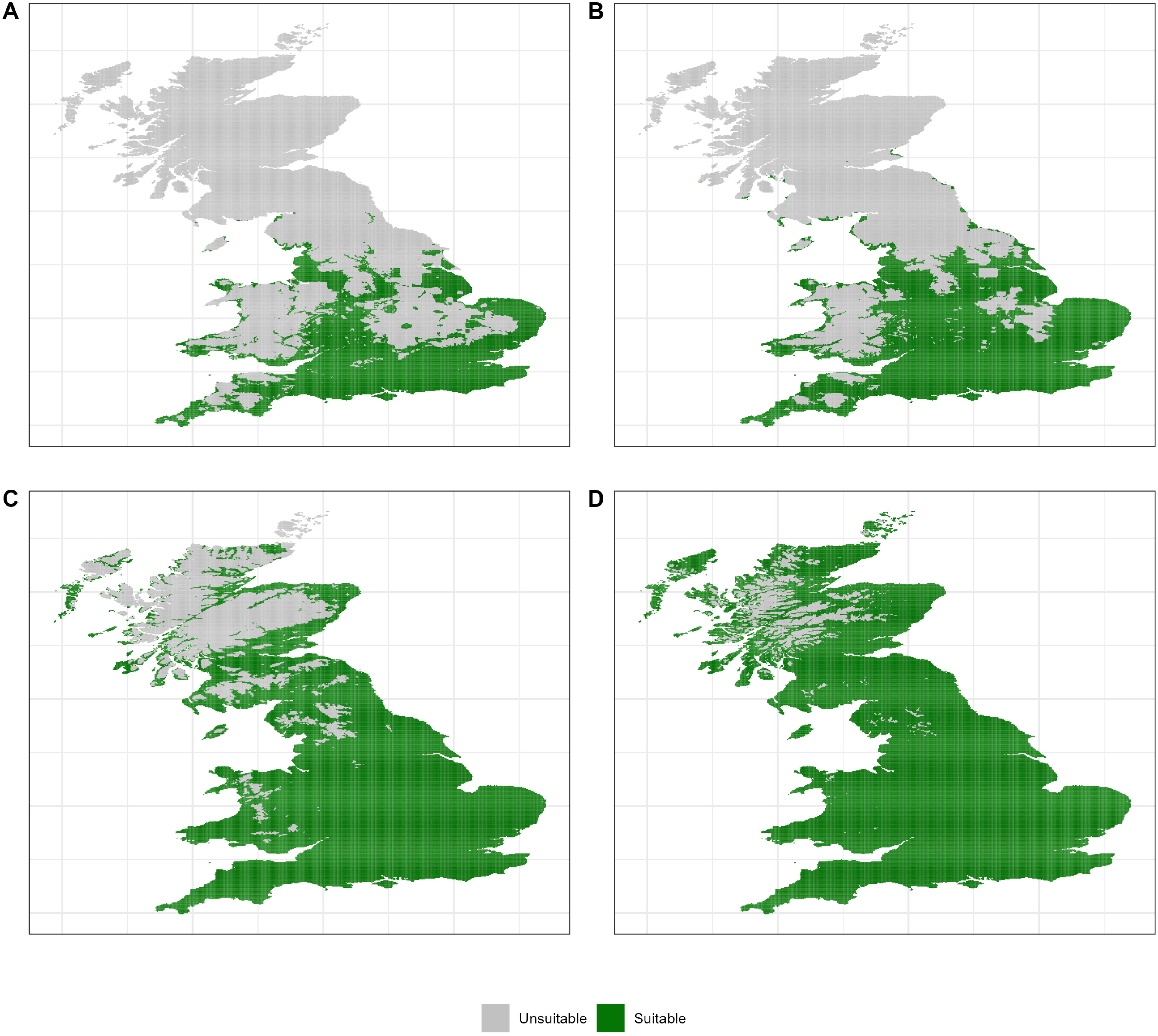

Figure S1. MaxEnt climate envelope maps for *Sphecodes monilicornis*. Showing climate envelope for 1980-89 **(A)**, 2010-19 **(B)**, and 2070-79 under RCP 4.5 **(C)** and RCP 8.5 **(D)**.
10th percentile training presence cloglog threshold = 0.288

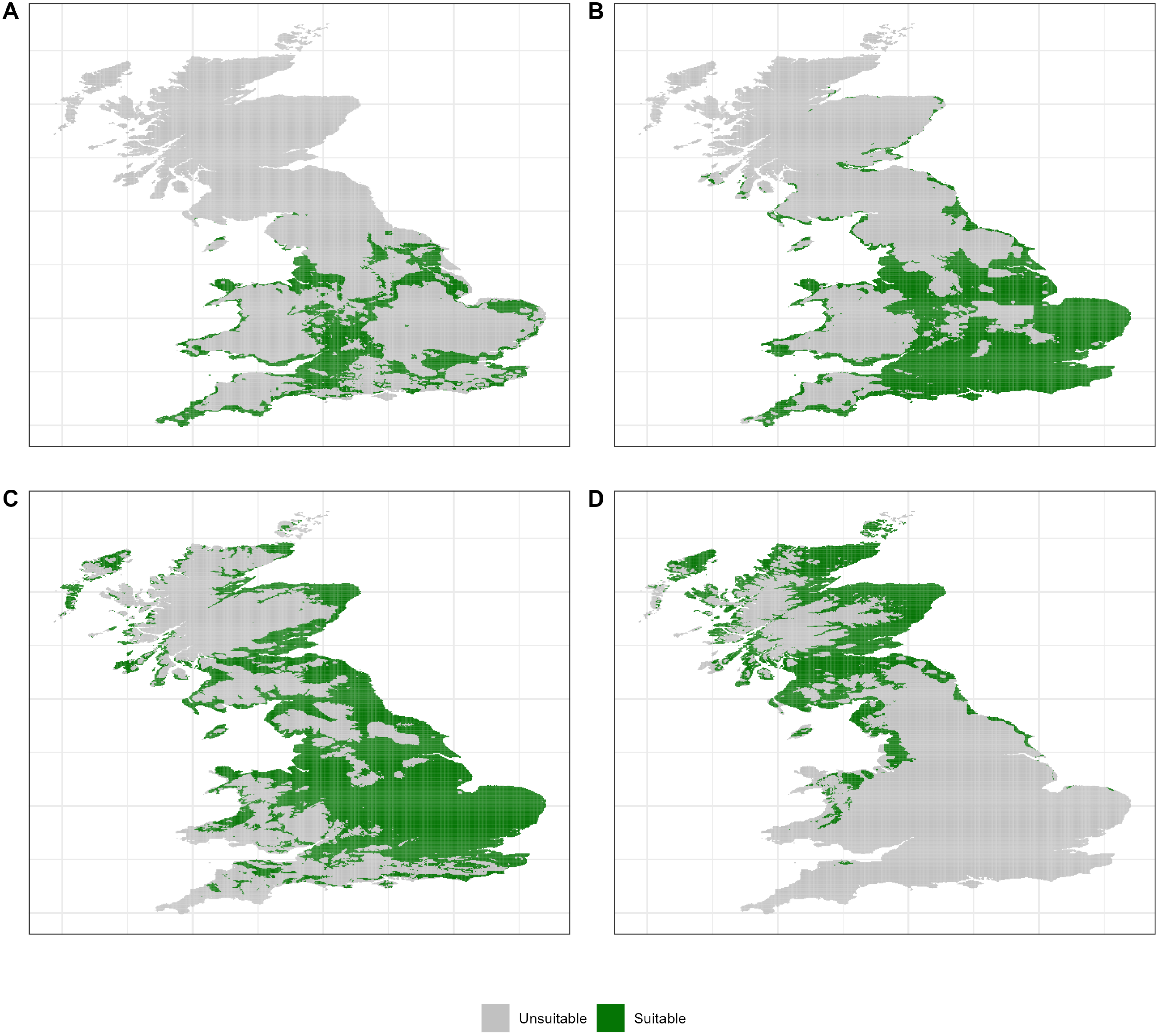

Figure S1. MaxEnt climate envelope maps for *Sphecodes pellucidus*. Showing climate envelope for 1980-89 **(A)**, 2010-19 **(B)**, and 2070-79 under RCP 4.5 **(C)** and RCP 8.5 **(D)**.
10th percentile training presence cloglog threshold = 0.2598

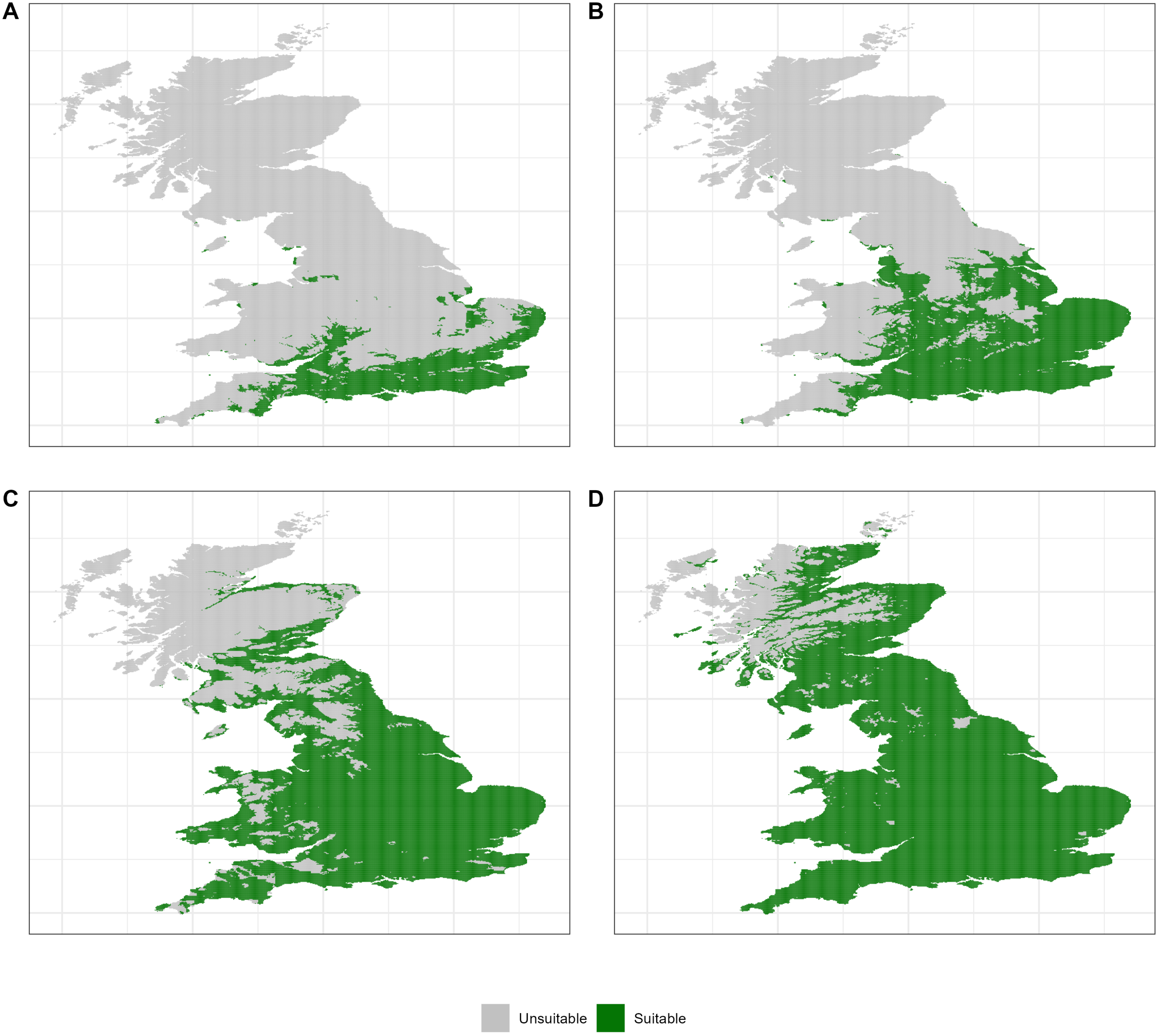

Figure S1. MaxEnt climate envelope maps for *Sphecodes puncticeps*. Showing climate envelope for 1980-89 **(A)**, 2010-19 **(B)**, and 2070-79 under RCP 4.5 **(C)** and RCP 8.5 **(D)**.
10th percentile training presence cloglog threshold = 0.3007
